# The impact of human immunodeficiency virus coinfection on mpox patients during the 2022 global outbreak: a systematic review and meta-analysis based on comparative observational studies

**DOI:** 10.3389/fmicb.2026.1751445

**Published:** 2026-02-09

**Authors:** Yingying Han, Xingzhao Li, Xin Wang, Zhuan Zhong

**Affiliations:** 1 Department of Neurology, China-Japan Union Hospital of Jilin University, Changchun, Jilin, China; 2 Department of Ultrasound, China-Japan Union Hospital of Jilin University, Changchun, Jilin, China; 3 Infection Management Department of Hospital, China-Japan Union Hospital of Jilin University, Changchun, Jilin, China; 4 Department of Orthopaedics, The Second Hospital of Jilin University, Changchun, Jilin, China

**Keywords:** comparative observational studies, HIV, meta-analysis, mpox, systematic review

## Abstract

**Background:**

The mpox outbreak in 2022 posed a new challenge to the medical system. We aimed to study the impact of human immunodeficiency virus (HIV) coinfection on mpox patients.

**Methods:**

This study was conducted in accordance with the Preferred Reporting Items for Systematic Reviews and Meta-Analyses (PRISMA) guidelines.

**Results:**

Our study included 27 articles for meta-analysis and divided mpox patients into two groups: one group was HIV-positive, and the other group was HIV-negative. We found that the age of HIV-positive patients was significantly greater than that of HIV-negative patients (SMD = 0.35, 95% CI: 0.29–0.41) and that there were more men who had sex with men in the HIV-positive group (OR = 3.98, 95% CI: 3.04–5.21). Syphilis (OR = 2.66, 95% CI: 2.13–3.33), hepatitis B (OR = 3.94, 95% CI: 2.93–5.30), hepatitis C (OR = 5.71, 95% CI: 3.06–10.64), proctitis (OR = 1.52, 95% CI: 1.17–1.98), fever (OR = 1.15, 95% CI: 1.01–1.30), diarrhea (OR = 1.69, 95% CI: 1.03–2.77) and pustules (OR = 1.33, 95% CI: 1.08–1.62) were more common among HIV-positive patients. HIV coinfection seemed to be associated with a decrease in the CD4^+^ T-cell count (SMD = −0.78, 95% CI: −1.34 to −0.23) and hemoglobin (SMD = −0.43, 95% CI: −0.64 to −0.22) and albumin (SMD = −0.35, 95% CI: −0.55 to −0.15) levels, whereas the CD8^+^ T-cell count yielded the opposite statistical conclusion (SMD = 0.35, 95% CI: 0.03–0.67). We also found that HIV-positive patients had more hospitalizations (OR = 1.63, 95% CI: 1.22–2.19), more severe mpox (OR = 1.82, 95% CI: 1.28–2.58), more need for tecovirimat treatment (OR = 4.25, 95% CI: 1.59–11.4), and higher mortality (OR = 3.36, 95% CI: 1.15–9.83).

**Conclusion:**

Overall, HIV coinfection may influence the disease process and clinical indicators of mpox patients and is associated with more severe clinical outcomes.

**Systematic review registration:**

https://www.crd.york.ac.uk/PROSPERO/view/CRD420251056729, PROSPERO registration number: CRD420251056729.

## 1 Introduction

Mpox is a zoonotic infectious disease caused by the mpox virus, an enveloped double-stranded DNA virus belonging to the Orthopoxvirus genus of the Poxviridae family, which also includes variola and vaccinia viruses (Parker et al., 2007). The first case of human infection with the mpox virus was observed in a 9-year-old boy in 1970 in the Democratic Republic of Congo (Ladnyj et al., 1972). Since then, sporadic outbreaks outside Central and West African countries have been limited to a few cases in recent years and have been based on zoonotic transmission or exported cases from endemic countries (Simpson et al., 2020). On May 6, 2022, a British national who returned from Nigeria was diagnosed with mpox after developing a febrile rash; this marked the beginning of the 2022 outbreak, which spread to non-endemic countries around the world (Girometti et al., 2022). One of the possible origins and focuses of the current mpox outbreak was the Gay Pride Festival in Gran Canaria (May 5–15, 2022), which was attended by 25,000–30,000 visitors from abroad (Martinez et al., 2022). The World Health Organization (WHO) declared it a Public Health Emergency of International Concern in July 2022, which was later lifted on May 11, 2023 (World Health Organization, 2025a). The presence of mpox virus has been confirmed by polymerase chain reaction (PCR) in various secretions, such as saliva, semen, urine and nasopharyngeal or rectal samples (Tarin-Vicente et al., 2022; Peiro-Mestres et al., 2022). Fever, headache, myalgia, asthenia and lymphadenopathy are common symptoms and signs in the initial stage, with a rash appearing in the second phase and lasting for 2–3 weeks. Most mpox patients fully recover (World Health Organization, 2022a).

The WHO reported that human immunodeficiency virus (HIV) remains a major global public health problem, affecting more than 40 million people around the world. In 2022, when the mpox epidemic occurred, 630,000 people died of HIV-associated comorbidities, and 1.3 million people acquired HIV (World Health Organization, 2025b). According to data released by the WHO, approximately 50% of mpox patients were coinfected with HIV during this global epidemic (World Health Organization, 2025b). Notably, HIV prevalence among mpox cases differed substantially across regions. A study by Curran et al. reported that among mpox patients in eight jurisdictions in the United States, 38% were infected with HIV (Curran et al., 2022). In contrast, a national retrospective study conducted in Peru using the National Surveillance System, which included 3,561 confirmed mpox cases, found that up to 60% of patients were infected with HIV (Ramirez-Soto et al., 2024). HIV infection compromises the immune system, rendering individuals more susceptible to a series of opportunistic infections and affecting the epidemiology, clinical manifestations and treatment outcomes of concurrent diseases. Mpox virus was transmitted mainly through sexual behavior in the 2022 epidemic, which is more common among men who have sex with men (MSM). This mode of transmission is similar to that of HIV, resulting in a high proportion of patients living with HIV infection (Yang S. et al., 2024).

We carried out this systematic review and meta-analysis to study the influence of HIV coinfection on mpox patients and to provide a reference for the clinical diagnosis and treatment of such patients and the public health care of mpox patients. In the 2022 global epidemic, the transmission route, affected area, location of lesions and severity of disease were different from those of previous mpox epidemics (Thornhill et al., 2022; Yinka-Ogunleye et al., 2019; Ogoina et al., 2020), so we did not include data from mpox patients before 2022 in this study.

## 2 Methods

This study was conducted in accordance with the Preferred Reporting Items for Systematic Reviews and Meta-Analyses (PRISMA) statement (PROSPERO registration number: CRD420251056729).

### 2.1 Eligibility criteria

Articles that met the following requirements were included in our study: (1) all included patients had confirmed cases of mpox, (2) the clinical characteristics of the HIV-positive and HIV-negative patients were recorded in detail, and (3) the data came from the outbreak of mpox after 2022. The exclusion criteria were as follows: (1) nonhuman studies; (2) data duplication; (3) case reports, reviews or comments; (4) data before 2022; (5) suspected cases of mpox; and (6) experimental group or control group sample sizes of less than 5.

### 2.2 Search strategy and study selection

We searched for articles published in PubMed, Embase and Web of Science before July 2, 2025. To retrieve the available data to the maximum extent possible, we did not limit the language of the articles, and the retrieval scope included the title and abstract. The search strategy was as follows: ((((Human Immunodeficiency Virus[Title/Abstract]) OR (HIV[Title/Abstract])) OR (AIDS[Title/Abstract])) OR (Acquired Immune Deficiency Syndrome[Title/Abstract])) AND ((((monkeypox[Title/Abstract]) OR (mpox[Title/Abstract])) OR (MPXV[Title/Abstract])) OR (MPX[Title/Abstract])).

We first deleted duplicate articles by matching titles, authors and journals. We subsequently performed a preliminary screening of the articles by reading the title or abstract. The articles that passed the preliminary screening were further screened by reading the full text to determine which articles were eligible for meta-analysis.

### 2.3 Data selection and quality assessment

Data extraction was performed by three authors to ensure accuracy. Two of the authors screened the data independently, and disagreements were adjudicated by the third author. The selected items included age, race, sexually transmitted infection, use of tecovirimat, number of MSM, location of lesions, type of lesions, number of lesions, complications, symptoms, laboratory findings, days between first symptoms and clinical assessment, mpox vaccination, number of severe mpox cases, number of hospitalizations, hospitalization duration, and number of deaths.

The Newcastle–Ottawa quality assessment scale was used to evaluate the quality and bias risk of the included articles. An article with a score ≥7 indicated a high-quality article with a low risk of bias. If an article received a score of 0 or 1 in the outcome domain, it was classified as poor quality regardless of the total score.

### 2.4 Statistical analysis

Odds ratios (ORs) and standardized mean differences (SMDs) were used for data analysis and evaluation in which ORs were used for dichotomous variables, SMDs were used for continuous variables, and confidence intervals (CIs) were set at 95%. For data with only the sample size and quartile, we used the transformation formula to calculate the mean and standard deviation (Wan et al., 2014). The heterogeneity was quantified by *I*^2^ statistics, and the source of heterogeneity was determined via subgroup analysis: *I*^2^ ≤ 50% indicated low heterogeneity, 50% < *I*^2^ ≤ 75% indicated moderate heterogeneity, and *I*^2^ > 75% indicated high heterogeneity (Higgins et al., 2003). A random effects model was used to estimate the effect value and a sensitivity analysis was conducted to verify the robustness of the results. The statistical software used was Stata 14.0, and a *z* test *p* value<0.05 was considered to indicate statistical significance. Additionally, we employed Egger’s test to evaluate reporting bias, with a *p* value>0.05 indicating the absence of bias.

## 3 Results

### 3.1 Study selection and characteristics

**Figure 1:**
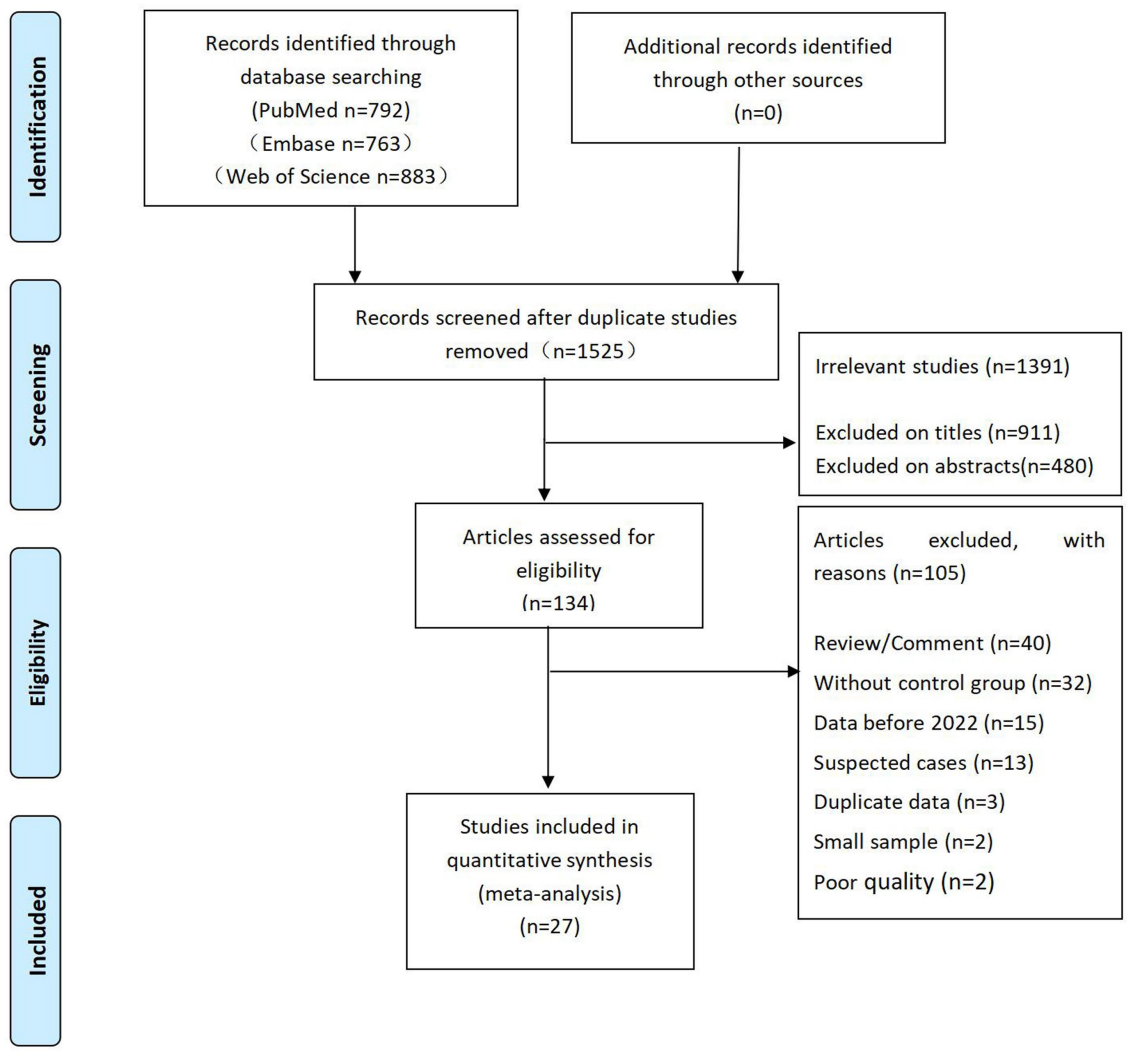
Flow diagram of the article selection process.

From the three databases, we retrieved a total of 2,438 articles and deleted 913 duplicate articles. A total of 1,391 articles that were not relevant to our study were excluded after screening the titles and abstracts. Of the remaining 134 articles, 107 were further excluded after screening the full text. The flow diagram of the article selection process is shown in Figure 1.

The article by Jin et al. (2025) included all the cases of mpox in China confirmed by the laboratory in 2023, but few items were studied. Among the other seven articles from China, six were from 2023 (Li et al., 2024; Zhao et al., 2024; Yang H. et al., 2024; Yan et al., 2024; Guo et al., 2024; Fu et al., 2023), and the other was from 2022 to 2024 (Hu et al., 2025). To avoid data duplication, the data in the other seven articles from China were not included in our meta-analysis if the items were listed in the article by Jin et al. For these articles, we included only the items that Jin et al. did not mention. We included two articles by Silva et al. (2022) and Silva et al. (2024), and the data of both articles came from the same institution. Because the sample size of the article published in 2024 was larger than that of the article published in 2022, we mainly included the data of the article in 2024. For the article published in 2022, we included only the items that were not mentioned in the 2024 article. Nunez et al. published two articles in 2024 (Nunez et al., 2024) and 2023 (Nunez et al., 2023). Since the 2024 article had a larger sample size and contained more items, we did not include the data from 2023. Yang et al. also published two articles in 2024 (Yang H. et al., 2024) and 2023 (Yang et al., 2023) with the aforementioned situation, and we included only the data from the 2024 article. The samples assessed by Li et al. (2024) and Zhou et al. (2024) were from the same institution during the same period. Since the study of Li et al. included more items, we did not include the article of Zhou et al.

The Newcastle–Ottawa quality assessment scale is described in Supplementary Table S1. We found that most of the included articles were of high quality and had a low risk of bias. In order to get rigorous results, we did not include the two studies with poor quality in the pooled estimates (Alpalhao et al., 2023; Agrati et al., 2023).

Our meta-analysis included 27 articles. Most studies had detected mpox virus via PCR, most HIV samples had reasonable CD4^+^ T-cell counts with low viral loads, and most patients received antiretroviral therapy. The details are listed in Table 1.

**Table 1:** Characteristics of individual studies.

Study	Country	Continent	Study period	Sample size	HIV-positive	HIV-negative	Diagnosis of mpox	Characteristics of HIV patients		
								CD4^+^ T-cell count (cells/mm^3^)	HIV viral load (copies/mL)	Number of patients receiving ART
Silva et al. (2022)	Brazil	South America	2022.6.12–2022.8.19	205	109	96	PCR	Median: 528	Undetectable: 99 patients	109
Li et al. (2024)	China	Asia	2023.6–2023.12	44	27	17	PCR	20 patients ≥ 200 (*24)	Undetectable: 16 patients (*24)	24
Aldred et al. (2024)	USA	North America	2022.6.1–2022.10.7	390	324	66	PCR	152 patients > 350 (*242)	138 patients<50 (*257)	NA
Zhao et al. (2024)	China	Asia	2023.7.1–2023.8.8	56	23	33	PCR	Median: 351 (*21)	Undetectable: 21 patients (*21)	21
Estevez et al. (2023)	Spain	Europe	2022.5.18–2022.9.30	99	71	28	PCR	Median: 253	Undetectable: 67 patients	71
Sousa et al. (2024)	Portugal	Europe	2022.1–2022.12	58	25	33	PCR	NA	The viral load of 22 patients remained stable	22
Caria et al. (2022)	Portugal	Europe	2022.5.5–2022.7.26	41	25	16	Nucleic Acid Amplification Testing	Median: 702	22 patients<50	25
Hoffmann et al. (2023)	Germany	Europe	2022.5.19–2022.6.30	546	256	290	PCR	Median: 691	226 patients<50 (*236)	NA
Yang H. et al. (2024)	China	Asia	2023.6.2–2023.6.10	41	21	20	PCR	Median: 825	NA	NA
Lim et al. (2024)	Korea	Asia	2022.6.1–2023.5.26	60	25	35	PCR	Median: 589	25 patients<20	25
Jin et al. (2025)	China	Asia	2023	1,702	802	900	NA	575 patients ≥ 200 (*652)	517 patients<50 (*606)	775
Angelo et al. (2023)	29 countries	Countries on six continents	2022.5.1–2022.7.1	226	92	134	PCR	Median: 713	Undetectable: 76 patients (*83)	NA
Curran et al. (2022)	USA	North America	2022.5.17–2022.7.22	1,969	755	1,214	NA	Median: 639	620 patients<200	710
Yan et al. (2024)	China	Asia	2023.6.15–2023.8.15	31	21	10	PCR	15 patients ≥ 200	NA	21
Silva et al. (2024)	Brazil	South America	2022.6.12–2022.12.31	409	213	196	PCR	Median: 624	180 patients<200	205
Corma-Gomez et al. (2024)	Spain	Europe	2022.4.27–2023.6.30	1,789	772	1,017	PCR	739 patients ≥ 350	745 patients<1,000	NA
Betancort-Plata et al. (2022)	Spain	Europe	2022.5.1–2022.7.31	42	27	15	PCR	Median: 759	25 patients<50	NA
Lozada et al. (2025)	Colombia	South America	2022	4,023	2,408	1,615	PCR	NA	NA	NA
Guo et al. (2024)	China	Asia	2023.6.2–2023.9.23	39	20	19	PCR	Median: 638	Undetectable: 18 patients	20
Nunez et al. (2024)	Mexico	North America	2022.5.24–2022.11.21	3,291	1,939	1,352	PCR	Median: 495 (*725)	NA	NA
Pilkington et al. (2023)	UK	Europe	2022.5–2022.12	144	58	86	PCR	Median: 508 (*22)	47 patients<200 (*52)	NA
Kowalski et al. (2023)	Poland	Europe	2022.5.16–2022.10.30	94	43	51	PCR	Median: 672	38 patients<50	42
Moraes-Cardoso et al. (2024)	Spain	Europe	2022.6.28–2022.9.22	33	14	19	NA	Median: 777	NA	11
Triana-Gonzalez et al. (2023)	Mexico	North America	2022.9–2022.12	72	64	8	PCR	Median: 506	Undetectable: 44 patients (*51)	51
Hu et al. (2025)	China	Asia	2022.9–2024.10	279	149	130	PCR	Median: 526	Most patients were detectable	140
Ramirez-Soto et al. (2024)	Peru	South America	2022.6.15–2023.12.31	3,561	2,123	1,438	PCR	NA	NA	1,796
Fu et al. (2023)	China	Asia	2023.6.1–2023.7.31	115	65	50	Positive mpox virus nucleic acid test or mpox virus culture isolated from any anatomical site	39 patients ≥ 500	Undetectable: 56 patients (*60)	60

*The number of patients with test results.

ART, antiretroviral therapy; HIV, human immunodeficiency virus; PCR, polymerase chain reaction.

### 3.2 Results of syntheses

#### 3.2.1 Demographics, coinfections and complications

**Figure 2:**
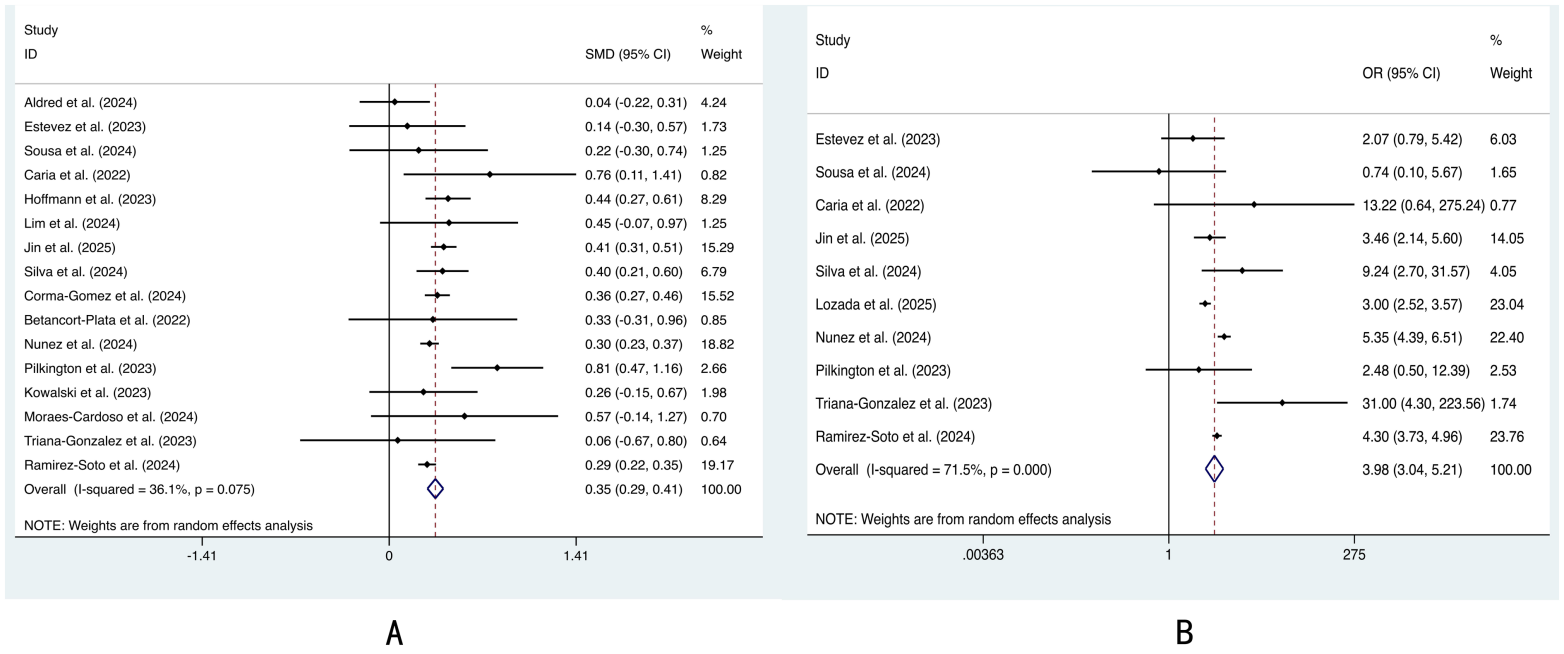
Forest plot of differences in the clinical characteristics between the HIV-positive group and the HIV-negative group: **(A)** age and **(B)** men who have sex with men.

Silva et al. noted that most published studies on mpox cases after 2022 included at least 99% cisgender men (Silva et al., 2022). In our meta-analysis, nearly half of the studies included samples consisting solely of males. Therefore, we did not compare sex differences between HIV-positive patients and HIV-negative patients. We found that the age of HIV-positive patients was significantly greater than that of HIV-negative patients (SMD = 0.35, 95% CI: 0.29–0.41; Figure 2), and that there were more MSM among the HIV-positive patients (OR = 3.98, 95% CI: 3.04–5.21; Figure 2). Although black people showed more trends and Asian people showed fewer trends in HIV-positive patients, the racial difference was not significant (Supplementary Figures S1–S3).

There was no significant difference between the two groups of patients in terms of gonorrhea or herpes (Supplementary Figures S4, S5), but HIV-positive patients had more syphilis (OR = 2.66, 95% CI: 2.13–3.33; Supplementary Figure S6), hepatitis B (OR = 3.94, 95% CI: 2.93–5.30; Supplementary Figure S7), hepatitis C (OR = 5.71, 95% CI: 3.06–10.64; Supplementary Figure S8) and chlamydia (OR = 1.35, 95% CI: 1.11–1.64; Supplementary Figure S9). In terms of complications, HIV-positive patients had more proctitis (OR = 1.52, 95% CI: 1.17–1.98; Supplementary Figure S10), while the proportions of patients with bacterial infection, urethritis, tonsillitis and pneumonia were similar to those of HIV-negative patients (Supplementary Figures S11–S14).

#### 3.2.2 Symptoms and laboratory findings

Among the 11 symptoms related to mpox that we studied, only fever (OR = 1.15, 95% CI: 1.01–1.30; Supplementary Figure S15) and diarrhea (OR = 1.69, 95% CI: 1.03–2.77; Supplementary Figure S16) were more common in HIV-positive patients, and the other nine symptoms, including rectal pain, lymphadenopathy, headache, sore throat, asthenia, myalgia, nausea, arthralgia and vomiting, were not significantly different between the two groups of patients (Supplementary Figures S17–S25).

We also found that HIV infection seemed to be associated with a decrease in hemoglobin (SMD = −0.43, 95% CI: −0.64 to −0.22; Supplementary Figure S26) and albumin (SMD = −0.35, 95% CI: −0.55 to −0.15; Supplementary Figure S27) levels in mpox patients, while it had no significant effect on white blood cell count, neutrophil count, lymphocyte count, monocyte count, platelet count, alanine transaminase, total bilirubin, lactate dehydrogenase, creatinine, creatine kinase, procalcitonin or C-reactive protein (Supplementary Figures S28–S39). Among the T-cell subgroups, the CD4^+^ T-cell count of HIV-positive patients was significantly lower than that of HIV-negative patients (SMD = −0.78, 95% CI: −1.34 to −0.23; Supplementary Figure S40), while the CD8^+^ T-cell count yielded the opposite statistical conclusion (SMD = 0.35, 95% CI: 0.03–0.67; Supplementary Figure S41). However, the results were based on data from only three studies, which might affect the generalizability and robustness of the conclusions. Therefore, avoiding overinterpreting its clinical mechanism or significance is necessary.

#### 3.2.3 Rash characteristics

We performed a detailed analysis of the rash of mpox patients, and there was no significant difference in the number of rashes (>10) between the two groups (Supplementary Figure S42). The lesions of HIV-positive patients were more frequently distributed on the neck (OR = 1.23, 95% CI: 1.05–1.45; Supplementary Figure S43), trunk (OR = 1.36, 95% CI: 1.04–1.79; Supplementary Figure S44) and anus (OR = 2.12, 95% CI: 1.08–4.16; Supplementary Figure S45), whereas the distribution of lesions on the palms, soles, face/head, arms, legs, oral cavity and genitals were similar for both groups of patients (Supplementary Figures S46–S52). We compared different types of lesions and found that maculae were less common in HIV-positive patients (OR = 0.87, 95% CI: 0.76–0.99; Supplementary Figure S53), while pustules were more common (OR = 1.33, 95% CI: 1.08–1.62; Supplementary Figure S54). There were no significant differences in terms of the number of papulae, vesicles or ulcerations (Supplementary Figures S55–S57).

#### 3.2.4 Treatment and clinical outcomes

Our study revealed that infection with HIV had no association with a delay in the days between first symptoms and clinical assessment for mpox patients (Supplementary Figure S58). There was also no significant difference in the number of patients who received the mpox vaccine between the two groups (Supplementary Figure S59). Although HIV coinfection did not increase the hospitalization duration of mpox patients (Supplementary Figure S60), more HIV-positive patients required hospitalization (OR = 1.63, 95% CI: 1.22–2.19; Supplementary Figure S61), and more HIV-positive patients received tecovirimat treatment (OR = 4.25, 95% CI: 1.59–11.4; Supplementary Figure S62). We also found that HIV-positive patients were more likely to progress to severe mpox (OR = 1.82, 95% CI: 1.28–2.58; Supplementary Figure S63), and that the mortality was greater than that of HIV-negative patients (OR = 3.36, 95% CI: 1.15–9.83; Supplementary Figure S64). The details are listed in Table 2.

**Table 2:** Comparison of clinical characteristics between the HIV-positive group and the HIV-negative group.

Items	Number of articles	Sample size of the HIV-positive group	Sample size of the HIV-negative group	OR	OR^#^	SMD	SMD^#^	95% CI	95% CI^#^	*I* ^2^	*p*	*p* ^#^
Age	16	6,781	5,550			0.35	0.33	0.29–0.41	0.30–0.37	36.1%	**<0.001**	**<0.001**
Men who have sex with men	10	7,728	5,672	3.98	4.04			3.04–5.21	3.68–4.43	71.5%	**<0.001**	**<0.001**
Race												
Black	3	1,292	1,476	2.52	2.76			0.90–7.07	2.27–3.35	93.3%	0.078	**<0.001**
White	4	1,350	1,562	0.70	0.51			0.29–1.73	0.43–0.60	92.4%	0.440	**<0.001**
Asian	2	1,079	1,280	0.31	0.43			0.08–1.19	0.26–0.71	40.2%	0.088	**0.001**
Sexually transmitted infection												
Syphilis	12	8,039	6,152	2.66	2.60			2.13–3.33	2.29–2.95	49.6%	**<0.001**	**<0.001**
Gonorrhea	7	3,315	2,893	1.64	1.48			0.97–2.78	1.23–1.77	54.1%	0.067	**<0.001**
Hepatitis B	9	3,502	2,391	3.94	4.05			2.93–5.30	3.02–5.44	0	**<0.001**	**<0.001**
Hepatitis C	9	5,421	3,724	5.71	7.46			3.06–10.64	5.30–10.51	54.0%	**<0.001**	**<0.001**
Chlamydia	8	3,257	2,786	1.35	1.36			1.11–1.64	1.12–1.64	0	**0.020**	**0.002**
Herpes	5	2,503	1,571	1.70	2.15			0.72–4.00	0.94–4.90	0	0.223	0.069
Complications												
Proctitis	9	6,019	5,167	1.52	1.54			1.17–1.98	1.28–1.84	30.0%	**0.002**	**<0.001**
Bacterial infection	7	621	361	0.92	0.96			0.56–1.51	0.60–1.55	0%	0.736	0.865
Urethritis	4	2,097	1,421	1.56	1.69			0.56–4.30	0.68–4.21	7.9%	0.392	0.258
Tonsillitis	2	172	163	1.96	1.95			0.52–7.44	0.51–7.45	0	0.324	0.328
Pneumonia	3	108	61	1.41	2.21			0.06–34.96	0.63–7.75	73.7%	0.833	0.216
Symptoms												
Rectal pain	3	481	250	1.50	1.51			0.89–2.52	0.91–2.51	0	0.127	0.110
Fever	16	8,943	7,214	1.15	1.11			1.01–1.30	1.03–1.18	49.1%	**0.030**	**0.003**
Lymphadenopathy	12	9,407	7,896	1.21	1.30			0.78–1.87	1.21–1.38	96.7%	0.388	**<0.001**
Headache	13	8,083	5,823	0.94	0.93			0.82–1.08	0.86–0.99	41.6%	0.400	**0.049**
Sore throat	12	5,962	4,367	1.00	1.00			0.88–1.15	0.91–1.10	14.4%	0.982	0.944
Asthenia	11	6,833	5,369	1.07	1.07			0.95–1.21	0.98–1.16	23.5%	0.260	0.130
Myalgia	10	7,670	5,716	1.06	1.05			0.95–1.18	0.97–1.13	24.4%	0.305	0.261
Diarrhea	5	2,985	2,533	1.69	1.73			1.03–2.77	1.19–2.51	29.2%	**0.039**	**0.004**
Nausea	3	2,140	1,582	1.06	1.06			0.84–1.32	0.85–1.33	0	0.636	0.615
Arthralgia	4	2,144	1,509	1.08	1.08			0.94–1.23	0.94–1.24	0	0.301	0.260
Vomiting	2	2,048	1,448	1.00	1.01			0.69–1.45	0.70–1.47	0	0.986	0.943
Laboratory findings												
White blood cell count	9	444	332			−0.06	−0.06	−0.21–0.09	−0.21–0.09	0	0.420	0.420
Neutrophil count	6	353	213			0.03	−0.05	−0.23–0.29	−0.23–0.13	36.7%	0.799	0.567
Lymphocyte count	7	376	246			−0.06	−0.06	−0.22–0.11	−0.23–0.11	0	0.505	0.505
Monocyte count	3	197	167			0.84	0.13	−0.48–2.16	−0.08–0.34	94.6%	0.215	0.222
Platelet count	7	380	261			−0.01	−0.05	−0.27–0.26	−0.21–0.12	48.4%	0.954	0.579
Hemoglobin	6	357	228			−0.43	−0.42	−0.64– −0.22	−0.59– −0.24	16.2%	**<0.001**	**<0.001**
Albumin	4	220	190			−0.35	−0.35	−0.55– −0.15	−0.55– −0.15	0	**<0.001**	**<0.001**
Alanine transaminase	4	238	226			−0.03	−0.03	−0.21–0.16	−0.21–0.16	0	0.778	0.778
Total bilirubin	4	272	210			−0.33	−0.14	−0.79–0.14	−0.32–0.05	78.1%	0.169	0.152
Lactate dehydrogenase	4	142	88			0.15	0.27	−0.77–1.07	−0.02–0.55	89.8%	0.747	0.067
Creatinine	6	357	228			0.23	0.21	−0.06–0.52	0.03–0.38	51.4%	0.122	**0.019**
Creatine kinase	2	50	50			−0.23	−0.23	−0.64–0.18	−0.64–0.18	0	0.266	0.266
Procalcitonin	4	261	165			−0.11	0.17	−0.62–0.39	−0.04–0.37	69.7%	0.656	0.104
C-reactive protein	7	402	302			0.25	0.27	−0.02–0.53	0.11–0.43	59.6%	0.071	**0.001**
CD4^+^ T-cell count	3	193	183			−0.78	−0.77	−1.34– −0.23	−0.98– −0.56	74.3%	**0.005**	**<0.001**
CD8^+^ T-cell count	3	193	183			0.35	0.36	0.03–0.67	0.15–0.57	36.7%	**0.035**	**0.001**
Number of lesions (>10)	5	389	357	1.01	1.03			0.48–2.12	0.71–1.50	54.3%	0.982	0.878
Location of lesions												
Palms	3	2,102	1,514	1.08	1.08			0.92–1.27	0.93–1.27	0	0.333	0.320
Soles	3	2,102	1,514	1.03	1.03			0.85–1.24	0.85–1.24	0	0.786	0.777
Neck	4	2,167	1,564	1.23	1.24			1.05–1.45	1.06–1.45	0	**0.009**	**0.008**
Face/Head	3	410	126	1.11	1.17			0.72–1.11	0.73–1.71	0	0.629	0.617
Arms	3	2,102	1,514	3.13	1.24			0.77–12.82	1.07–1.43	89.6%	0.112	**0.003**
Legs	2	2,010	1,380	1.38	1.03			0.57–3.36	0.90–1.18	62.0%	0.478	0.665
Trunk	6	678	405	1.36	1.36			1.04–1.79	1.04–1.79	0	**0.027**	**0.026**
Oral cavity	3	916	979	0.87	0.97			0.31–2.45	0.61–1.56	66.1%	0.789	0.905
Genitals	11	3,478	2,464	0.79	0.97			0.60–1.06	0.87–1.08	66.3%	0.110	0.566
Anus	4	479	465	2.12	1.61			1.08–4.16	1.22–2.13	67.1%	**0.029**	**0.001**
Type of lesions												
Maculae	5	2,463	1,504	0.87	0.87			0.76–0.99	0.76–0.99	0	**0.041**	**0.043**
Papulae	5	2,463	1,504	0.95	0.97			0.72–1.24	0.85–1.11	22.8%	0.695	0.676
Vesicle	5	2,463	1,504	1.05	1.05			0.92–1.20	0.92–1.20	0	0.443	0.442
Pustules	5	2,463	1,504	1.33	1.35			1.08–1.62	1.17–1.55	7.4%	**0.006**	**<0.001**
Ulceration	6	698	298	1.25	0.99			0.50–3.14	0.68–1.46	72.0%	0.638	0.976
Days between first symptoms and clinical assessment	5	619	348			0.03	0.03	−0.11–0.17	−0.11–0.17	0	0.632	0.632
Mpox vaccination	9	1,877	1,892	1.23	0.97			0.67–2.26	0.76–1.23	71.8%	0.499	0.800
Hospitalizations	13	9,079	7,503	1.63	1.80			1.22–2.19	1.56–2.08	63.3%	**0.001**	**<0.001**
Hospitalization duration	3	821	1,298			0.01	−0.03	−0.58–0.59	−0.33–0.27	73.5%	0.984	0.849
Use of tecovirimat	3	441	235	4.25	5.38			1.59–11.40	2.73–10.61	49.1%	**0.004**	**<0.001**
Severe mpox	4	2,380	1,587	1.82	1.86			1.28–2.58	1.32–2.62	0	**0.001**	**<0.001**
Mortality	7	7,220	4,805	3.36	5.44			1.15–9.83	2.37–12.51	0	**0.027**	**<0.001**

^#^Fixed effects model.

HIV, human immunodeficiency virus.Bold values, significant difference.

### 3.3 Reporting biases, heterogeneity and sensitivity analysis

Egger’s test was used for reporting bias analysis, and the results revealed that most of our findings had a low risk of bias (Figure 3 and Supplementary Figures S65–S121). Notably, the statistical efficiency of Egger’s test is low when the number of included studies is small (<10), and fully identifying publication bias may not be possible; thus, these results should be interpreted with greater caution.

**Figure 3:**
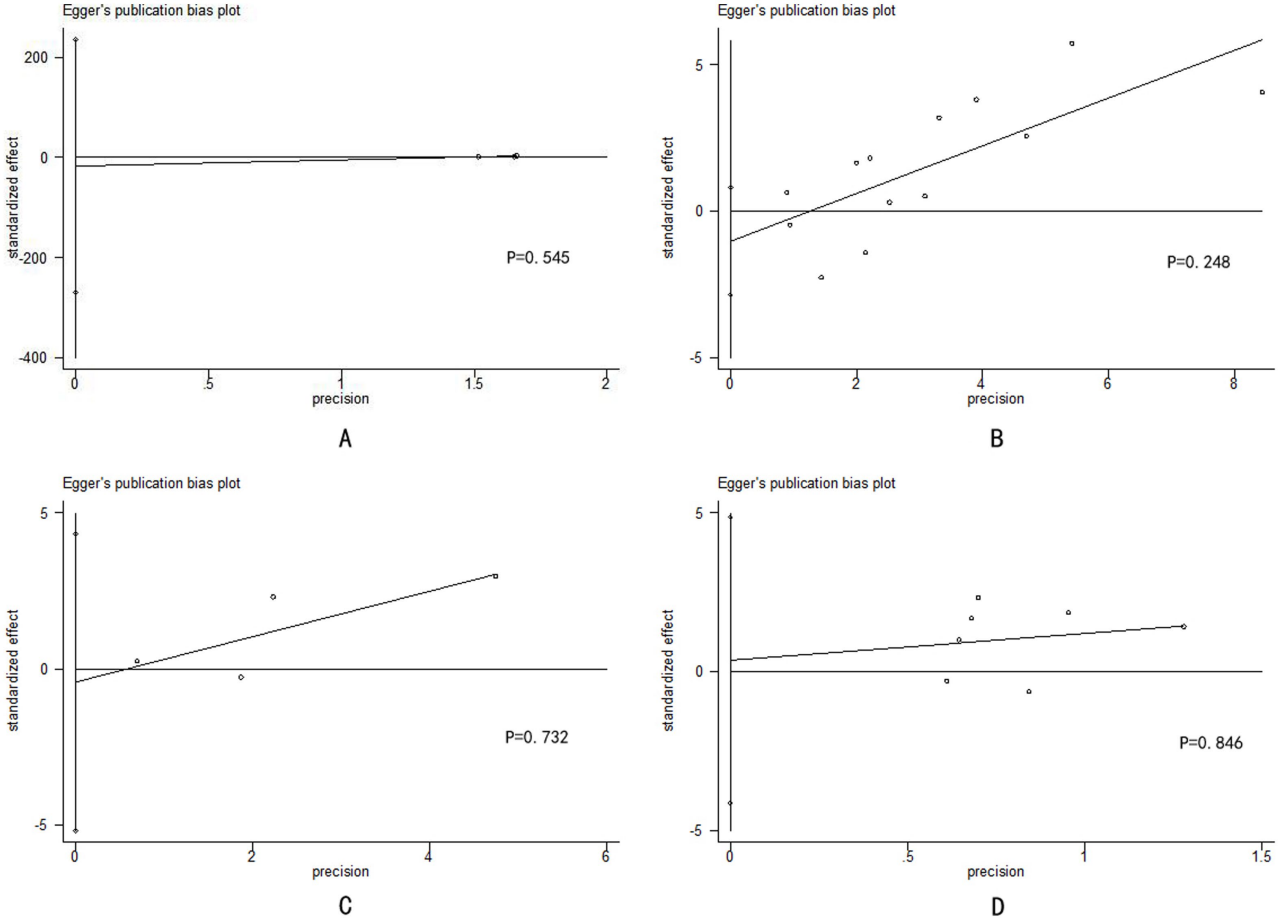
Egger’s publication bias plot of differences in the clinical characteristics between the HIV-positive group and the HIV-negative group: **(A)** use of tecovirimat; **(B)** hospitalization; **(C)** severe mpox; and **(D)** mortality.

Most of our results showed low or moderate heterogeneity. Among the seven results with high heterogeneity, six were not suitable for subgroup analysis (Supplementary Figures S1, S2, S31, S34, S35, S49) because of the small number of included studies (<5). We tried to explain the result in Supplementary Figure S18 through subgroup analysis; unfortunately, we did not find the exact source of heterogeneity.

All results were recalculated using a fixed effects model and compared with those from the random effects model. The results from the fixed-effects model are presented in Table 2. Most results (57/66) were consistent across the two models. Among the nine results with significant changes, seven had *I*^2^ statistics greater than 50%, and all nine changed from statistically insignificant to significant, and the CIs narrowed. The greater the heterogeneity, the more likely that the fixed effects model ignores the differences between studies, thus yielding a significant result of “false-positive.” These results support the rationality of our initial choice of the random effects model.

## 4 Discussion

Since the outbreak of the mpox epidemic in 2022, coinfection with HIV has received significant attention from health care workers. However, in terms of evidence-based medicine, several limitations still exist (Shin et al., 2023; Taha et al., 2024; Shabil et al., 2025). Shin et al. published a systematic review and meta-analysis in 2023 (Shin et al., 2023), which made outstanding contributions to the research in this field. However, among the included articles, case series and reports accounted for 87.87%, which might have led to the original data having low evidence levels, a high risk of bias, and difficulties in evaluating the universality of the results. Nunez et al. (2024) also reported that Shin et al. (2023) study included mainly case reports and small series, and that this uneven reporting limits the conclusions that may be drawn. This limitation may be because their study started at the early stage of the epidemic, and it was difficult to collect control data. Taha et al. (2024) and Shabil et al. (2025) also conducted detailed studies on mpox patients with HIV, but they focused on fewer items. Shabil et al. (2025) only analyzed hospitalization throughout the entire article. Our data collection was conducted in 2025. The sample size was larger and more up to date. All included articles could extract control data, and small sample studies were excluded. Therefore, the data used for the meta-analysis were more rigorous, the evidence level and methodological quality were higher, the statistical power was stronger, and the causal inference ability was better. Additionally, we compared differences in more than 60 clinical characteristics between HIV-positive patients and HIV-negative patients, which is helpful for providing a reference for clinical decisions.

Our study revealed that HIV coinfection had varying degrees of impact on the location of lesions, lesion type, complications, symptoms, laboratory findings, and final clinical outcomes of mpox patients during the current epidemic.

A previous study has suggested that HIV infection might play a part in the function or senescence of B and T immune compartments and could lead to a reduced level or persistence of protective response to natural infection, as reported in other viral diseases (Spinelli et al., 2021). It has been reported that specific mpox virus and orthopoxvirus T-cell-mediated responses were well coordinated with the mpox virus antibody concentration in people without HIV, but not in those with HIV. Moreover, compared with individuals without HIV, NK cell levels were reduced in people with HIV during both the acute and recovery phases, which could indicate that the dysfunction of NK cells induced by HIV may weaken protective immunity against the mpox virus (Liu et al., 2025). Guo et al. found that mpox patients with HIV had higher concentrations of MIP-1α, MIP-1β, G-CSF, and FGF basic, but lower neutralizing antibody titers than those without HIV, indicating that HIV-driven immune suppression might impair antibody responses (Guo et al., 2024). Additionally, HIV coinfection has been proposed to affect mpox virus gene expression, with notable changes in virus’s immune evasion strategies, and corresponding changes in the host immune response. Several immune-related genes were dysregulated between early and late stages of mpox in lesions from individuals coinfected with HIV, potentially impairing antiviral defenses and lymphocyte recruitment, a pattern absent in lesions from patients with only mpox infection (Hurst et al., 2025).

Our study revealed that among mpox patients, those infected with HIV were older, which might be related to the significant increase in HIV prevalence among elderly individuals in recent years (Vance et al., 2011). Tecovirimat is a tetracyclic acyl hydrazide targeting the VP37 protein that was developed specifically for poxviruses and has antiviral activity against other orthopoxviruses, including the mpox virus (Grosenbach et al., 2018). In animal models, tecovirimat has been shown to prevent morbidity and mortality associated with mpox (Russo et al., 2018). We found that HIV-positive patients required more hospitalizations and tecovirimat treatment, further indicating that HIV coinfection may aggravate the condition of mpox patients and require more medical resources.

Notably, caution should be exercised when interpreting the association between HIV coinfection and clinical outcomes. Specifically, the CIs for some results (such as mortality and tecovirimat use) were notably wide, reflecting statistical imprecision due to the limited number of events; thus, there is uncertainty in the estimated effects of these variables. Furthermore, outcomes such as hospitalization and severe mpox may be influenced by unmeasured confounders, including lower admission thresholds for immunocompromised patients or differences in health care access, rather than biological severity alone.

An overview of HIV epidemiology among MSM around the world highlighted that the risk of HIV infection among MSM was 10–20 times higher than that of the general population, and the infection rate was significantly higher (Beyrer et al., 2012). This disparity might explain the overrepresentation of MSM among HIV-positive patients in our study, suggesting that MSM status might be a critical confounder. HIV coinfection in patients (especially those who have not been treated or diagnosed) may increase the risk of simultaneous exposure to other sexually transmitted diseases due to increased sexual activity, multiple sexual partners or unprotected sex, leading to the co-transmission of multiple pathogens. A report from the United States showed that the incidence of syphilis in HIV-positive people was 3–5 times greater than that in HIV-negative people, especially in the MSM population, and that the syphilis infection rate of HIV-positive patients could be as high as 25% (Salado-Rasmussen et al., 2015). We found that proctitis was more common among HIV-positive patients, which might be associated with higher rates of anal sex behaviors among the MSM community. Moreover, having more sexually transmitted diseases may further aggravate rectal inflammation. One point that needs to be emphasized is that mpox can infect individuals of any sexual orientation. People must avoid stigma and discrimination against the MSM community. The WHO has warned that stigma toward specific groups may hinder patients from seeking timely medical treatment, undermine community trust, and ultimately weaken the effectiveness of epidemic prevention (World Health Organization, 2022b). Public health information transmission and intervention measures should not only effectively identify and cover high-risk groups but also balance ethical principles that promote social inclusion and ensure health equity.

Our study had several limitations: (1) The overall heterogeneity of our results was minimal, but there were still a few results with high heterogeneity. Due to the small number of included articles (<5), we did not conduct subgroup analysis and could not determine the exact source of the heterogeneity. (2) Although we found that HIV-positive patients were more likely to progress to severe mpox, due to the lack of globally recognized standards, determining the severity of mpox was still a challenge. We could only determine the number of severe cases in each included article based on the descriptions in the original data. (3) Most HIV-positive patients included in this meta-analysis had well-controlled infections with preserved CD4^+^ T-cell counts. Therefore, the findings may not be generalizable to patients with advanced or untreated HIV infection. Few published articles have focused on mpox patients with severe HIV, so we are unable to fully assess the potential detrimental effects of uncontrolled HIV viremia or associated immunodeficiency on mpox presentation. We will continue to pay attention to this field in future studies.

## 5 Conclusion

Overall, HIV coinfection may influence the disease process and clinical indicators of mpox patients and is associated with more severe clinical outcomes. Our study provides a reference for the clinical diagnosis and treatment of patients and the public health management of mpox, suggesting the need to strengthen the comprehensive management of mpox patients with HIV, with a particular focus on screening and treating concurrent sexually transmitted infections.

## Data Availability

The original contributions presented in the study are included in the article/Supplementary material, further inquiries can be directed to the corresponding author.

## References

[ref1] Agrati C. Cossarizza A. Mazzotta V. Grassi G. Casetti R. Biasi S. D. . (2023). Immunological signature in human cases of monkeypox infection in 2022 outbreak: an observational study. Lancet Infect. Dis. 23, 320–330. doi: 10.1016/S1473-3099(22)00662-4, 36356606 PMC9761565

[ref2] Aldred B. Scott J. Y. Aldredge A. Gromer D. J. Anderson A. M. Cartwright E. J. . (2024). Associations between HIV and severe Mpox in an Atlanta cohort. J. Infect. Dis. 229, S234–S242. doi: 10.1093/infdis/jiad505, 38001044

[ref3] Alpalhao M. Sousa D. Frade J. V. Patrocinio J. Garrido P. M. Correia C. . (2023). Human immunodeficiency virus infection may be a contributing factor to monkeypox infection: analysis of a 42-case series. J. Am. Acad. Dermatol. 88, 720–722. doi: 10.1016/j.jaad.2022.09.029, 36156305 PMC9534227

[ref4] Angelo K. M. Smith T. Camprubí-Ferrer D. Balerdi-Sarasola L. Menéndez M. D. Servera-Negre G. . (2023). Epidemiological and clinical characteristics of patients with monkeypox in the GeoSentinel network: a cross-sectional study. Lancet Infect. Dis. 23, 196–206. doi: 10.1016/S1473-3099(22)00651-X, 36216018 PMC9546520

[ref5] Betancort-Plata C. Lopez-Delgado L. Jaen-Sanchez N. Tosco-Nunez T. Suarez-Hormiga L. Lavilla-Salgado C. . (2022). Monkeypox and HIV in the Canary Islands: a different pattern in a mobile population. Trop. Med. Infect. Dis. 7:318. doi: 10.3390/tropicalmed7100318, 36288059 PMC9608065

[ref6] Beyrer C. Baral S. D. Griensven F. V. Goodreau S. M. Chariyalertsak S. Wirtz A. L. . (2012). Global epidemiology of HIV infection in men who have sex with men. Lancet 380, 367–377. doi: 10.1016/S0140-6736(12)60821-6, 22819660 PMC3805037

[ref7] Caria J. Pinto R. Leal E. Almeida V. Cristovao G. Goncalves A. C. . (2022). Clinical and epidemiological features of hospitalized and ambulatory patients with human monkeypox infection: a retrospective observational study in Portugal. Infect. Dis. Rep. 14, 810–823. doi: 10.3390/idr14060083, 36412741 PMC9680313

[ref8] Corma-Gomez A. Cabello A. Orviz E. Morante-Ruiz M. Ayerdi O. Al-Hayani A. . (2024). Long or complicated mpox in patients with uncontrolled HIV infection. J. Med. Virol. 96:e29511. doi: 10.1002/jmv.29511, 38469884

[ref9] Curran K. G. Eberly K. Russell O. O. Snyder R. E. Phillips E. K. Tang E. C. . (2022). HIV and sexually transmitted infections among persons with Monkeypox - eight U.S. jurisdictions, may 17-July 22, 2022. MMWR Morb. Mortal Wkly. Rep. 71, 1141–1147. doi: 10.15585/mmwr.mm7136a1, 36074735 PMC9470220

[ref10] Estevez S. Vara M. Gamo M. Manzano S. Troya J. Botezat E. . (2023). Epidemiological and clinical characteristics of patients admitted to a secondary hospital with suspected MPOX virus infection: is HIV playing a role? J. Clin. Med. 12:4124. doi: 10.3390/jcm12124124, 37373818 PMC10299369

[ref11] Fu L. Wang B. Wu K. Yang L. Hong Z. Wang Z. . (2023). Epidemiological characteristics, clinical manifestations, and mental health status of human mpox cases: a multicenter cross-sectional study in China. J. Med. Virol. 95:e29198. doi: 10.1002/jmv.29198, 37881113

[ref12] Girometti N. Byrne R. Bracchi M. Heskin J. Mcowan A. Tittle V. . (2022). Demographic and clinical characteristics of confirmed human monkeypox virus cases in individuals attending a sexual health centre in London, UK: an observational analysis. Lancet Infect. Dis. 22, 1321–1328. doi: 10.1016/S1473-3099(22)00411-X, 35785793 PMC9534773

[ref13] Grosenbach D. W. Honeychurch K. Rose E. A. Chinsangaram J. Frimm A. Maiti B. . (2018). Oral Tecovirimat for the treatment of smallpox. N. Engl. J. Med. 379, 44–53. doi: 10.1056/NEJMoa1705688, 29972742 PMC6086581

[ref14] Guo L. Song R. Zhang Q. Li D. Chen L. Fang M. . (2024). Profiling of viral load, antibody and inflammatory response of people with monkeypox during hospitalization: a prospective longitudinal cohort study in China. EBioMedicine 106:105254. doi: 10.1016/j.ebiom.2024.105254, 39043012 PMC11318531

[ref15] Higgins J. P. Thompson S. G. Deeks J. J. Altman D. G. (2003). Measuring inconsistency in meta-analyses. BMJ 327, 557–560. doi: 10.1136/bmj.327.7414.557, 12958120 PMC192859

[ref16] Hoffmann C. Jessen H. Wyen C. Grunwald S. Noe S. Teichmann J. . (2023). Clinical characteristics of monkeypox virus infections among men with and without HIV: a large outbreak cohort in Germany. HIV Med. 24, 389–397. doi: 10.1111/hiv.13378, 36059149

[ref17] Hu H. Zheng Y. Ruan L. Liu Y. Tong Y. Chen Y. . (2025). Clinical, epidemiological, virological characteristics and outcomes of 286 patients infected with monkeypox virus in China. Allergy 80, 1436–1451. doi: 10.1111/all.16540, 40156486 PMC12105066

[ref18] Hurst J. R. Tan D. H. S. Li A. Khera S. S. Persaud R. Chan A. . (2025). Monkeypox virus transcriptional profiles and host responses in skin lesion swabs among individuals with HIV. J. Infect. Dis. 2025:jiaf316. doi: 10.1093/infdis/jiaf316 PMC12614975 40498594

[ref19] Jin Y. Qin Q. Li C. Tang H. Zhang D. Bai W. . (2025). Comparative analysis of epidemiological and clinical characteristics between mpox cases with and without HIV-China, 2023. China CDC Wkly. 7, 233–238. doi: 10.46234/ccdcw2025.037, 39974765 PMC11832442

[ref20] Kowalski J. Cielniak I. Garbacz-Lagozna E. Cholewińska-Szymańska G. Parczewski M. (2023). Comparison of clinical course of Mpox among HIV-negative and HIV-positive patients: a 2022 cohort of hospitalized patients in Central Europe. J. Med. Virol. 95:e29172. doi: 10.1002/jmv.29172, 37861345

[ref21] Ladnyj I. D. Ziegler P. Kima E. (1972). A human infection caused by monkeypox virus in Basankusu territory, Democratic Republic of the Congo. Bull. World Health Organ. 46, 593–597, 4340218 PMC2480792

[ref22] Li J. Yuan X. Peng J. Hou X. Zheng F. Xiao G. . (2024). An epidemiological and clinical study of monkeypox in Changsha, China: a retrospective analysis of HIV-infected and non-HIV-infected patients from June to December 2023. Infect Drug Resist. 17, 5305–5313. doi: 10.2147/IDR.S485232, 39628830 PMC11614583

[ref23] Lim S. Y. Jo H. J. Lee S. Y. Ahn M. Kim Y. Jeon J. . (2024). Clinical features of mpox patients in Korea: a multicenter retrospective study. J. Korean Med. Sci. 39:e19. doi: 10.3346/jkms 38288533 PMC10825456

[ref24] Liu Y. Liu X. Wang J. Xie Y. Guo J. Liu Z. . (2025). Single-cell sequencing of peripheral blood mononuclear cells reveals immune landscape of monkeypox patients with HIV. Emerg. Microbes Infect. 14:2459136. doi: 10.1080/22221751.2025.2459136, 39868995 PMC11809181

[ref25] Lozada C. M. M. Reales J. A. M. Narvaez P. E. C. Alvarado F. E. P. (2025). Prevalence of HIV history and associated factors in people affected by mpox in Colombia, 2022. medRxiv. doi: 10.1101/2025.02.05.25321718

[ref26] Martinez J. I. Montalbán E. G. Bueno S. J. Martínez F. M. Juliá A. N. Díaz J. S. . (2022). Monkeypox outbreak predominantly affecting men who have sex with men, Madrid, Spain, 26 April to 16 June 2022. Euro Surveill. 27:2200471. doi: 10.2807/1560-7917.ES.2022.27.27.2200471, 35801519 PMC9264731

[ref27] Moraes-Cardoso I. Benet S. Carabelli J. Perez-Zsolt D. Mendoza A. Rivero A. . (2024). Immune responses associated with mpox viral clearance in men with and without HIV in Spain: a multisite, observational, prospective cohort study. Lancet Microbe. 5:100859. doi: 10.1016/S2666-5247(24)00074-0, 38857615

[ref28] Nunez I. Ceballos-Liceaga S. E. Torre A. García-Rodríguez G. López-Martínez I. López-Gatell H. . (2024). Clinical features and outcomes of mpox in people with and without HIV: a national comparative study. J. Acquir. Immune Defic. Syndr. 96, 166–170. doi: 10.1097/QAI.0000000000003407, 38465911

[ref29] Nunez I. García-Grimshaw M. Ceballos-Liceaga S. E. Toledo-Salinas C. Carbajal-Sandoval G. Sosa-Laso L. . (2023). Epidemiological and clinical characteristics of patients with human monkeypox infection in Mexico: a nationwide observational study. Lancet Reg. Health Americas 17:100392. doi: 10.1016/j.lana.2022.100392, 36405887 PMC9646405

[ref30] Ogoina D. Iroezindu M. James H. L. Oladokun R. Yinka-Ogunleye A. Wakama P. . (2020). Clinical course and outcome of human monkeypox in Nigeria. Clin. Infect. Dis. 71, e210–e214. doi: 10.1093/cid/ciaa143 32052029

[ref31] Parker S. Nuara A. Buller R. M. L. Schultz D. A. (2007). Human monkeypox: an emerging zoonotic disease. Future Microbiol. 2, 17–34. doi: 10.2217/17460913.2.1.17, 17661673

[ref32] Peiro-Mestres A. Fuertes I. Camprubí-Ferrer D. Ángeles M. M. Vilella A. Navarro M. . (2022). Frequent detection of monkeypox virus DNA in saliva, semen, and other clinical samples from 12 patients, Barcelona, Spain, May to June 2022. Euro Surveill. 27:2200503. doi: 10.2807/1560-7917.ES.2022.27.28.2200503 35837964 PMC9284919

[ref33] Pilkington V. Quinn K. Campbell L. Payne L. Brady M. Post F. A. (2023). Clinical presentation of mpox in people with and without HIV in the United Kingdom during the 2022 global outbreak. AIDS Res. Hum. Retrovir. 39, 581–586. doi: 10.1089/AID.2023.0014, 37071153

[ref34] Ramirez-Soto M. C. Arroyo-Hernández H. (2024). Epidemiological and clinical characteristics of monkeypox among people with and without HIV in Peru: a national observational study. J. Infect. Public Health 17:102494. doi: 10.1016/j.jiph.2024.102494, 39024895

[ref35] Russo A. T. Grosenbach D. W. Brasel T. L. Baker R. O. Cawthon A. G. Reynolds E. . (2018). Effects of treatment delay on efficacy of tecovirimat following lethal aerosol monkeypox virus challenge in cynomolgus macaques. J. Infect. Dis. 218, 1490–1499. doi: 10.1093/infdis/jiy326, 29982575 PMC6151088

[ref36] Salado-Rasmussen K. (2015). Syphilis and HIV co-infection. Epidemiology, treatment and molecular typing of *Treponema pallidum*. Dan. Med. J. 62:B5176. 26621404

[ref37] Shabil M. Gaidhane S. Roopashree R. Kaur M. Srivastava M. Barwal A. . (2025). Association of HIV infection and hospitalization among mpox cases: a systematic review and meta-analysis. BMC Infect. Dis. 25:102. doi: 10.1186/s12879-025-10512-6, 39844097 PMC11752846

[ref38] Shin H. Shin H. Rahmati M. Koyanagi A. Jacob L. Smith L. . (2023). Comparison of clinical manifestations in mpox patients living with HIV versus without HIV: a systematic review and meta-analysis. J. Med. Virol. 95:e28713. doi: 10.1002/jmv.28713, 36991570

[ref39] Silva M. S. T. Coutinho C. Torres T. S. Peixoto E. M. Bastos M. O. Mesquita M. B. . (2024). Mpox severity and associated hospitalizations among people with HIV and related immunosuppression in Brazil. AIDS 38, 105–113. doi: 10.1097/QAD.0000000000003748, 37812389 PMC10715691

[ref40] Silva M. S. T. Coutinho C. Torres T. S. Peixoto E. M. Ismério R. Lessa F. . (2022). Ambulatory and hospitalized patients with suspected and confirmed mpox: an observational cohort study from Brazil. Lancet Reg. Health Am. 17:100406. doi: 10.1016/j.lana.2022.100406 36776570 PMC9904017

[ref41] Simpson K. Heymann D. Brown C. S. Edmunds W. J. Elsgaard J. Fine P. . (2020). Human monkeypox - after 40 years, an unintended consequence of smallpox eradication. Vaccine 38, 5077–5081. doi: 10.1016/j.vaccine.2020.04.062, 32417140 PMC9533855

[ref42] Sousa D. Volovetska Y. Nunes D. Lemos C. Borges-Costa J. Filipe P. (2024). Clinical and epidemiological characteristics of Mpox in HIV-infected and uninfected men who have sex with men: a retrospective study in Lisbon. Viruses 16:225. doi: 10.3390/v16020225, 38400001 PMC10892182

[ref43] Spinelli M. A. Lynch K. L. Yun C. Glidden D. V. Peluso M. J. Henrich T. J. . (2021). SARS-CoV-2 seroprevalence, and IgG concentration and pseudovirus neutralising antibody titres after infection, compared by HIV status: a matched case-control observational study. Lancet HIV. 8, e334–e341. doi: 10.1016/S2352-3018(21)00072-2, 33933189 PMC8084354

[ref44] Taha A. M. Elrosasy A. Mahmoud A. Saed S. A. A. Moawad W. A. E. Hamouda E. . (2024). The effect of HIV and mpox co-infection on clinical outcomes: systematic review and meta-analysis. HIV Med. 25, 897–909. doi: 10.1111/hiv.13622, 38443319

[ref45] Tarin-Vicente E. J. Alemany A. Agud-Dios M. Ubals M. Suñe r. C. Antón A. . (2022). Clinical presentation and virological assessment of confirmed human monkeypox virus cases in Spain: a prospective observational cohort study. Lancet 400, 661–669. doi: 10.1016/S0140-6736(22)01436-2, 35952705 PMC9533900

[ref46] Thornhill J. P. Barkati S. Walmsley S. Rockstroh J. Antinori A. Harrison L. B. . (2022). Monkeypox virus infection in humans across 16 countries - April-June 2022. N. Engl. J. Med. 387, 679–691. doi: 10.1056/NEJMoa2207323, 35866746

[ref47] Triana-Gonzalez S. Román-López C. Mauss S. Cano-Díaz A. L. Mata-Marín J. A. Pérez-Barragán E. . (2023). Risk factors for mortality and clinical presentation of monkeypox. AIDS 37, 1979–1985. doi: 10.1097/QAD.0000000000003623, 37294338

[ref48] Vance D. E. McGuinness T. Musgrove K. Orel N. A. Fazeli P. L. (2011). Successful aging and the epidemiology of HIV. Clin. Interv. Aging 6, 181–192. doi: 10.2147/CIA.S14726, 21822373 PMC3147048

[ref49] Wan X. Wang W. Liu J. Tong T. (2014). Estimating the sample mean and standard deviation from the sample size, median, range and/or interquartile range. BMC Med. Res. Methodol. 14:135. doi: 10.1186/1471-2288-14-135, 25524443 PMC4383202

[ref50] World Health Organization. (2022b). Surveillance, case investigation and contact tracing for Monkeypox: interim guidance. Available online at: https://www.who.int/publications/i/item/WHO-MPX-Surveillance-2022.3 (Accessed July 2, 2025).

[ref51] World Health Organization. (2022a). Clinical management and infection prevention and control for monkeypox: interim rapid response guidance. Available online at: https://www.who.int/publications/i/item/WHO-MPX-Clinical-and-IPC-2022.1 (Accessed July 2, 2025).

[ref52] World Health Organization. (2025a). 2022-24 Mpox (Monkeypox) outbreak: global trends. Available online at: https://worldhealthorg.shinyapps.io/mpx_global/ (Accessed July 2, 2025).

[ref53] World Health Organization. (2025b). HIV and AIDS. Available online at: http://www.who.int/news-room/fact-sheets/detail/hiv-aids (Accessed July 2, 2025).

[ref54] Yan J. Zhang Z. An D. Li F. Zheng R. Shi J. . (2024). Clinical characteristics of monkeypox patients with HIV positive or HIV negative. Chin. J. Clin. Infect. Dis. 9:611. doi: 10.3760/cma.j.issn.1674-2397.2023.04.005

[ref55] Yang S. Xia C. Zhang Y. Shen Y. Xia C. Lu Y. . (2024). Clinical features and viral load variations of mpox: a retrospective study in Chongqing, China. BMC Infect. Dis. 24:641. doi: 10.1186/s12879-024-09537-0, 38926635 PMC11202379

[ref56] Yang H. Xie X. Zeng M. Cao Y. Fan Q. Jiang M. . (2024). Clinical characteristics, viral dynamics, and antibody response of monkeypox virus infections among men with and without HIV infection in Guangzhou, China. Front. Cell. Infect. Microbiol. 14:1412753. doi: 10.3389/fcimb.2024.1412753, 38979508 PMC11228139

[ref57] Yang H. Zheng H. Chen X. Tan Y. Zhang F. Wang J. . (2023). Clinical and laboratory characteristics of mpox patients in Guangzhou City. Chin. J. Infect. Dis. 41, 695–700. doi: 10.3760/cma.j.cn311365-20230727-00013

[ref58] Yinka-Ogunleye A. Aruna O. Dalhat M. Ogoina D. McCollum A. Disu Y. . (2019). Outbreak of human monkeypox in Nigeria in 2017-18: a clinical and epidemiological report. Lancet Infect. Dis. 19, 872–879. doi: 10.1016/S1473-3099(19)30294-4, 31285143 PMC9628943

[ref59] Zhao B. Liu Q. Du Q. Kang J. Tang R. Tu Y. . (2024). Characteristics and differences in mpox patients with and without HIV infection: a retrospective cross-sectional study in Chengdu, China. Int. J. Gen. Med. 17, 1381–1393. doi: 10.2147/IJGM.S456198, 38617056 PMC11011692

[ref60] Zhou Q. Yuan X. Xiao G. (2024). Epidemiological and clinical characteristics of 44 cases of mpox in the Changsha City. Chin. J. Zoonoses 40, 893–897. doi: 10.3969/j.issn.1002-2694.2024.00.136

