# Supplementary material for: The impact of human immunodeficiency virus coinfection on mpox patients during the 2022 global outbreak: a systematic review and meta-analysis based on comparative observational studies
Source: Front Microbiol. 2026 Feb 9;17:1751445. doi: 10.3389/fmicb.2026.1751445 (PMC12964369; doi:10.3389/fmicb.2026.1751445)
Supplement: Supplementary file 1 [file Table_1.DOC]

**Supplementary Materials**

**Table S1 : Newcastle–Ottawa quality assessment scale.**

| **Study** | **Publication year** | **Selection** | **Comparability** | **Outcome** | **Quality assessment** |
| --- | --- | --- | --- | --- | --- |
| Silva et al. | 2022 | 4 | 1 | 3 | good |
| Li et al. | 2024 | 4 | 1 | 3 | good |
| Aldred et al. | 2024 | 4 | 1 | 3 | good |
| Zhao et al. | 2024 | 4 | 2 | 3 | good |
| Estevez et al. | 2023 | 4 | 1 | 3 | good |
| Sousa et al. | 2024 | 4 | 1 | 3 | good |
| Caria et al. | 2022 | 4 | 1 | 3 | good |
| Hoffmann et al. | 2023 | 4 | 1 | 3 | good |
| Yang et al. | 2024 | 4 | 1 | 3 | good |
| Lim et al. | 2024 | 4 | 2 | 3 | good |
| Jin et al. | 2025 | 4 | 1 | 3 | good |
| Angelo et al. | 2023 | 4 | 1 | 3 | good |
| Curran et al. | 2022 | 4 | 1 | 3 | good |
| Yan et al. | 2024 | 4 | 1 | 3 | good |
| Alpalhao et al. | 2023 | 4 | 1 | 1 | **poor** |
| Agrati et al. | 2023 | 4 | 1 | 1 | **poor** |
| Silva et al. | 2024 | 4 | 1 | 3 | good |
| Corma-Gomez et al. | 2024 | 4 | 1 | 3 | good |
| Betancort-Plata et al. | 2022 | 4 | 1 | 3 | good |
| Lozada et al. | 2025 | 4 | 1 | 3 | good |
| Guo et al. | 2024 | 4 | 2 | 3 | good |
| Nunez et al. | 2024 | 4 | 1 | 3 | good |
| Pilkington et al. | 2023 | 4 | 1 | 3 | good |
| Kowalski et al. | 2023 | 4 | 1 | 3 | good |
| Fu et al. | 2023 | 4 | 1 | 3 | good |
| Moraes-Cardoso et al. | 2024 | 4 | 1 | 3 | good |
| Triana-Gonzalez et al. | 2023 | 4 | 2 | 3 | good |
| Hu et al. | 2025 | 4 | 1 | 3 | good |
| Ramirez-Soto et al. | 2024 | 4 | 1 | 3 | good |


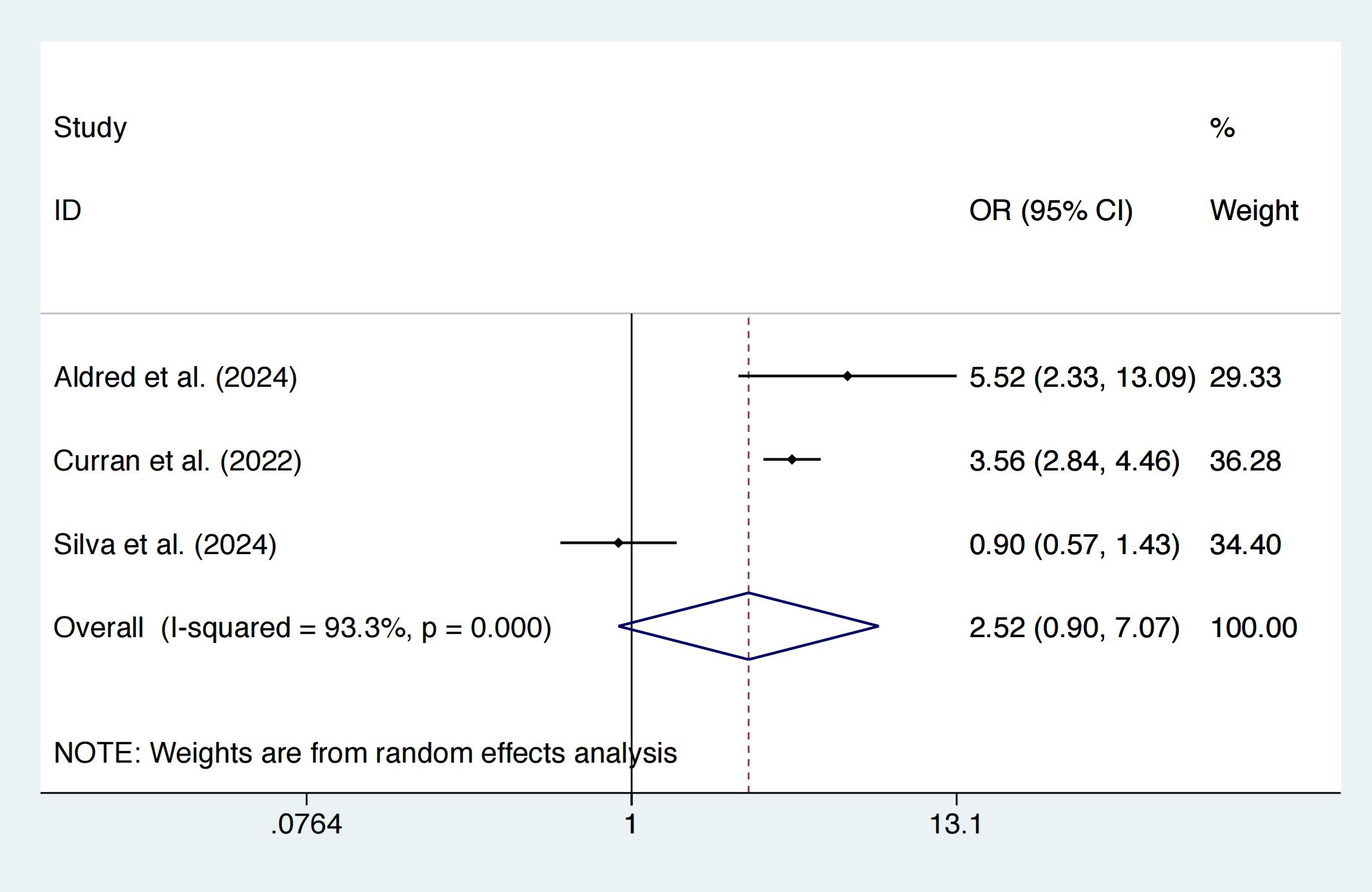


**Figure S1: Forest plot of differences in the race between the HIV-positive group and the HIV-negative group: Black.**

**
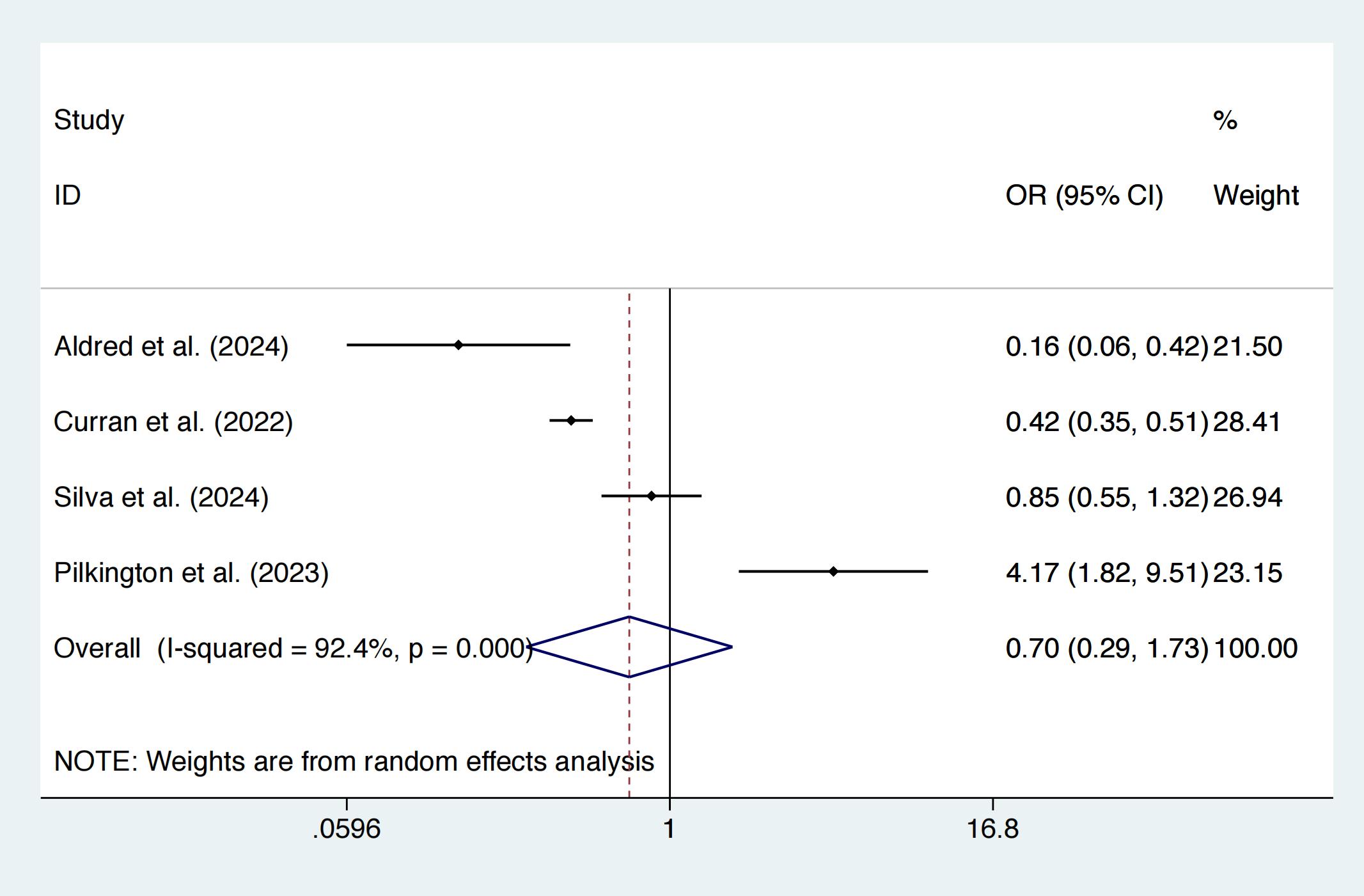
**

**Figure S2: Forest plot of differences in the race between the HIV-positive group and the HIV-negative group: White.**

**
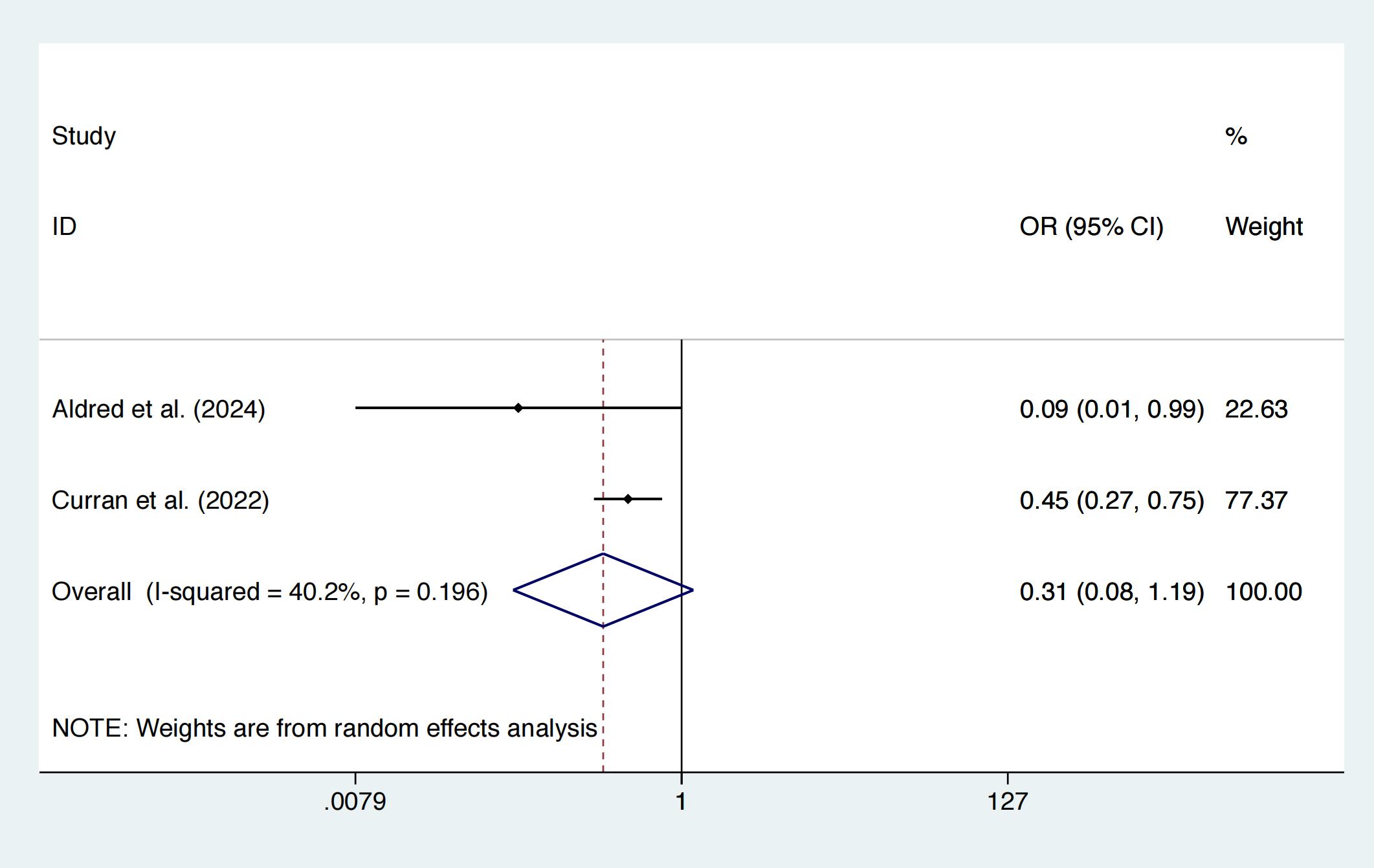
**

**Figure S3: Forest plot of differences in the race between the HIV-positive group and the HIV-negative group: Asian.**

**
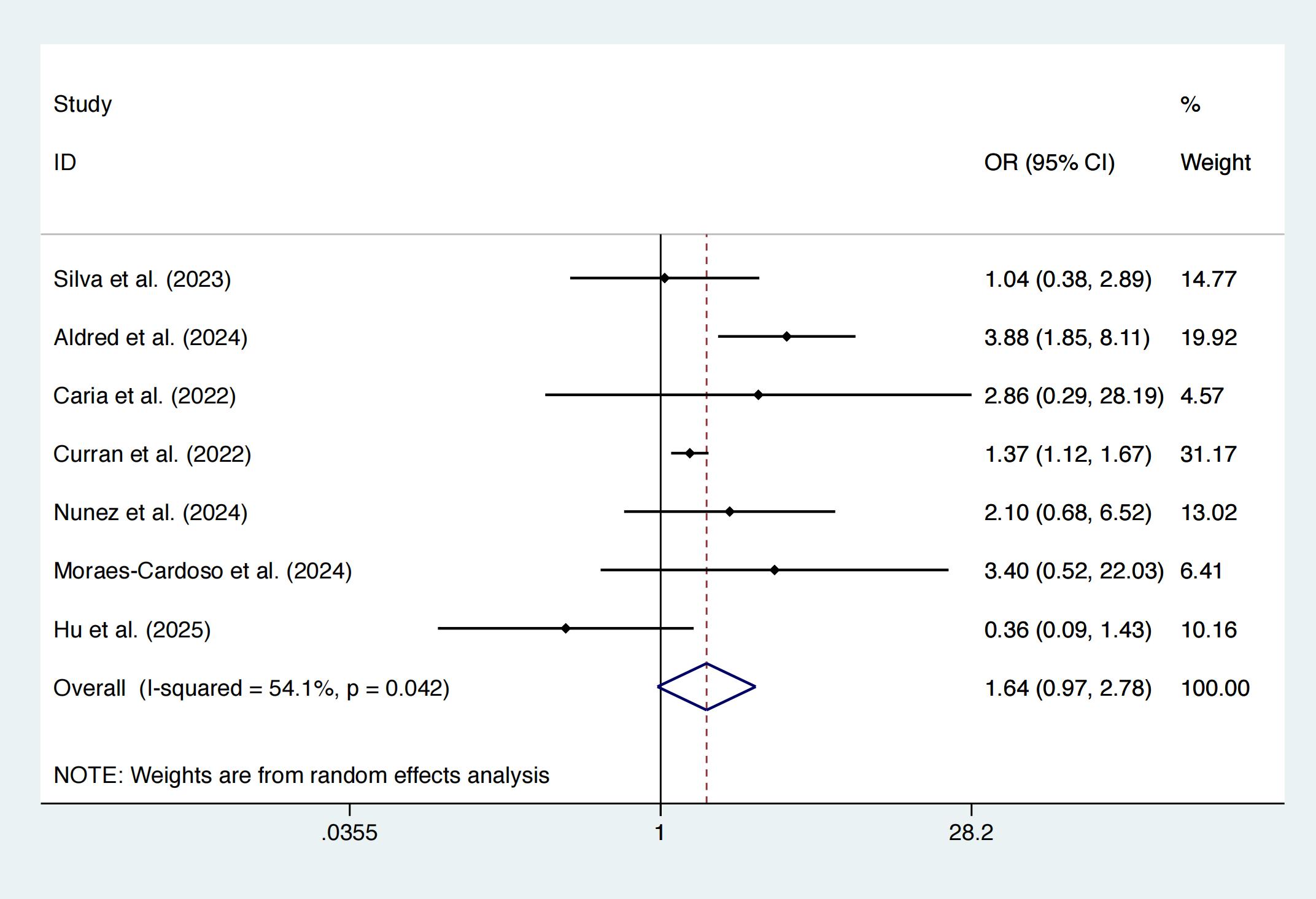
**

**Figure S4: Forest plot of differences in the sexually transmitted infection between the HIV-positive group and the HIV-negative group: gonorrhea.**

**
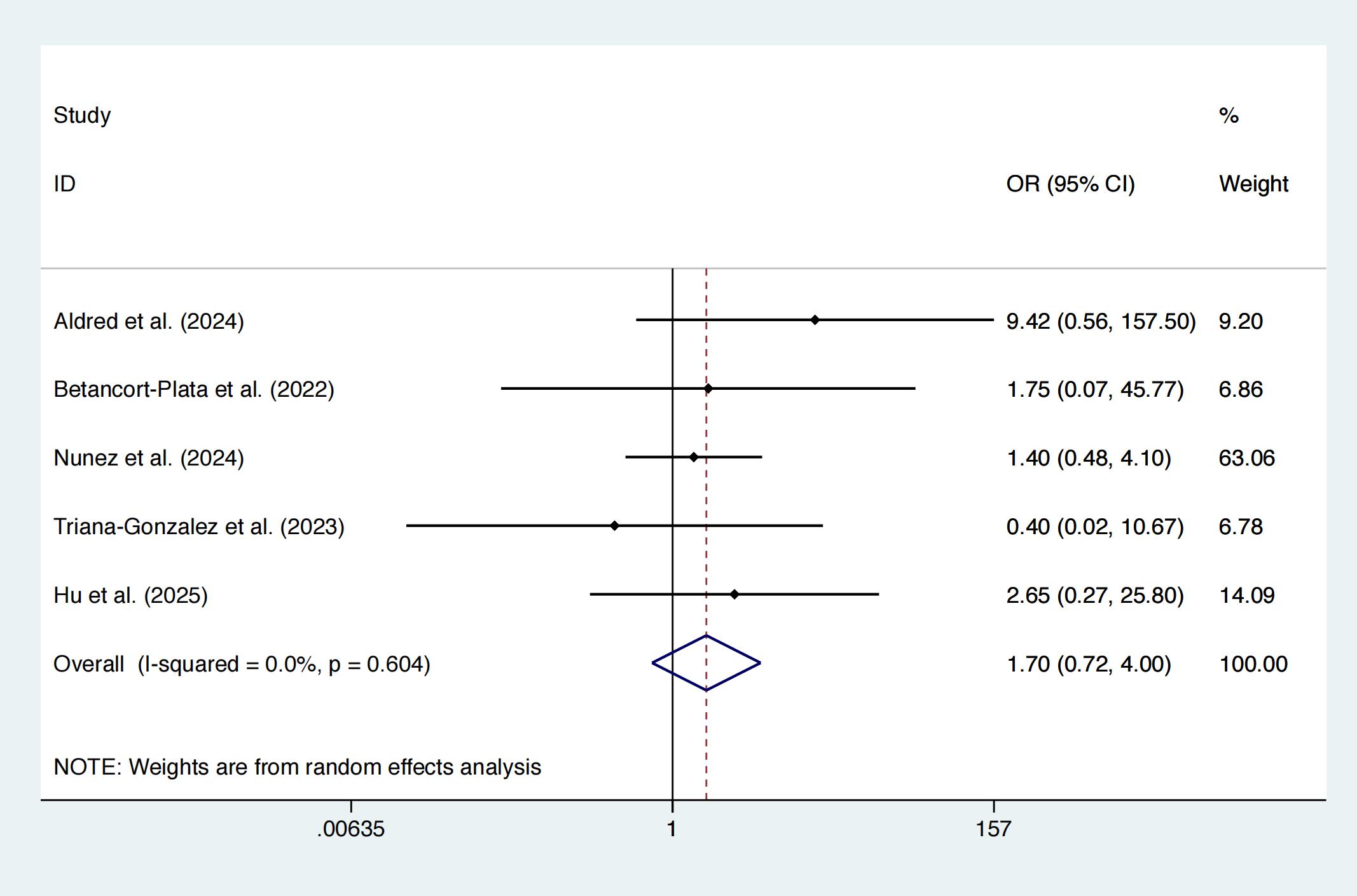
**

**Figure S5: Forest plot of differences in the sexually transmitted infection between the HIV-positive group and the HIV-negative group: herpes.**

**
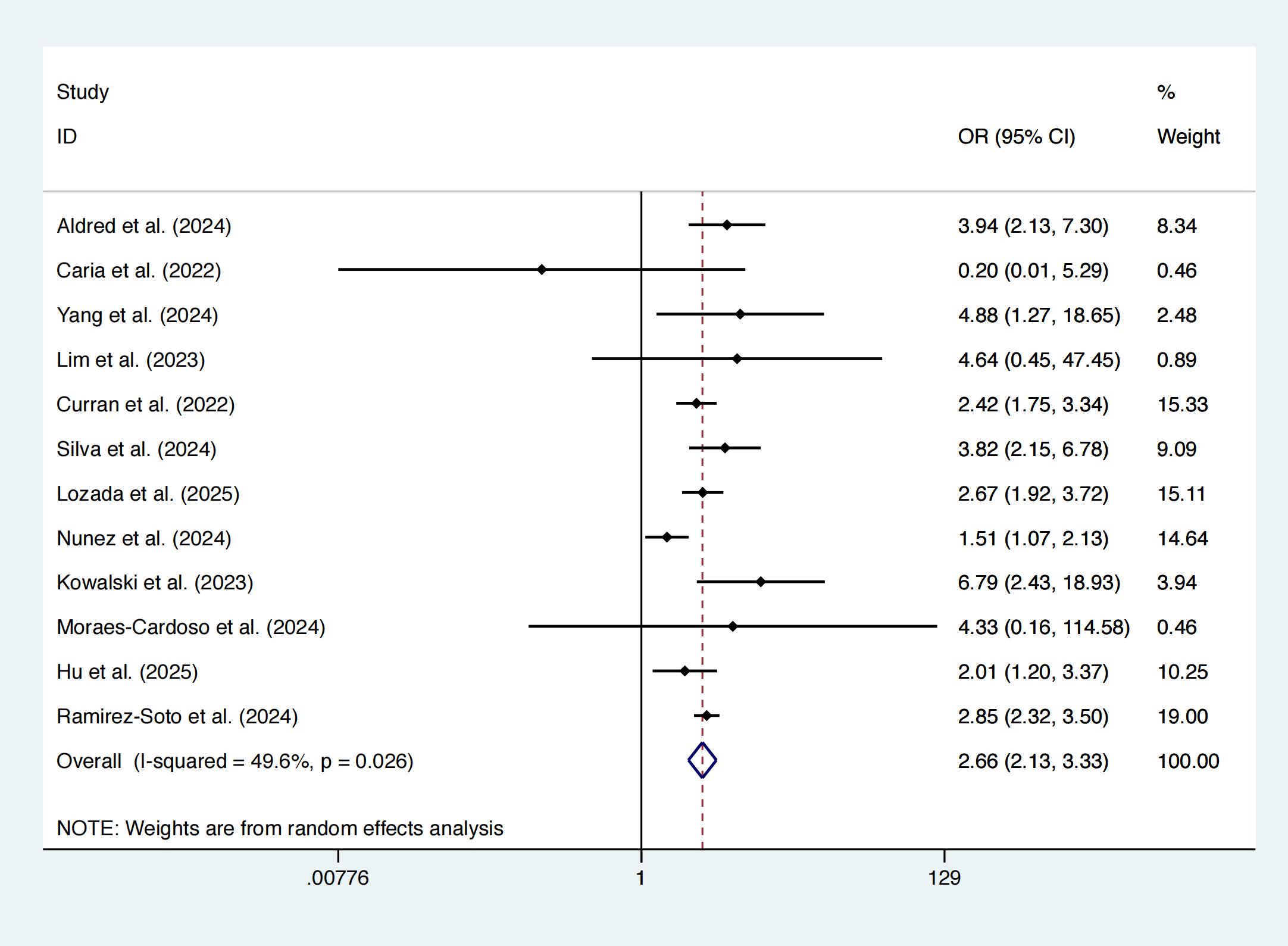
**

**Figure S6: Forest plot of differences in the sexually transmitted infection between the HIV-positive group and the HIV-negative group: syphilis.**

**
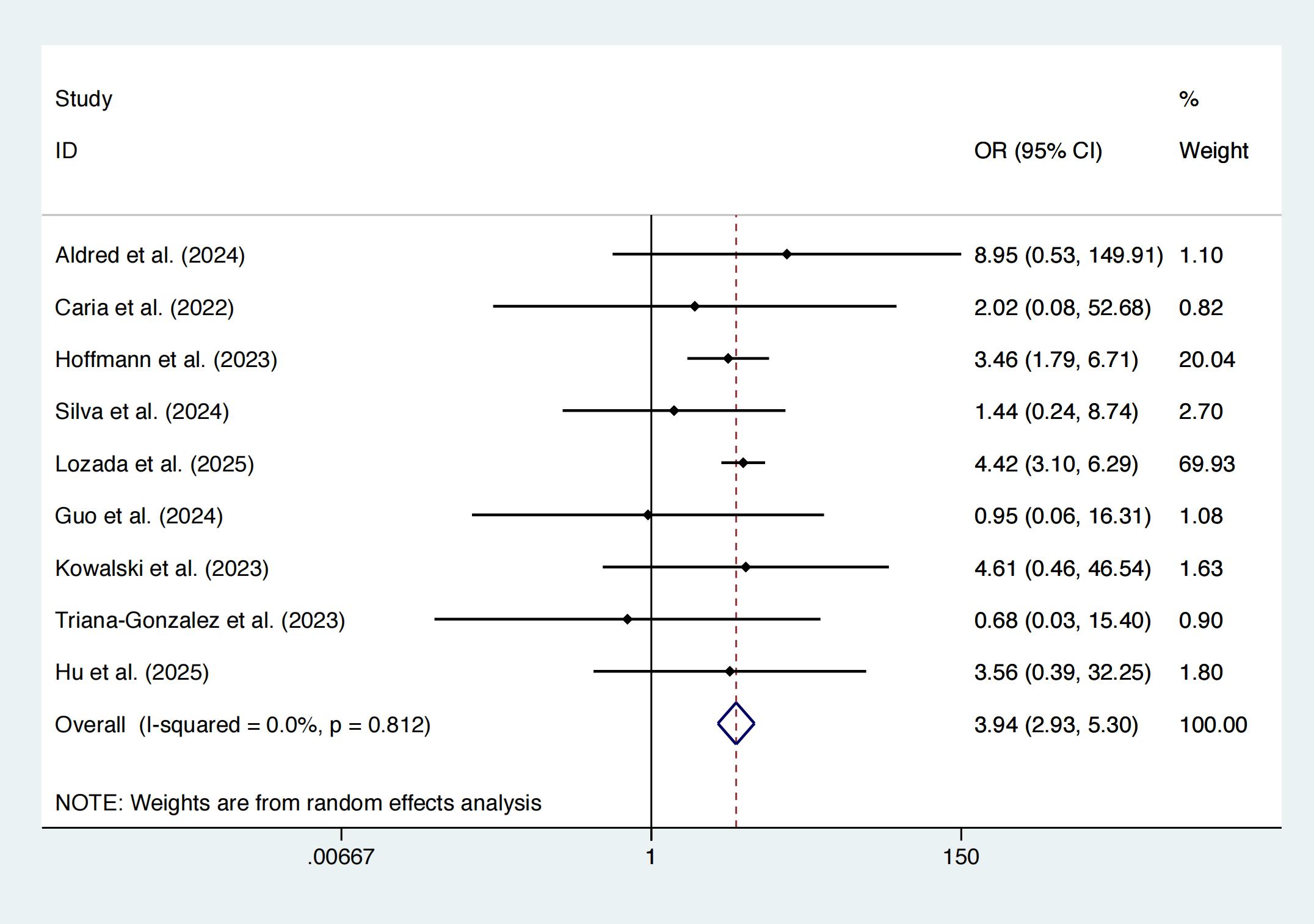
**

**Figure S7: Forest plot of differences in the sexually transmitted infection between the HIV-positive group and the HIV-negative group: hepatitis B.**

**
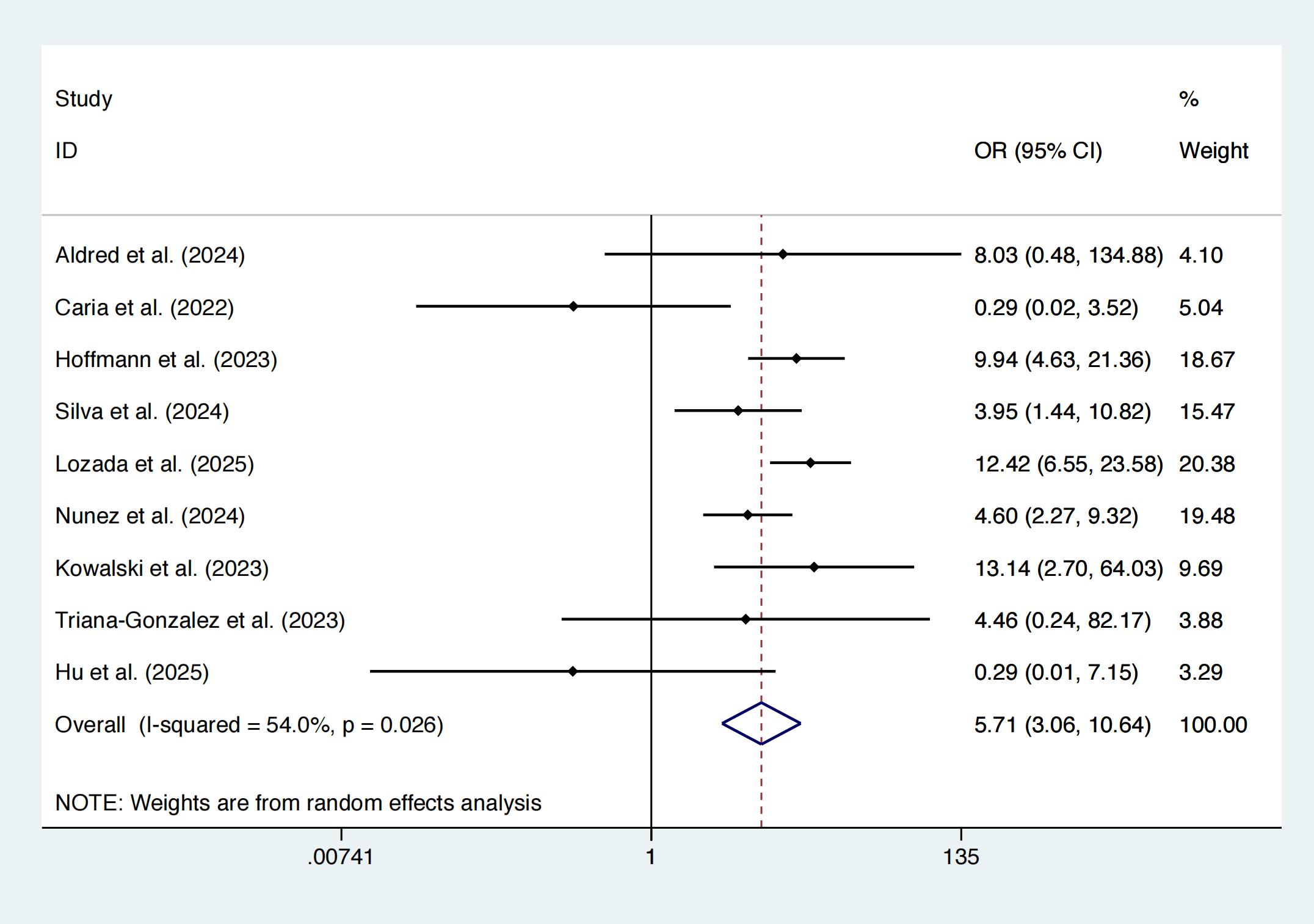
**

**Figure S8: Forest plot of differences in the sexually transmitted infection between the HIV-positive group and the HIV-negative group: hepatitis C.**

**
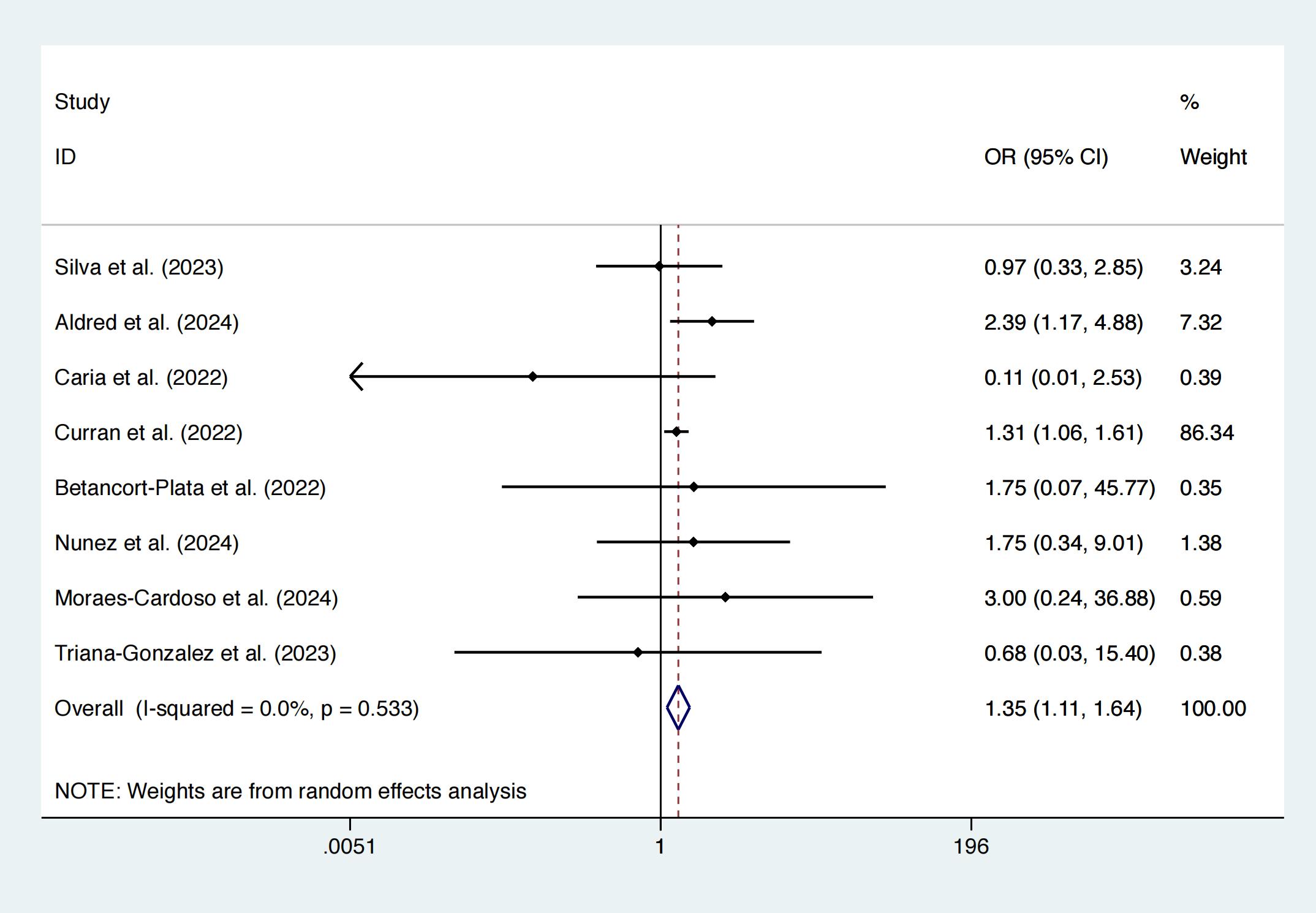
**

**Figure S9: Forest plot of differences in the sexually transmitted infection between the HIV-positive group and the HIV-negative group: chlamydia.**

**
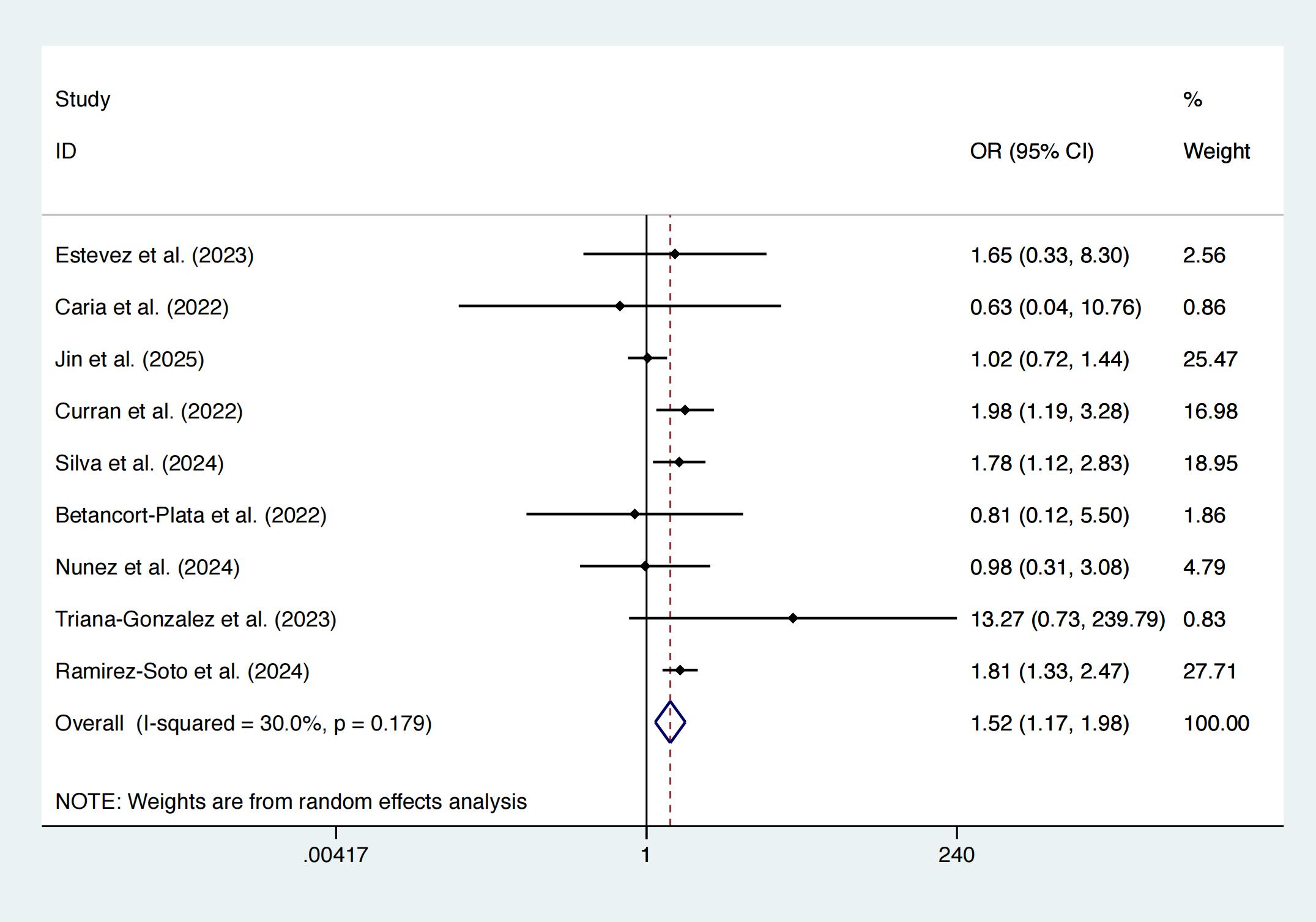
**

**Figure S10: Forest plot of differences in the complications between the HIV-positive group and the HIV-negative group: proctitis.**

**
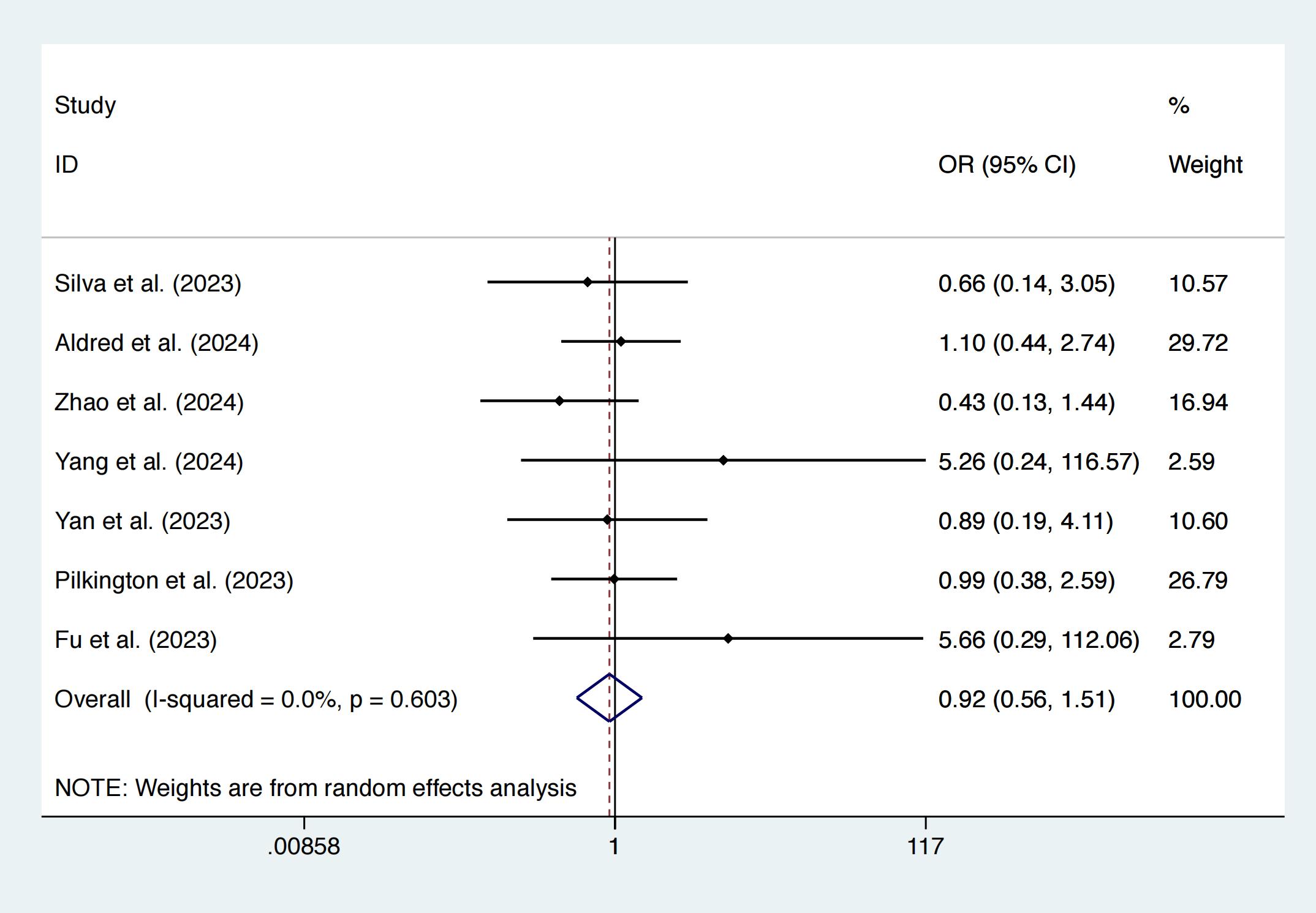
**

**Figure S11: Forest plot of differences in the complications between the HIV-positive group and the HIV-negative group: bacterial infection.**

**
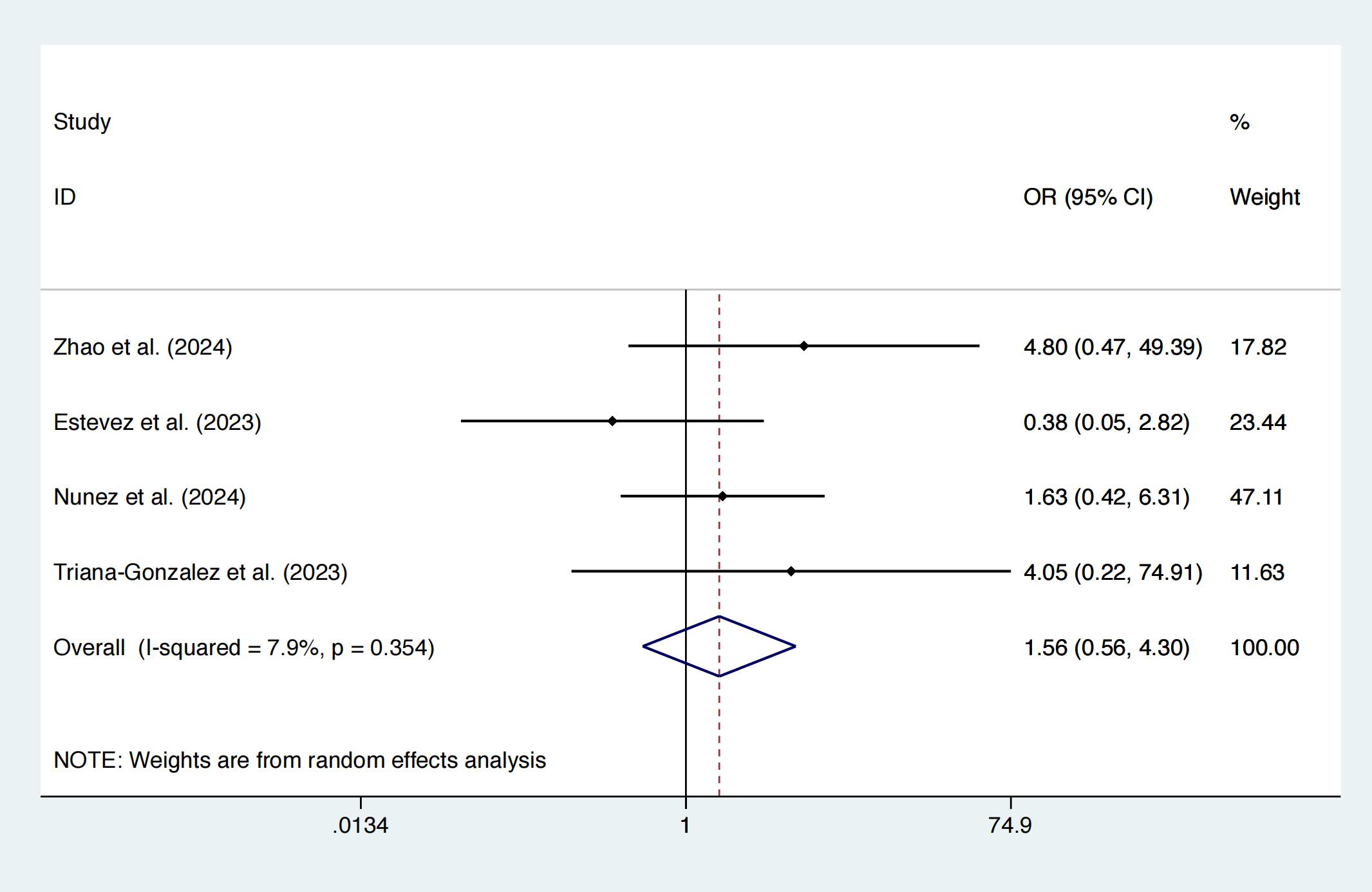
**

**Figure S12: Forest plot of differences in the complications between the HIV-positive group and the HIV-negative group: urethritis.**

**
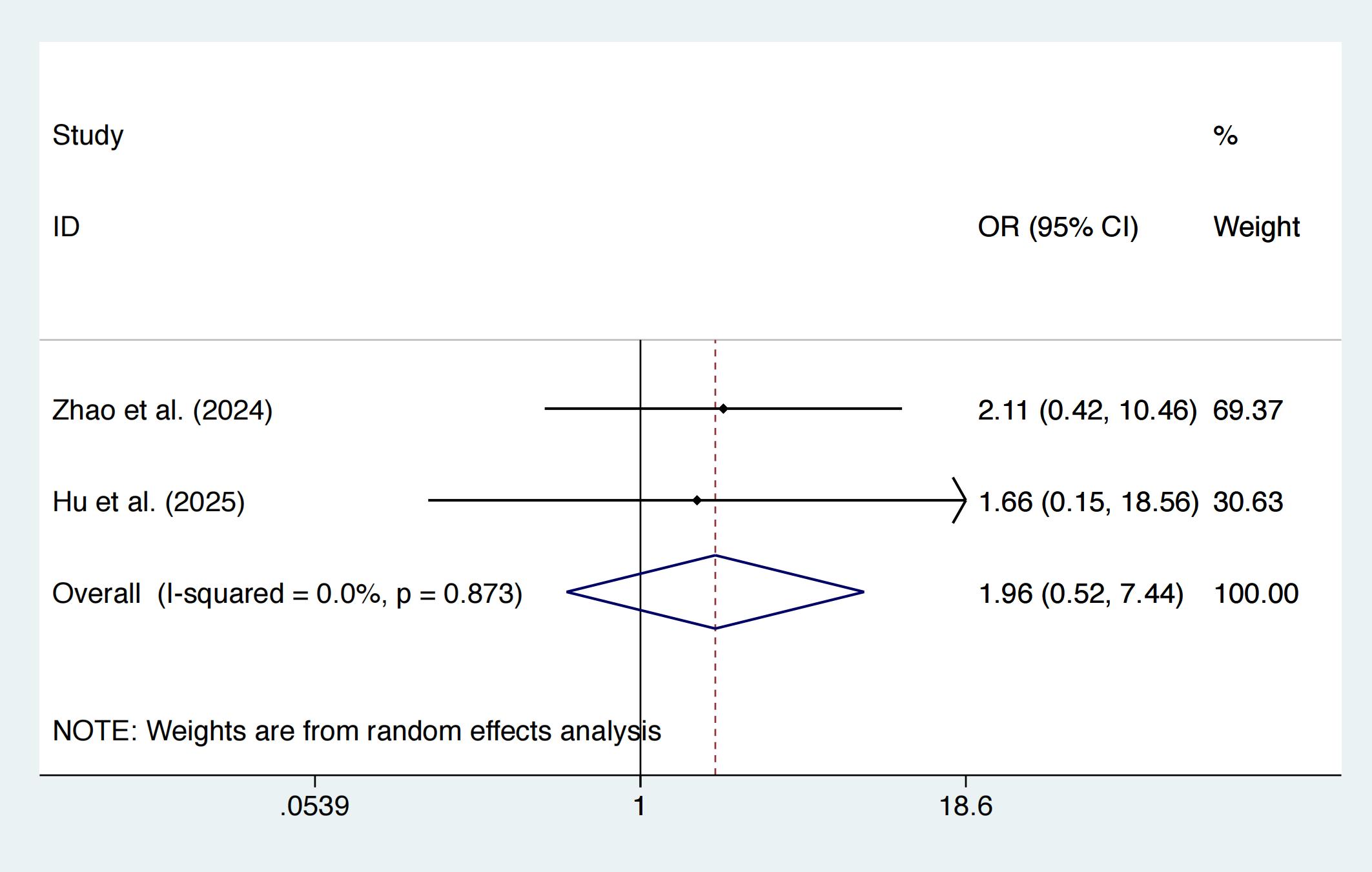
**

**Figure S13: Forest plot of differences in the complications between the HIV-positive group and the HIV-negative group: tonsillitis.**

**
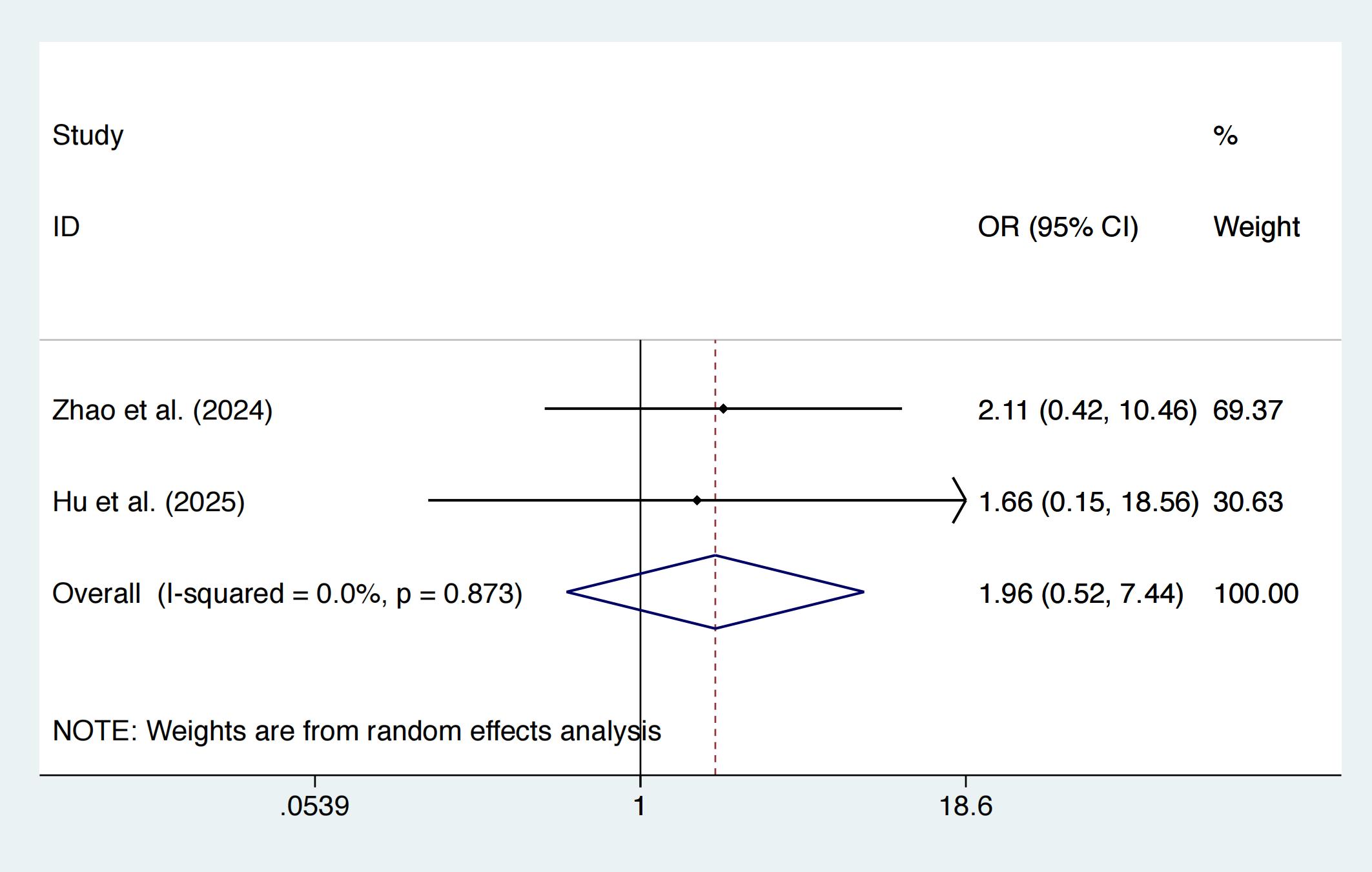
**

**Figure S14: Forest plot of differences in the complications between the HIV-positive group and the HIV-negative group: pneumonia.**

**Figure S15: Forest plot of differences in the symptoms between the HIV-positive group and the HIV-negative group: fever.**

**
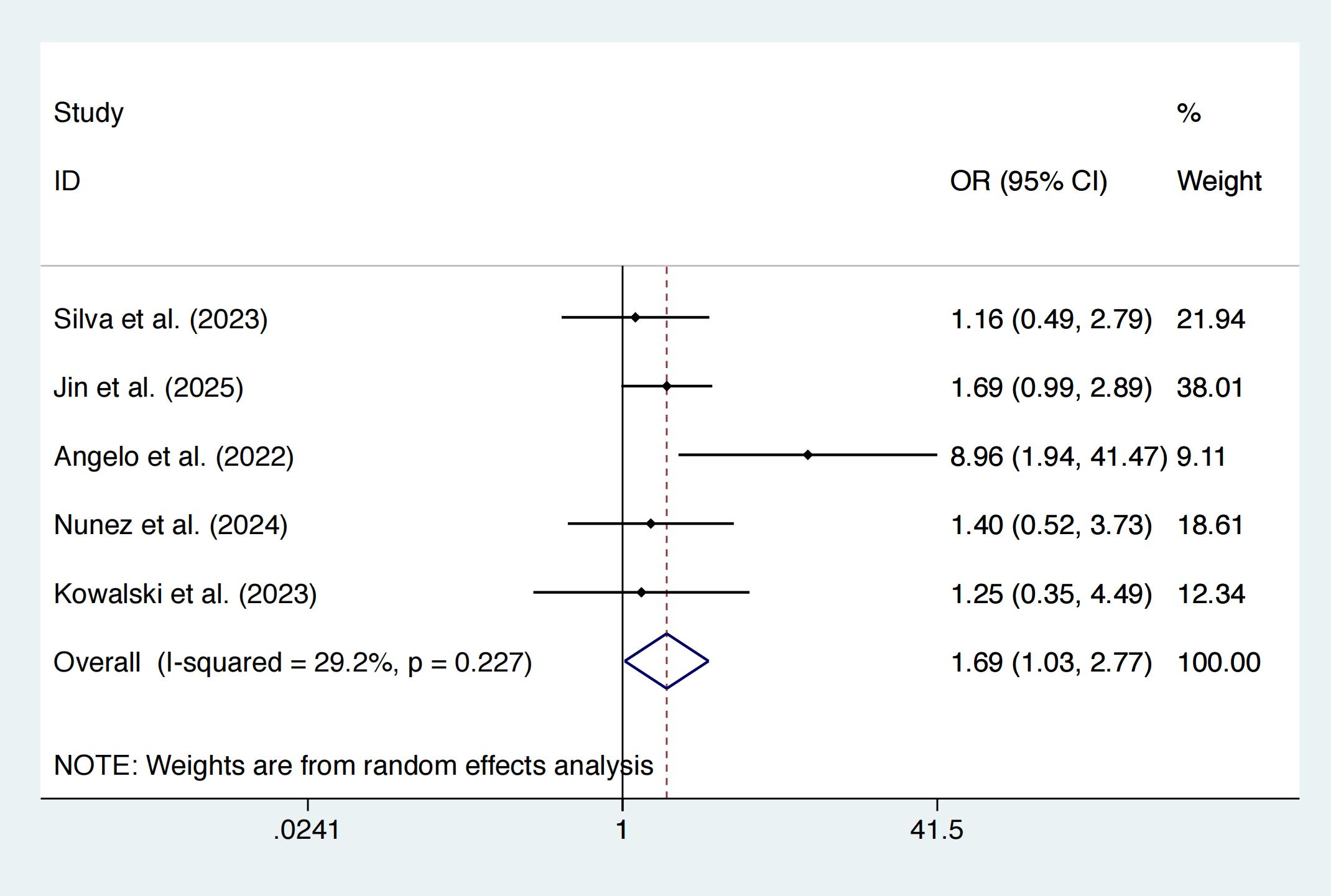
**

**Figure S16: Forest plot of differences in the symptoms between the HIV-positive group and the HIV-negative group: diarrhea.**

**
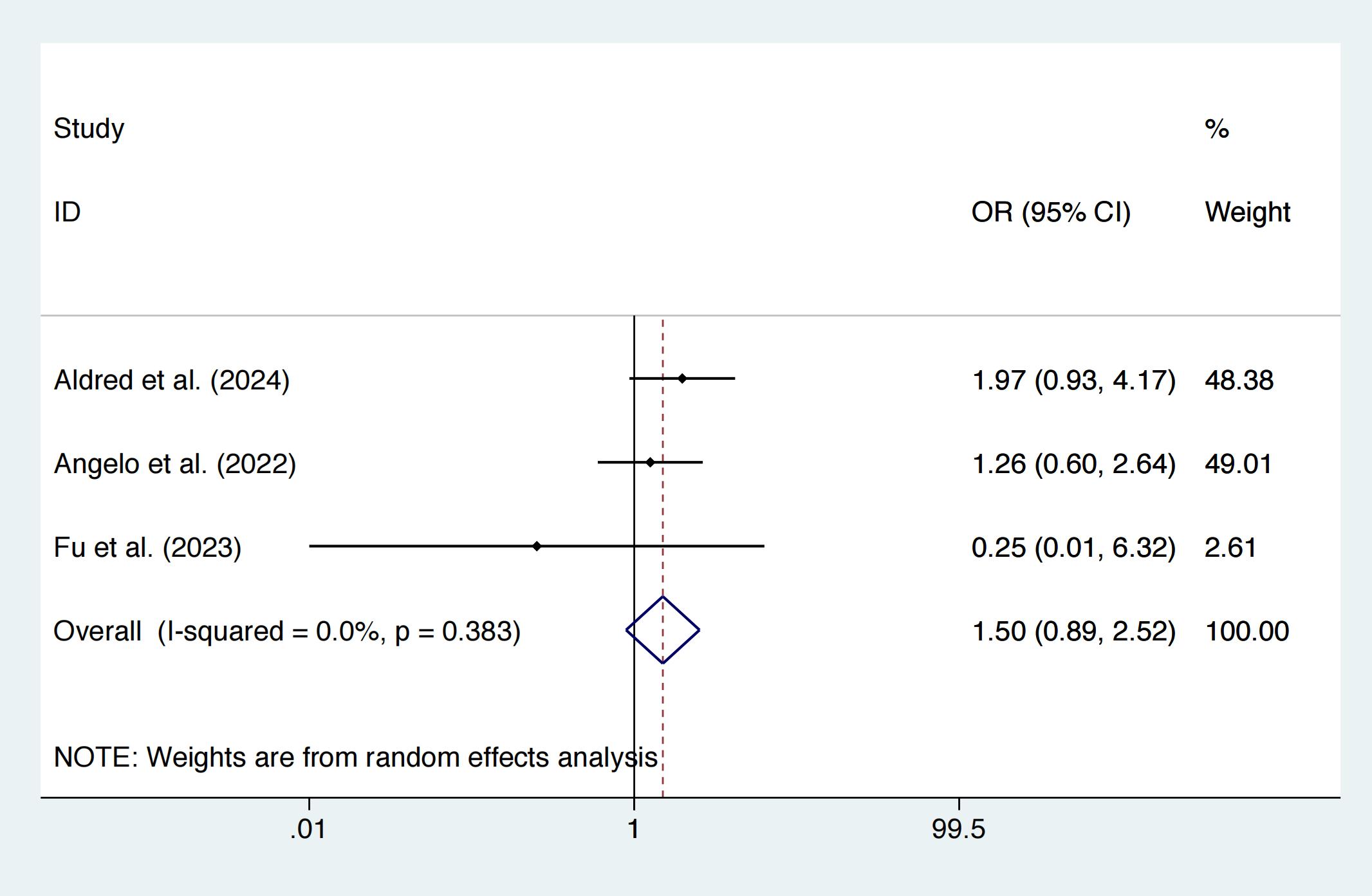
**

**Figure S17: Forest plot of differences in the symptoms between the HIV-positive group and the HIV-negative group: rectal pain.**

**
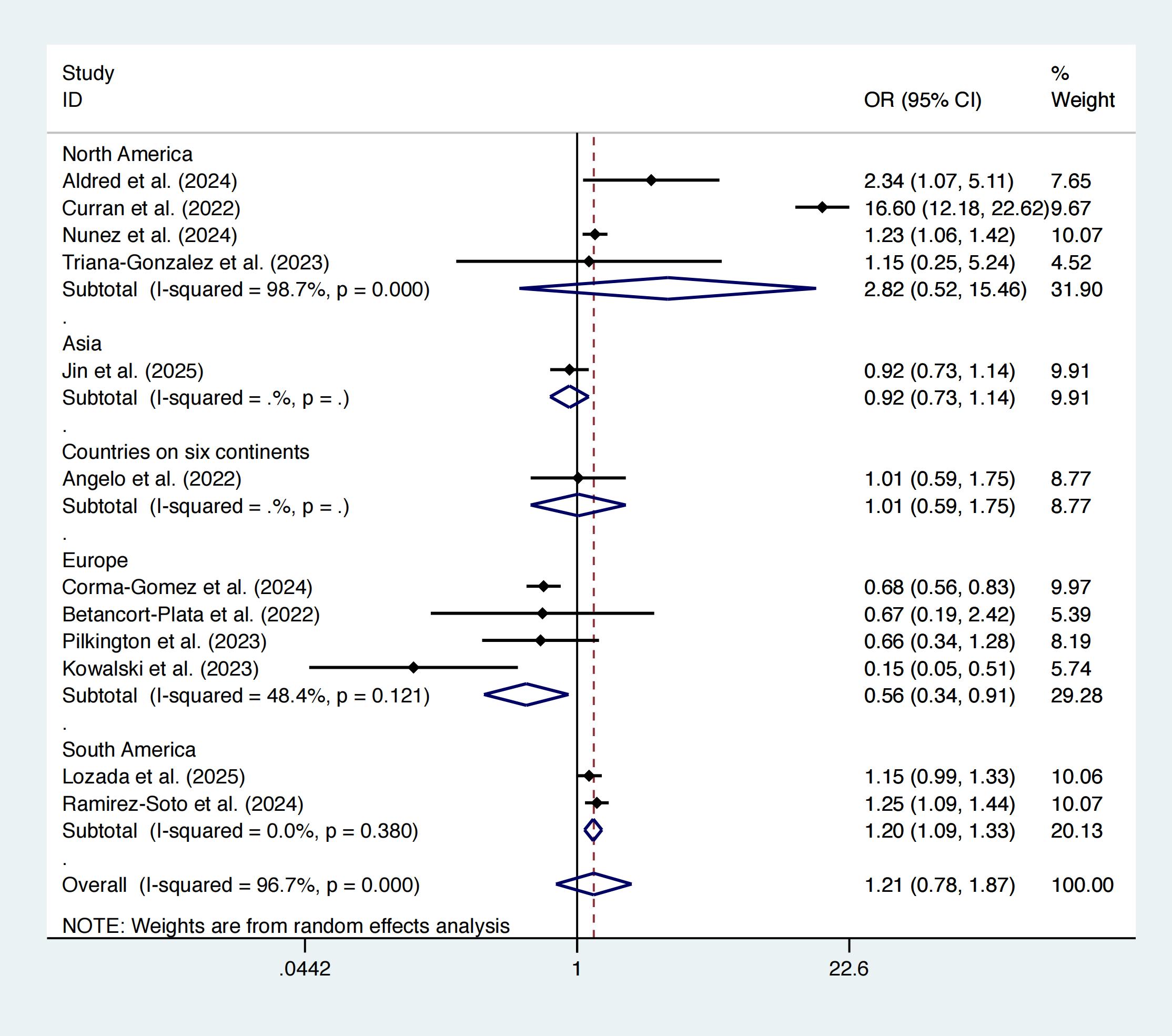
**

**Figure S18: Forest plot of differences in the symptoms between the HIV-positive group and the HIV-negative group: lymphadenopathy.**

**Figure S19: Forest plot of differences in the symptoms between the HIV-positive group and the HIV-negative group: headache.**

**
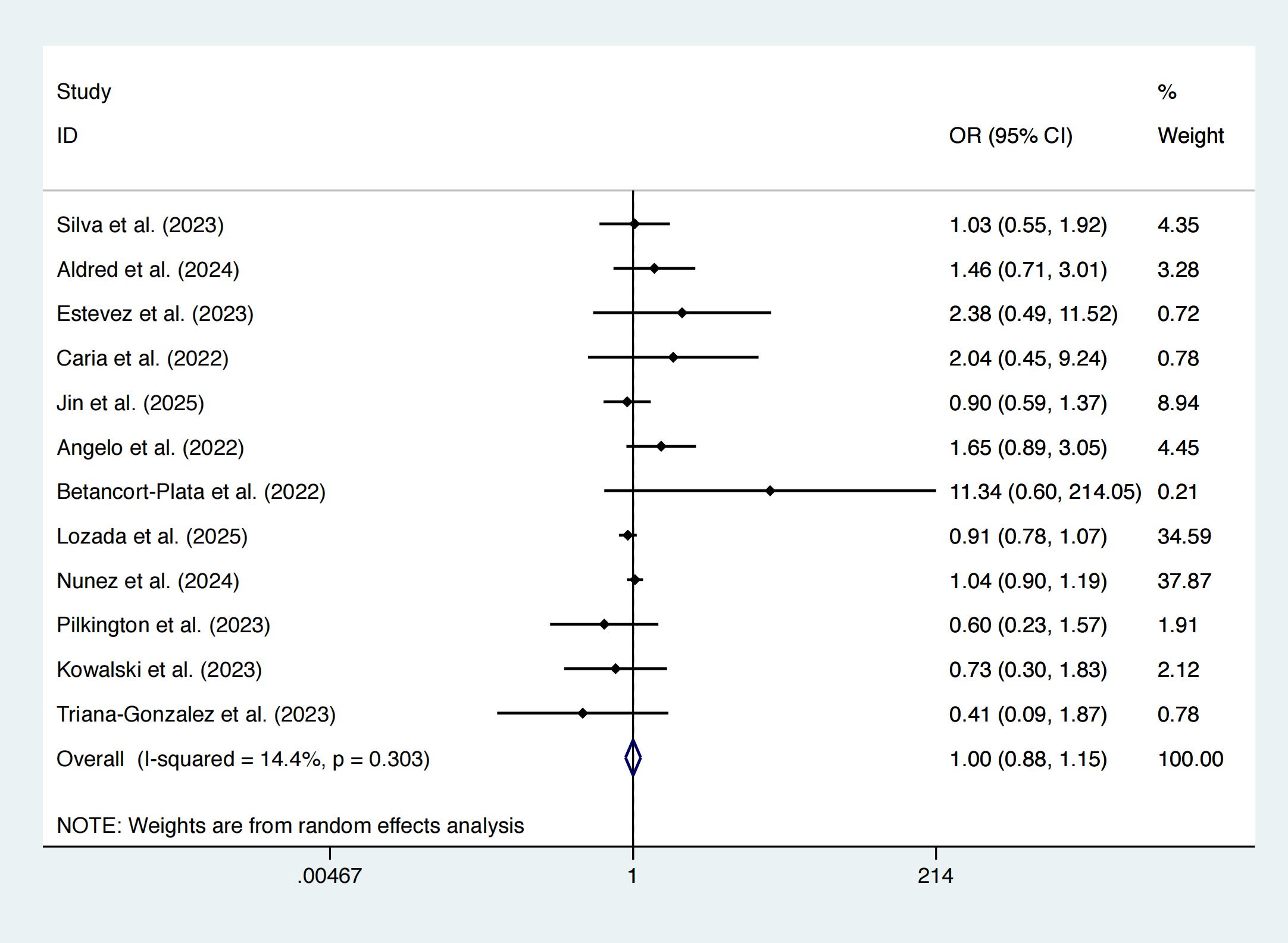
**

**Figure S20: Forest plot of differences in the symptoms between the HIV-positive group and the HIV-negative group: sore throat.**

**
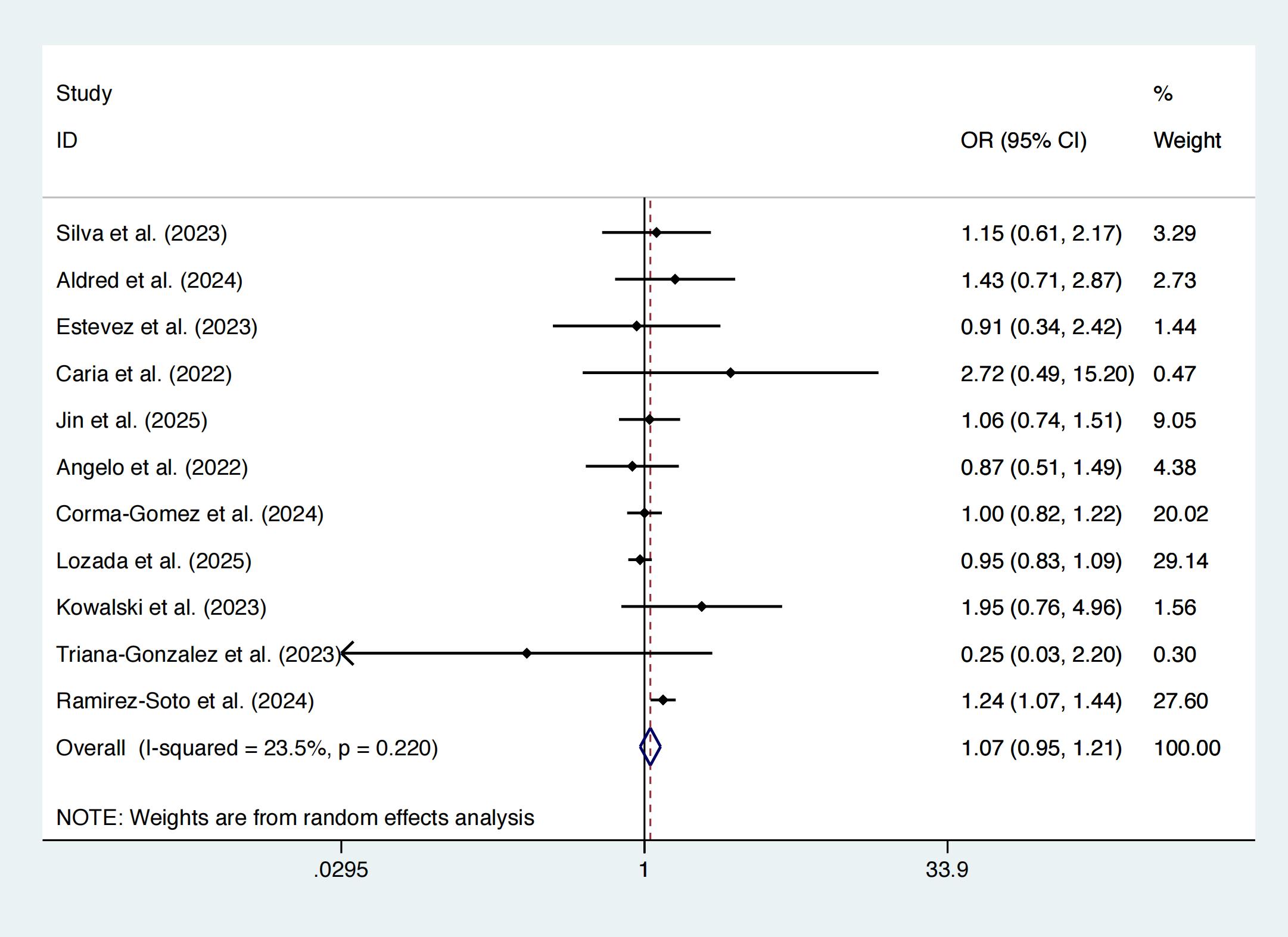
**

**Figure S21: Forest plot of differences in the symptoms between the HIV-positive group and the HIV-negative group: asthenia.**

**
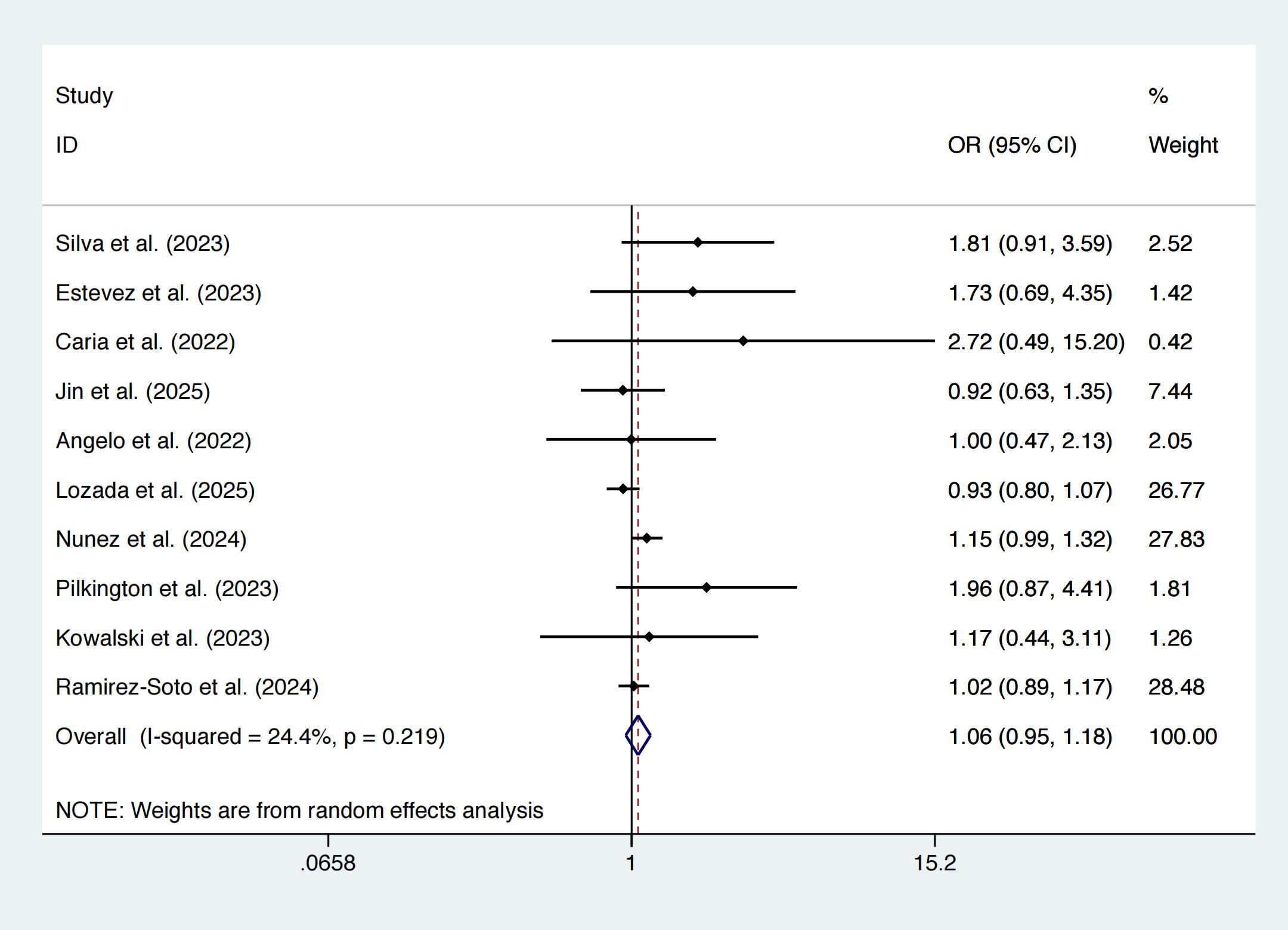
**

**Figure S22: Forest plot of differences in the symptoms between the HIV-positive group and the HIV-negative group: myalgia.**

**
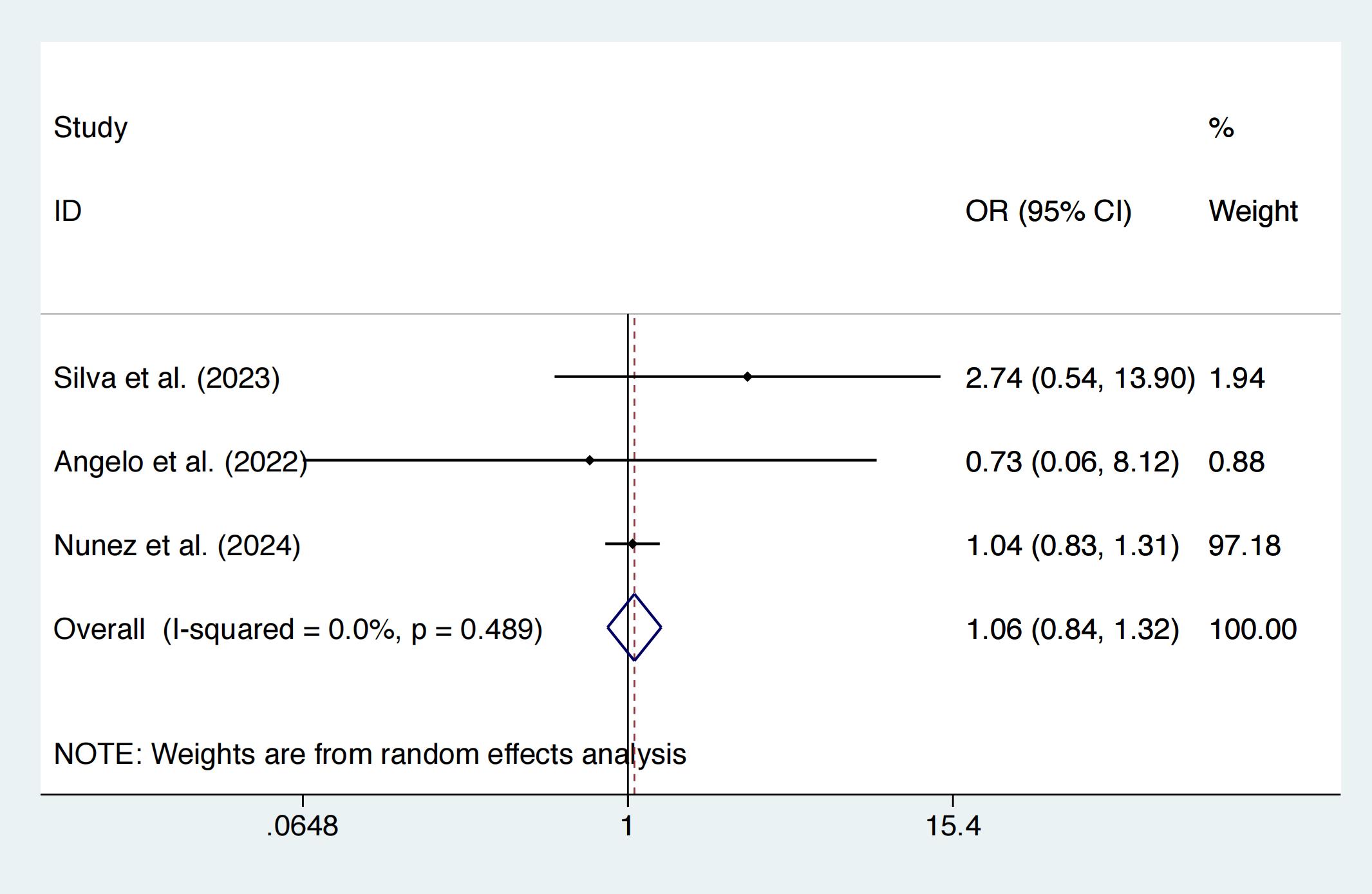
**

**Figure S23: Forest plot of differences in the symptoms between the HIV-positive group and the HIV-negative group: nausea.**

**
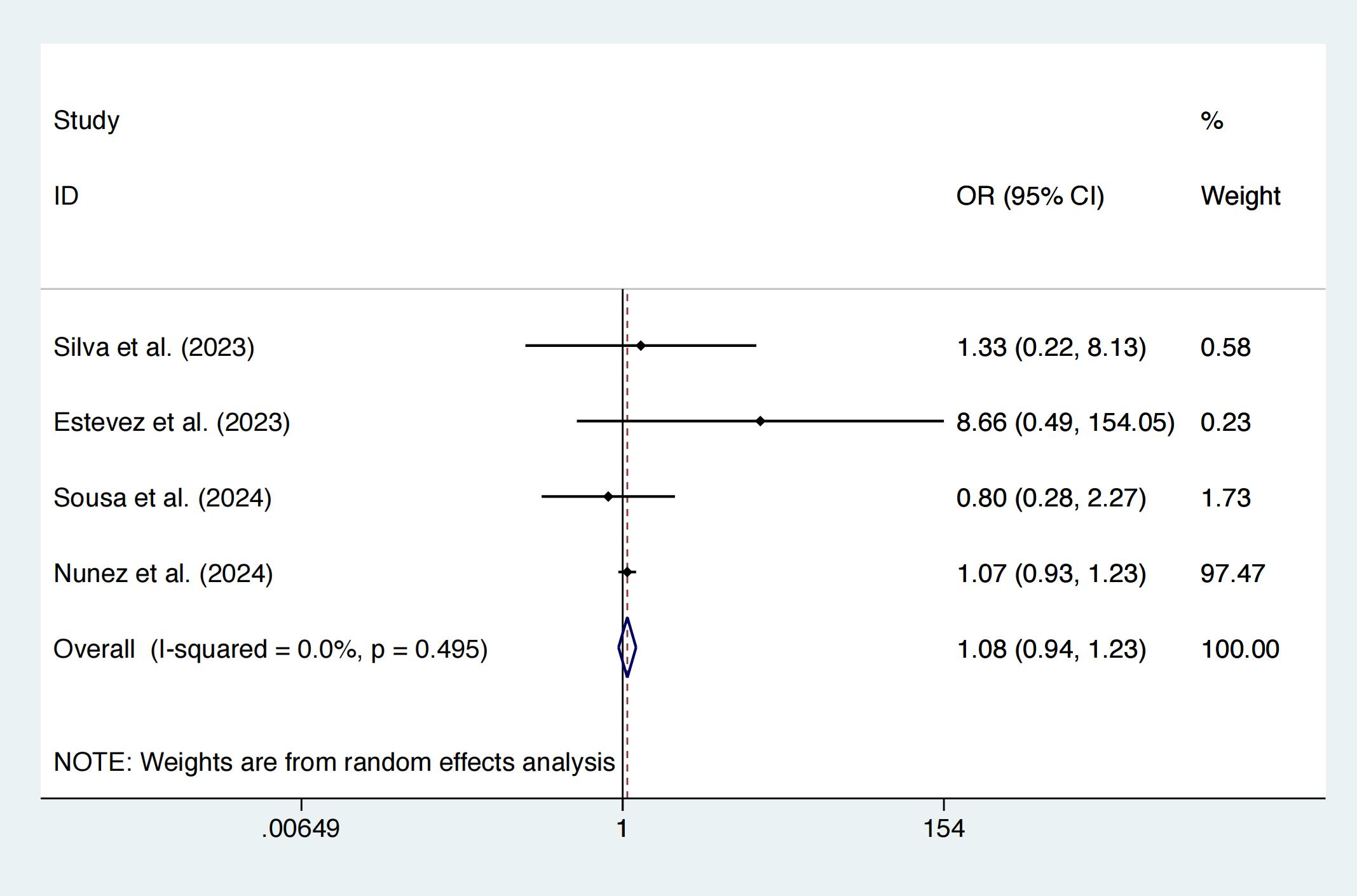
**

**Figure S24: Forest plot of differences in the symptoms between the HIV-positive group and the HIV-negative group: arthragia.**

**
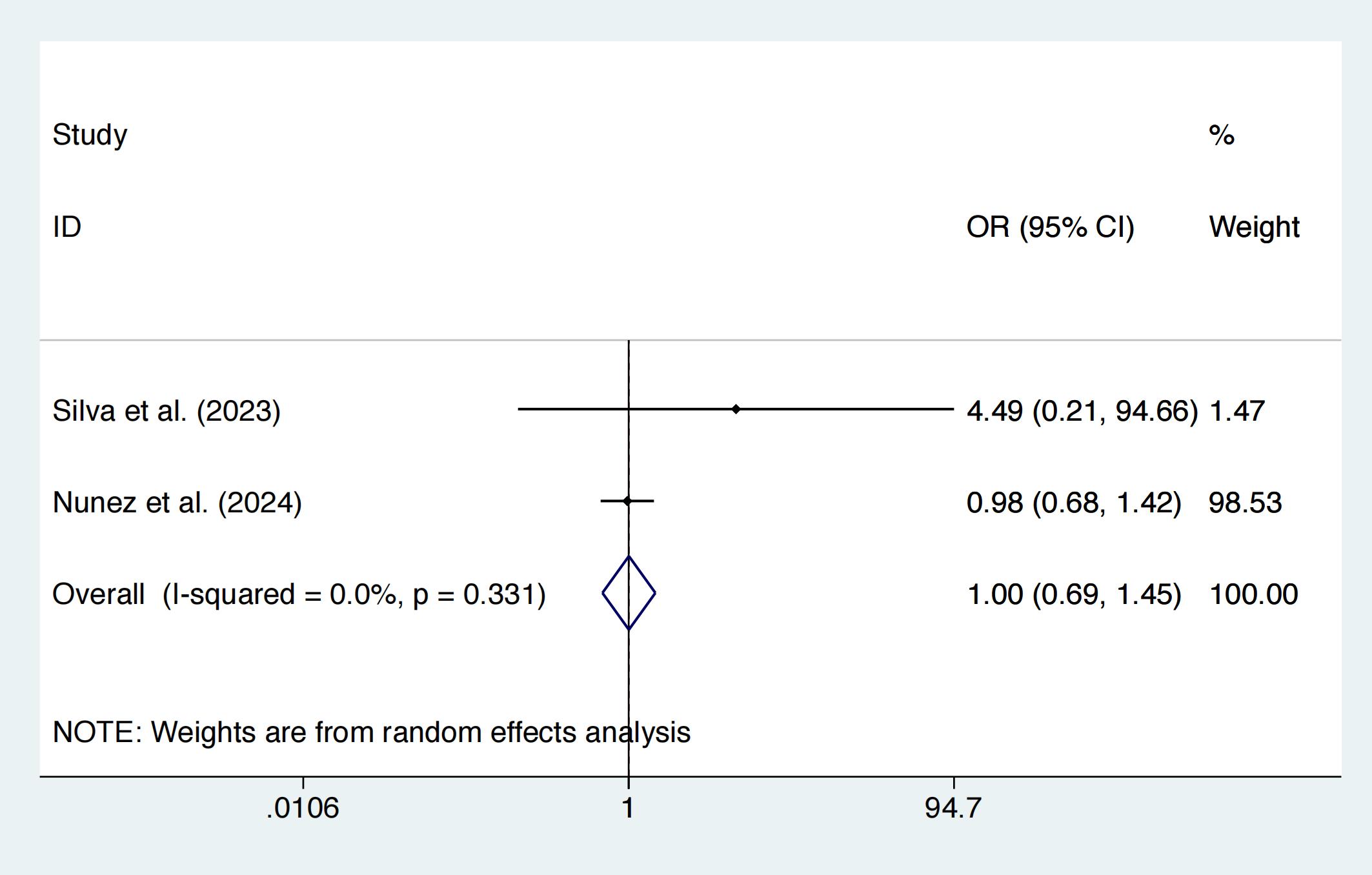
**

**Figure S25: Forest plot of differences in the symptoms between the HIV-positive group and the HIV-negative group: vomiting.**

**
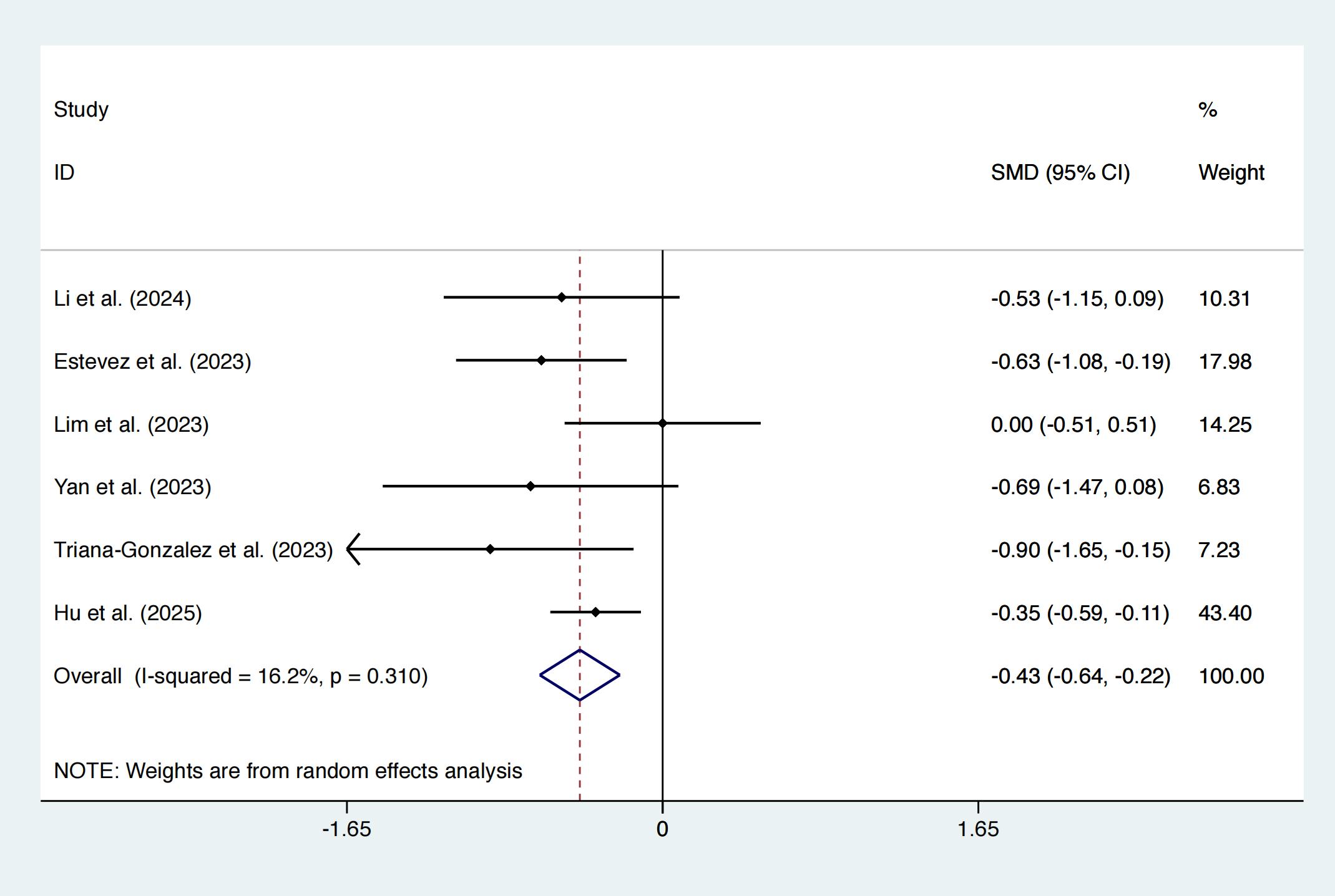
**

**Figure S26: Forest plot of differences in the laboratory findings between the HIV-positive group and the HIV-negative group: hemoglobin.**

**
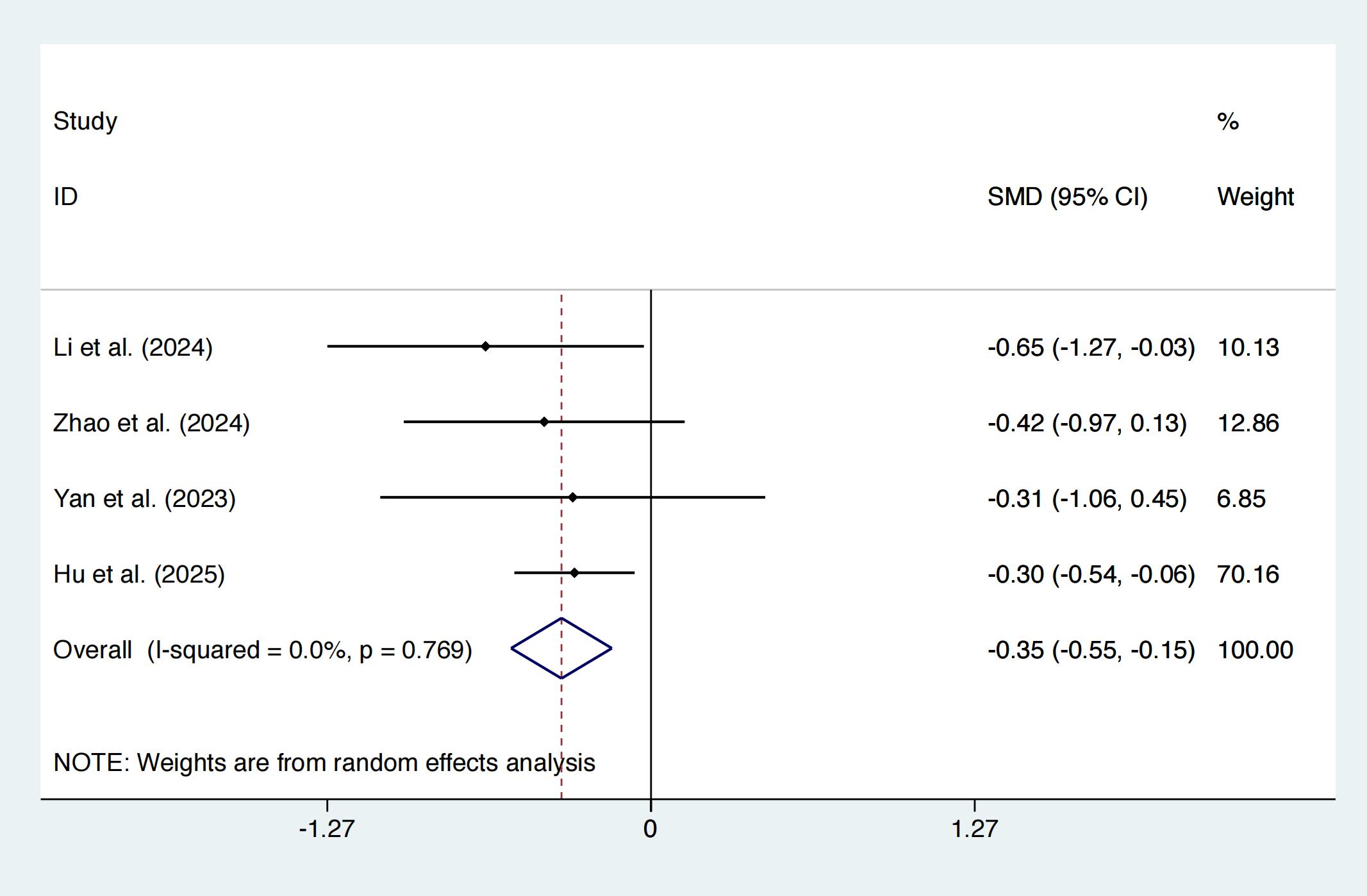
**

**Figure S27: Forest plot of differences in the laboratory findings between the HIV-positive group and the HIV-negative group: albumin.**

**
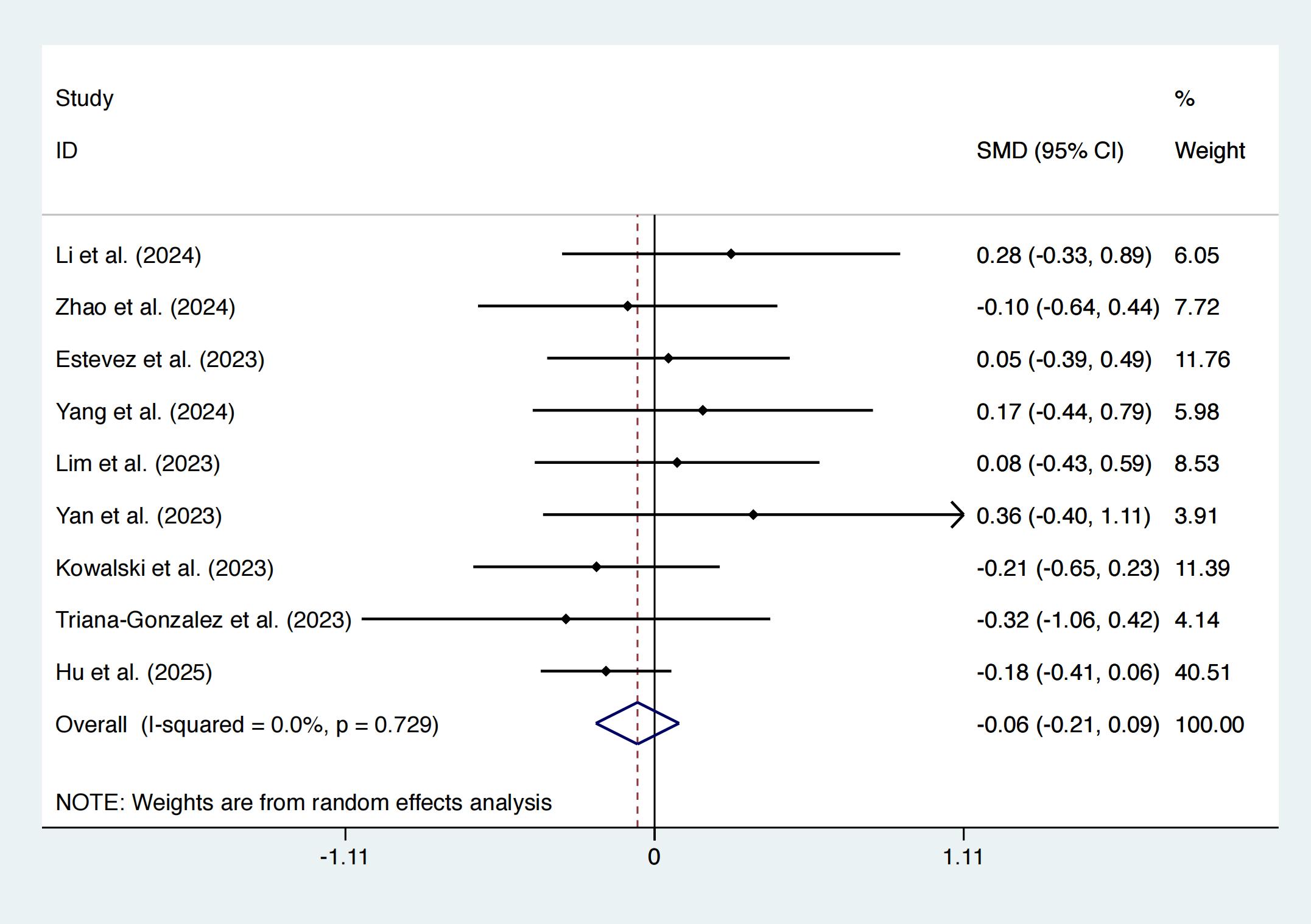
**

**Figure S28: Forest plot of differences in the laboratory findings between the HIV-positive group and the HIV-negative group: white blood cell count.**

**
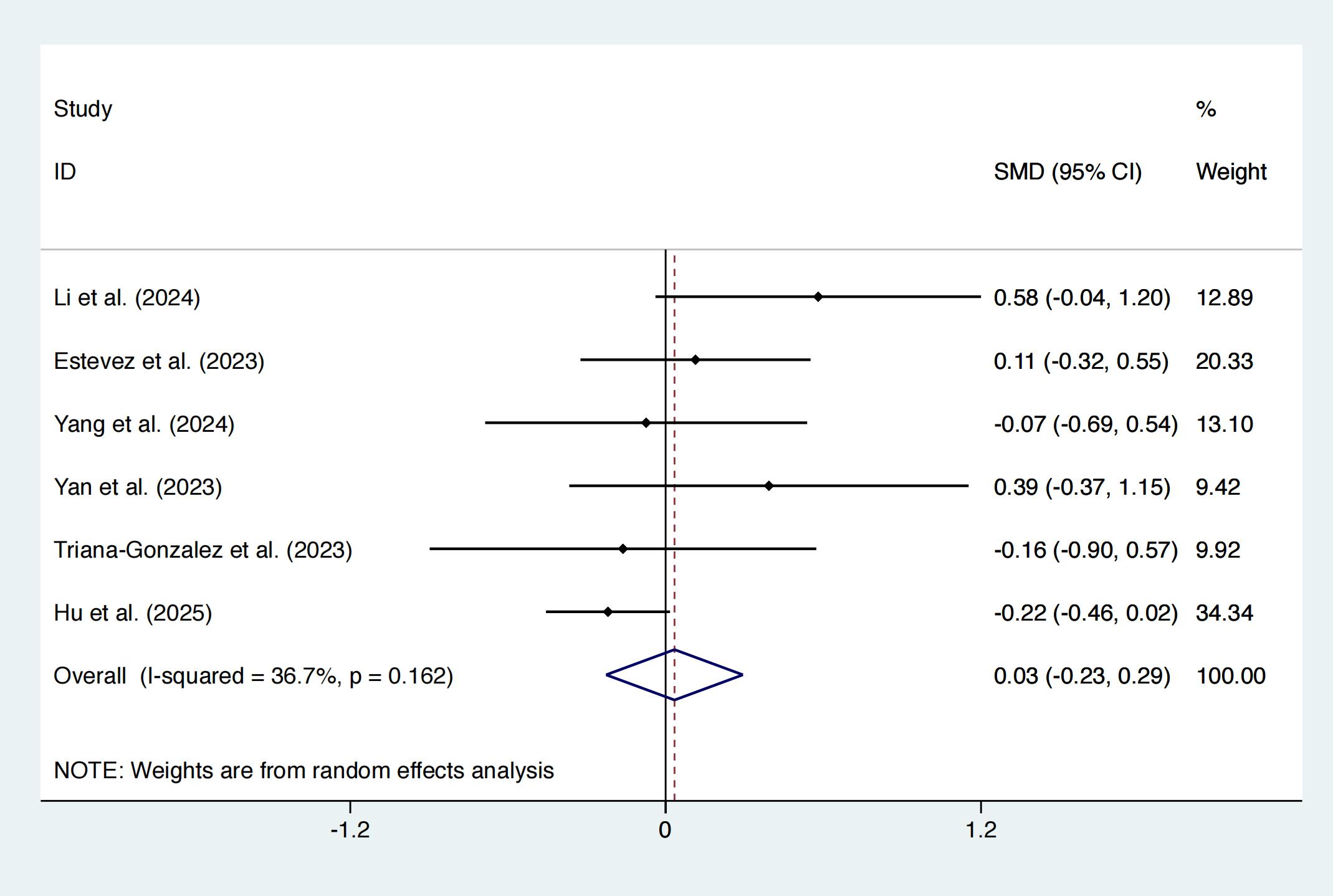
**

**Figure S29: Forest plot of differences in the laboratory findings between the HIV-positive group and the HIV-negative group: neutrophil count.**

**
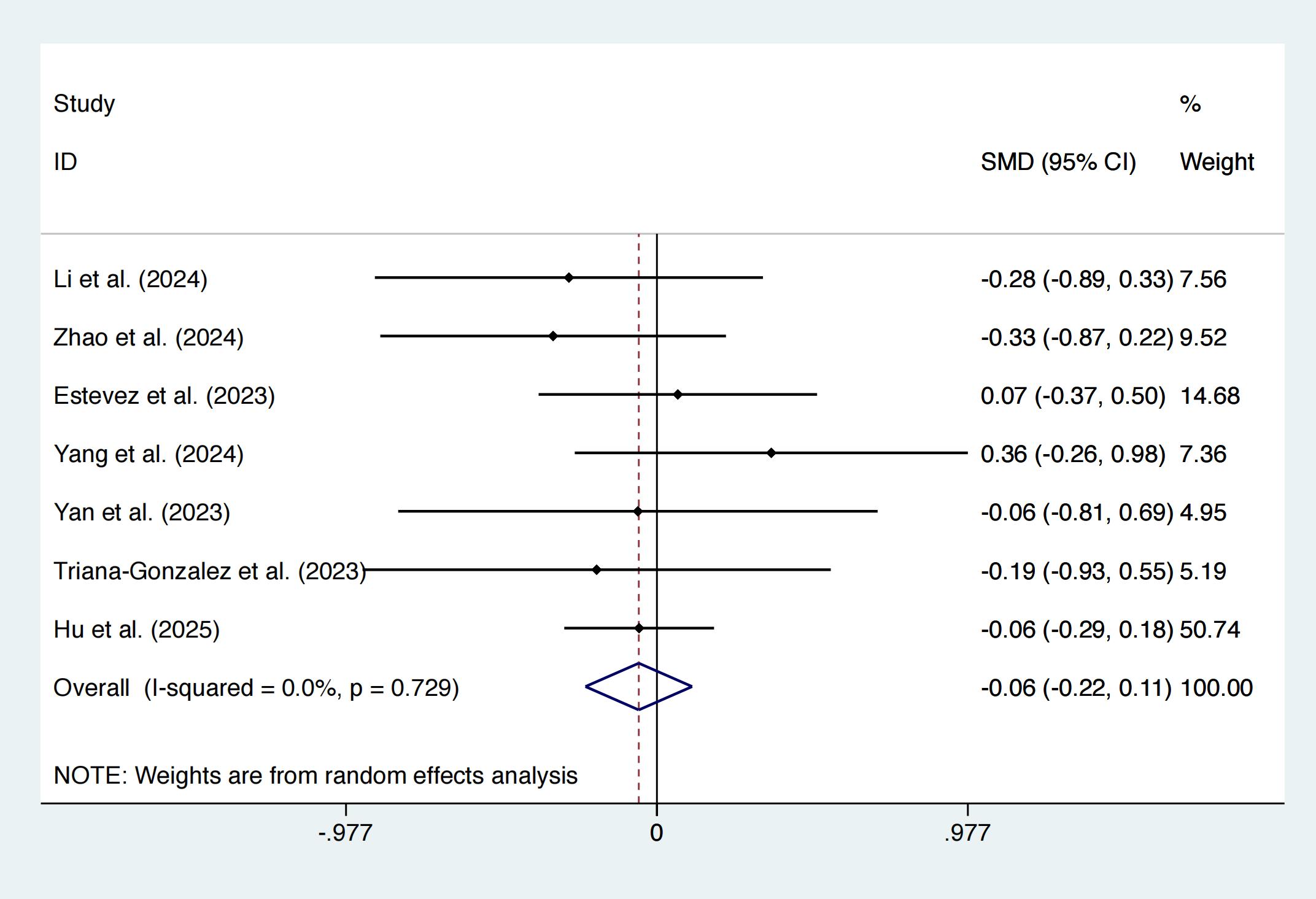
**

**Figure S30: Forest plot of differences in the laboratory findings between the HIV-positive group and the HIV-negative group: lymphocyte count.**

**
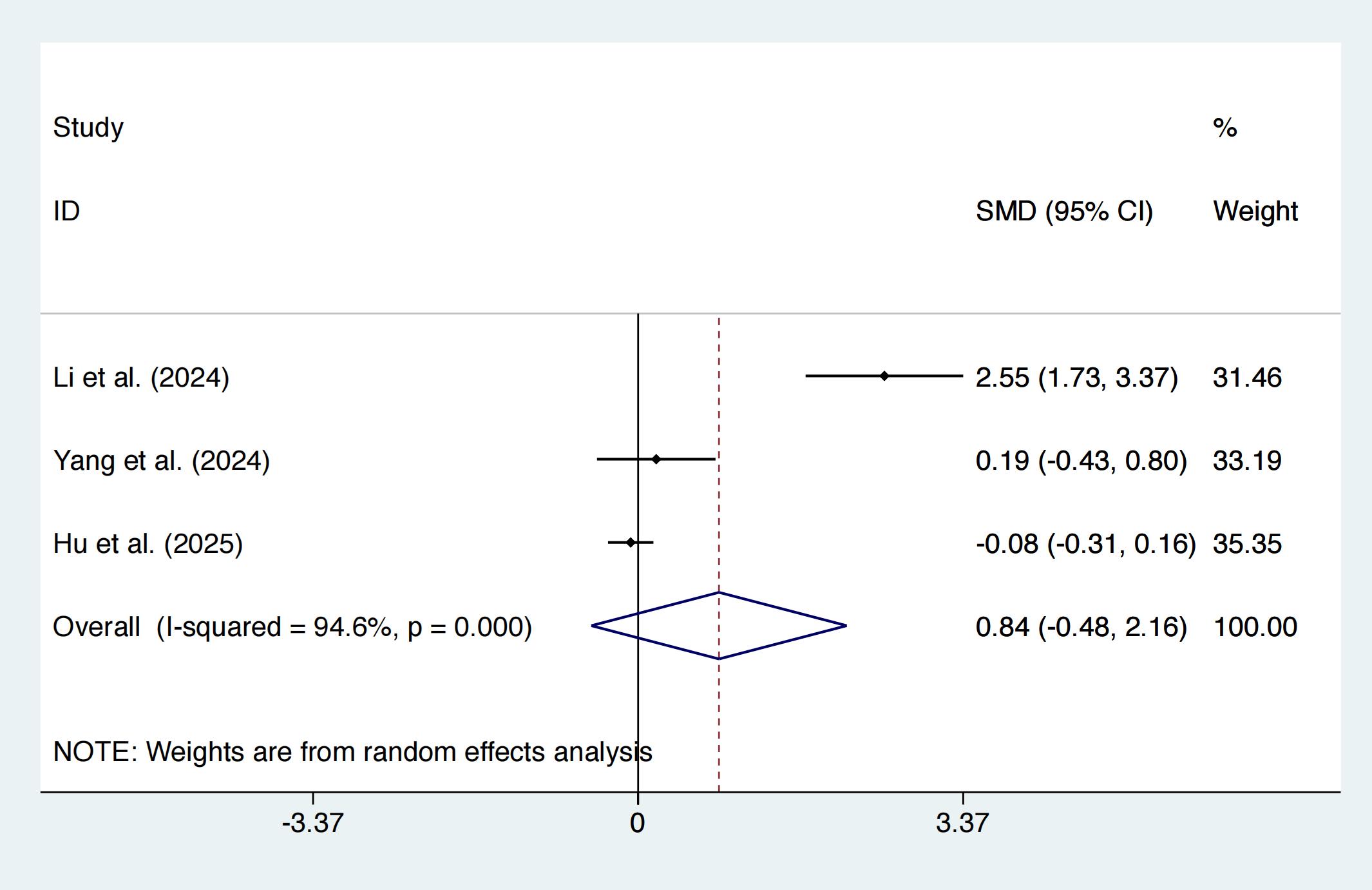
**

**Figure S31: Forest plot of differences in the laboratory findings between the HIV-positive group and the HIV-negative group: monocyte count.**

**
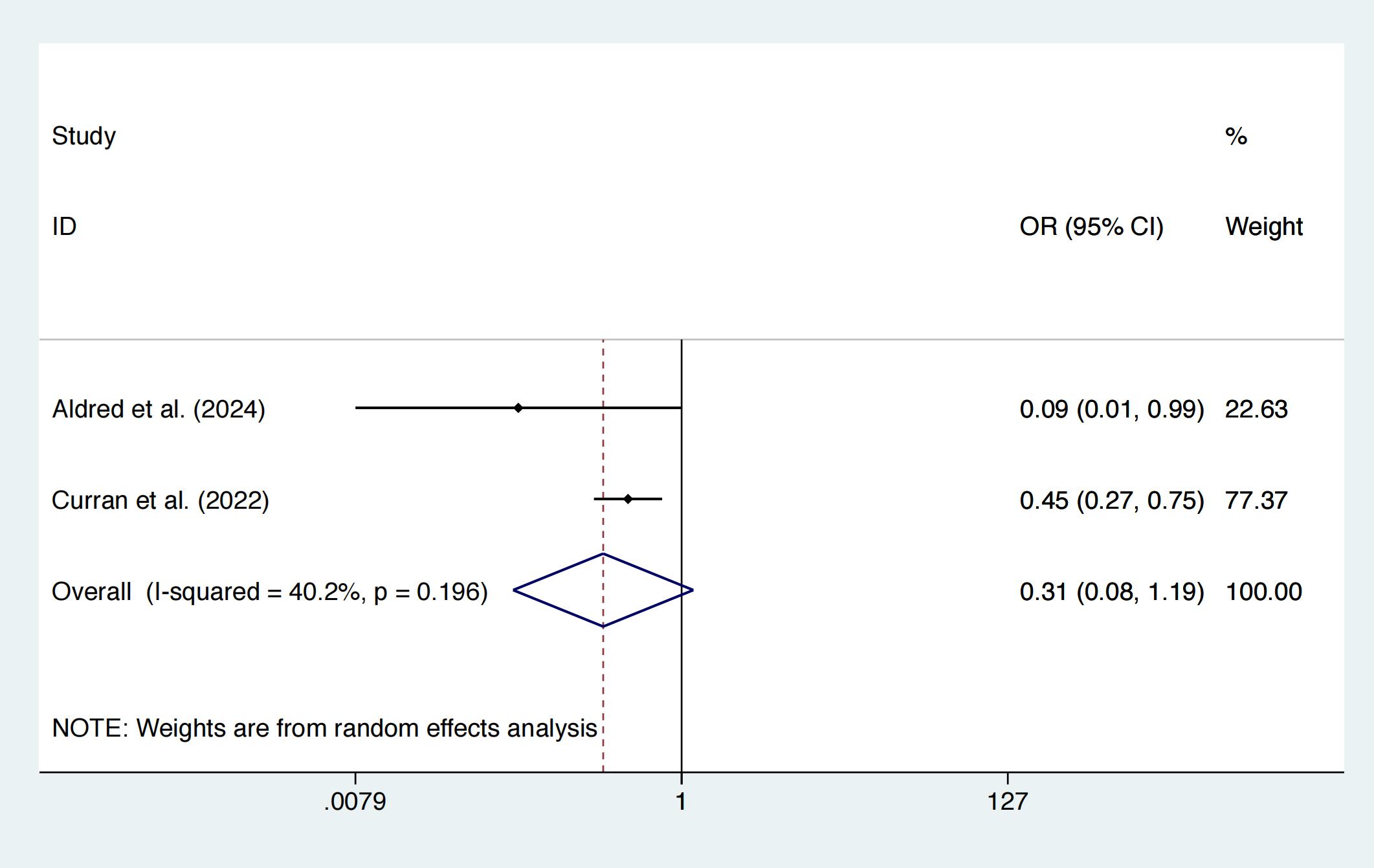
**

**Figure S32: Forest plot of differences in the laboratory findings between the HIV-positive group and the HIV-negative group: platelet count.**

**
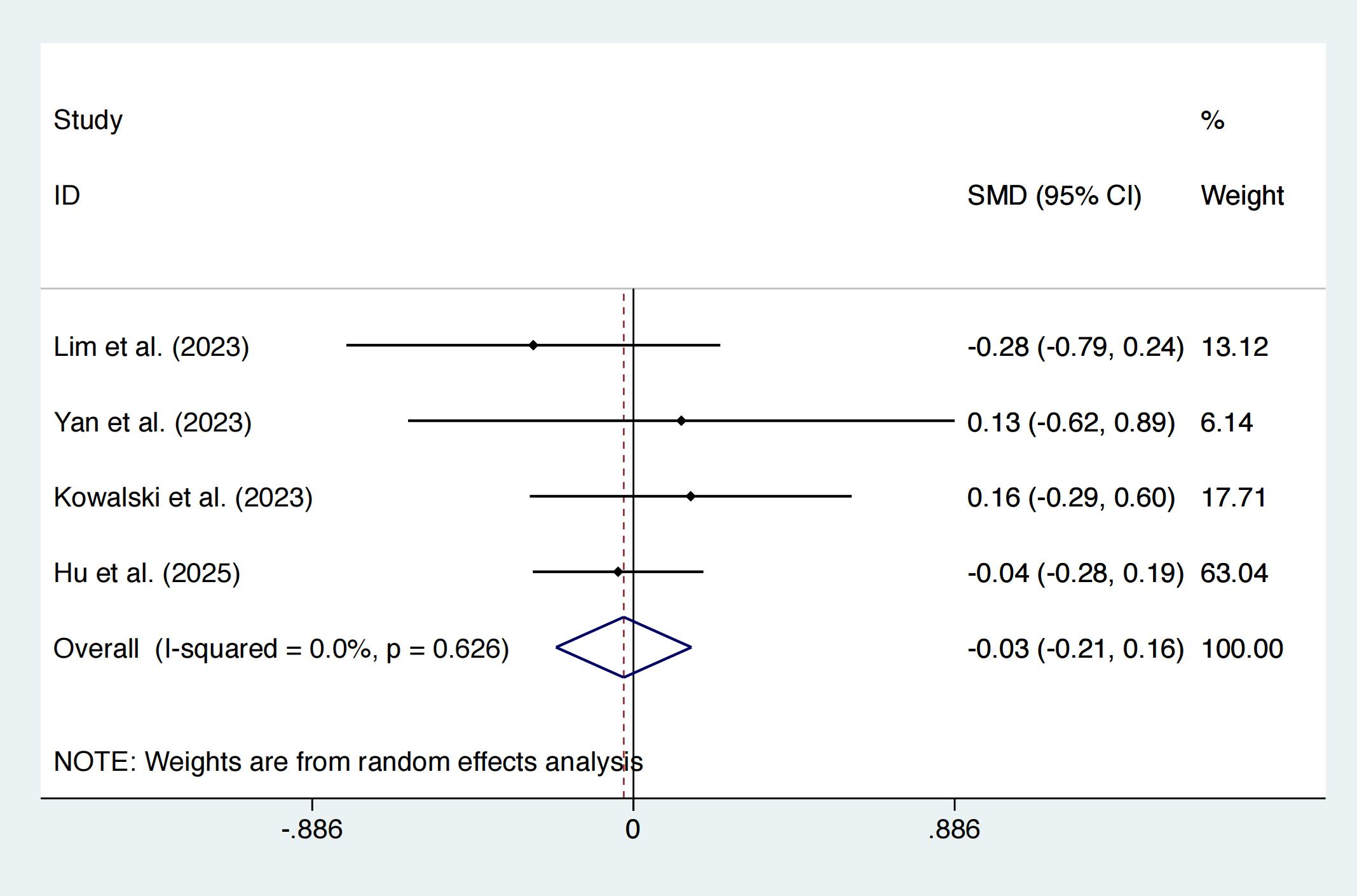
**

**Figure S33: Forest plot of differences in the laboratory findings between the HIV-positive group and the HIV-negative group: alanine transaminase.**

**
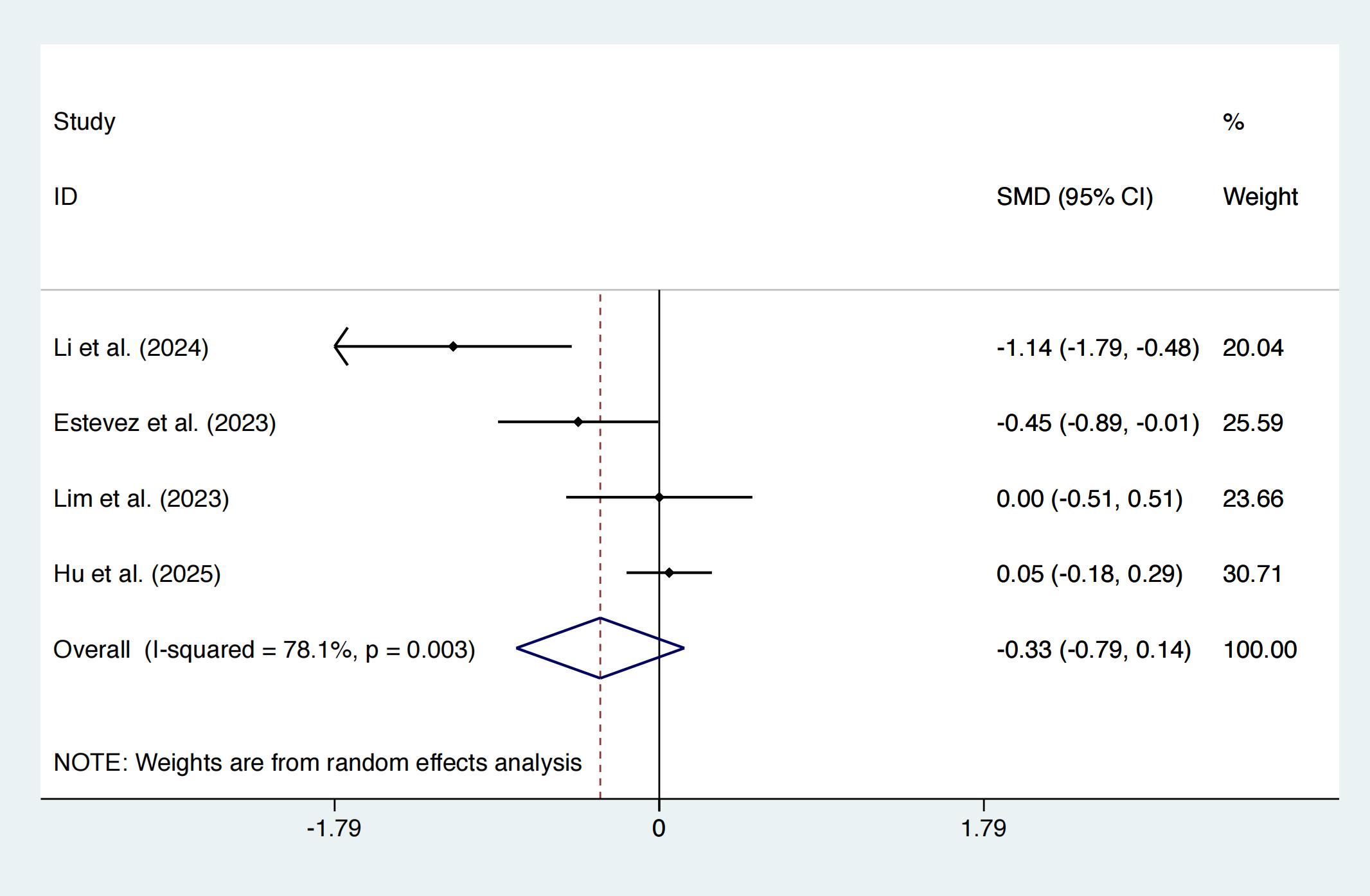
**

**Figure S34: Forest plot of differences in the laboratory findings between the HIV-positive group and the HIV-negative group: total bilirubin .**

**
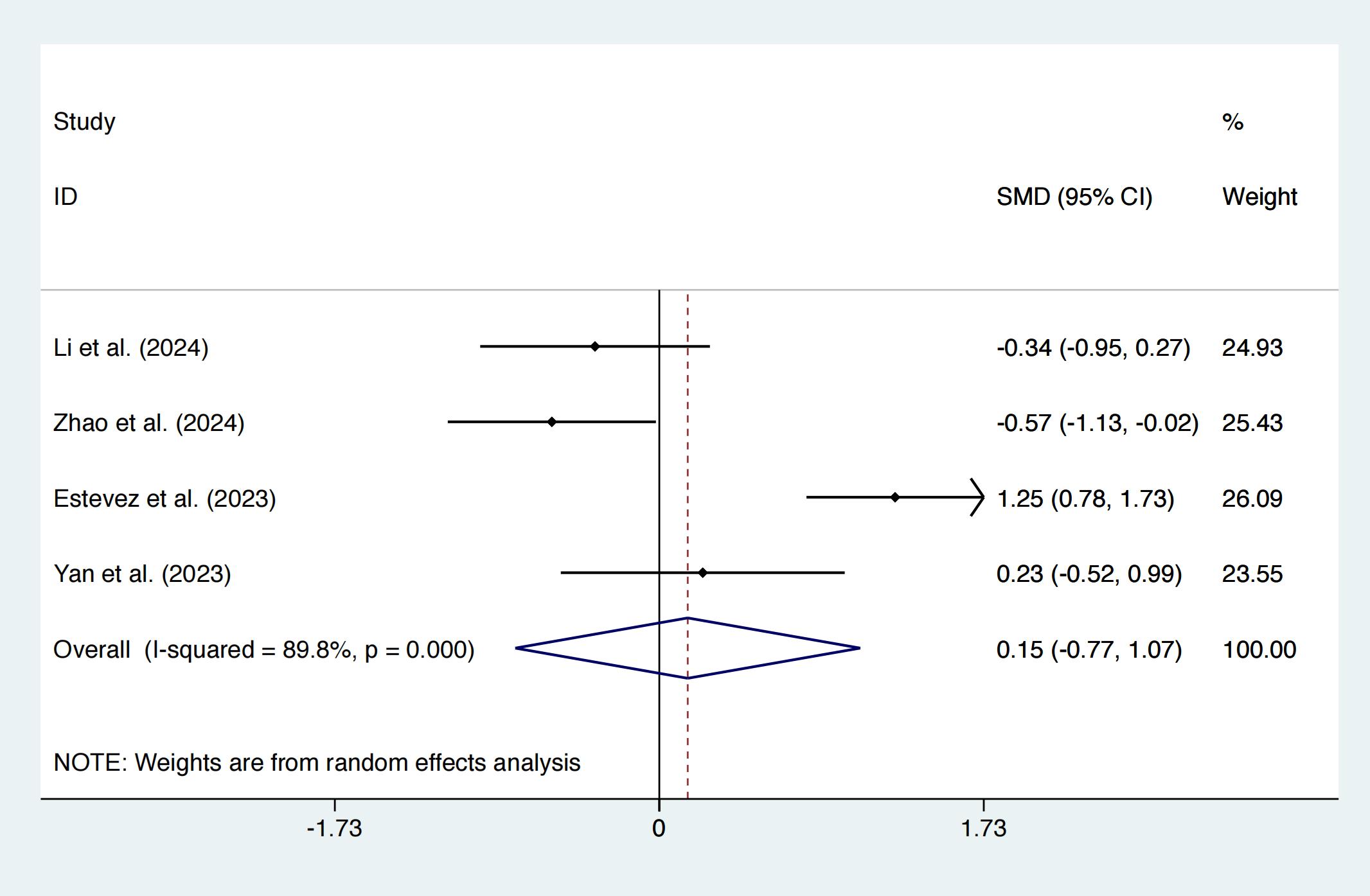
**

**Figure S35: Forest plot of differences in the laboratory findings between the HIV-positive group and the HIV-negative group: lactate dehydrogenase.**

**
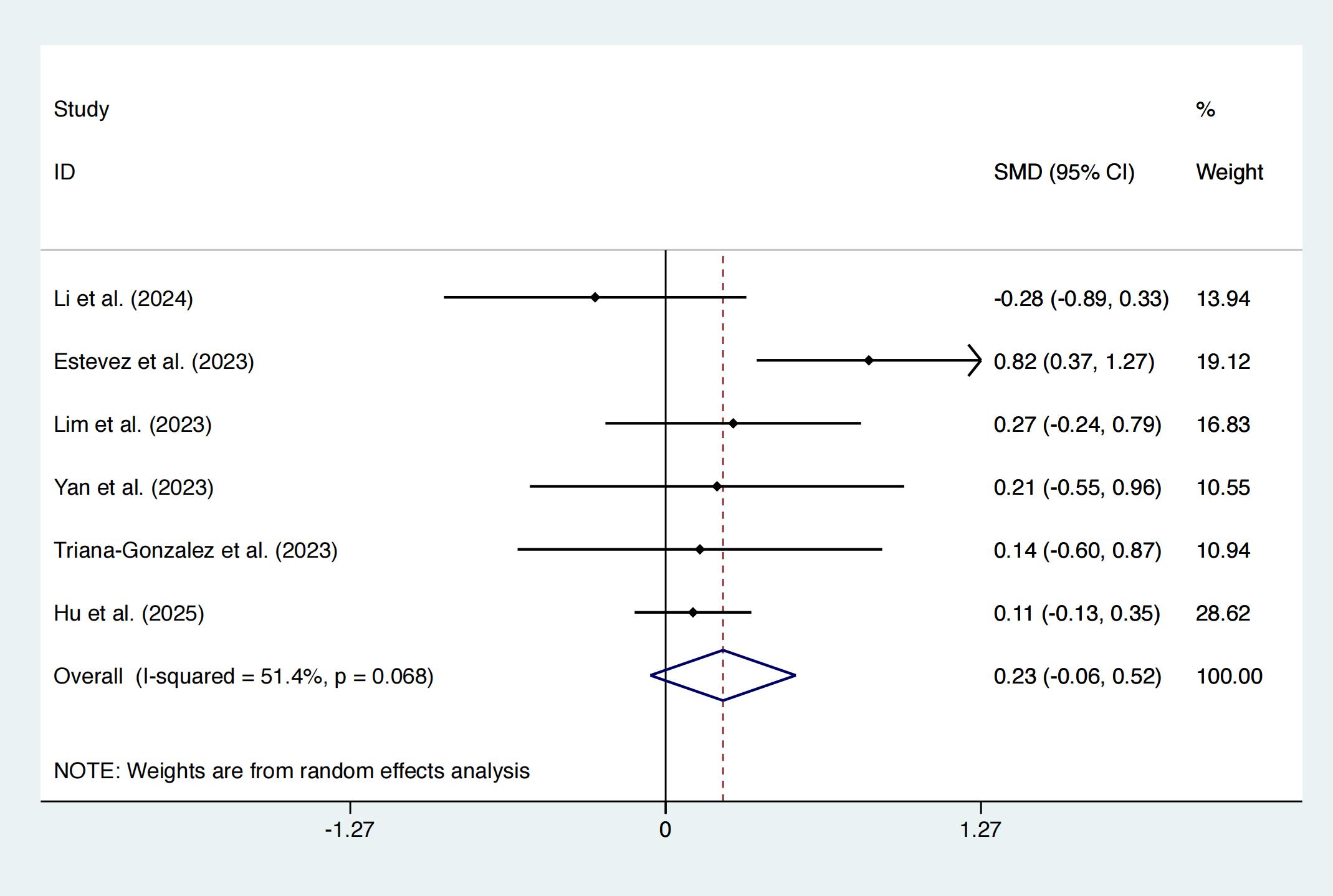
**

**Figure S36: Forest plot of differences in the laboratory findings between the HIV-positive group and the HIV-negative group: creatinine.**

**
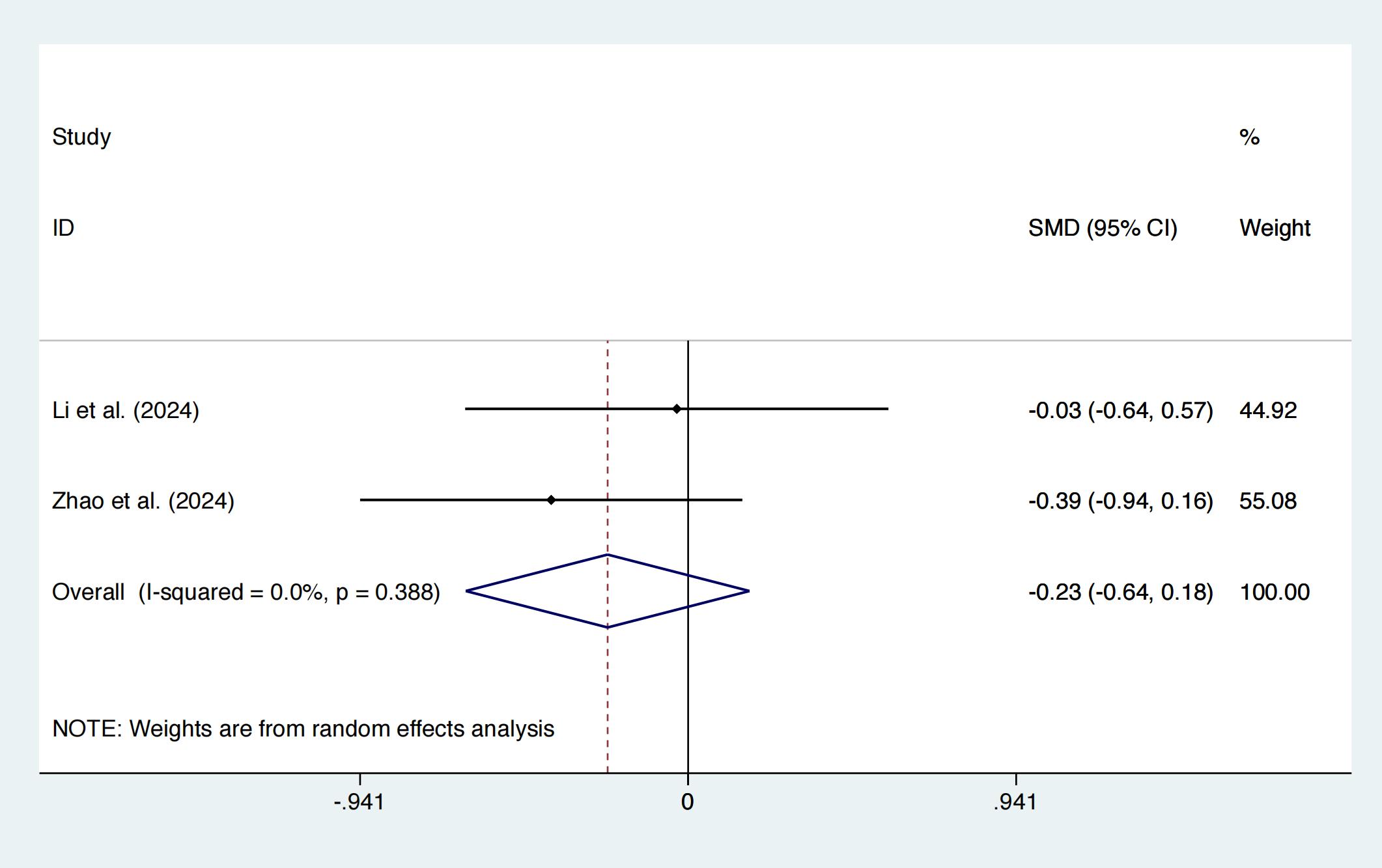
**

**Figure S37: Forest plot of differences in the laboratory findings between the HIV-positive group and the HIV-negative group: creatine kinase.**

**
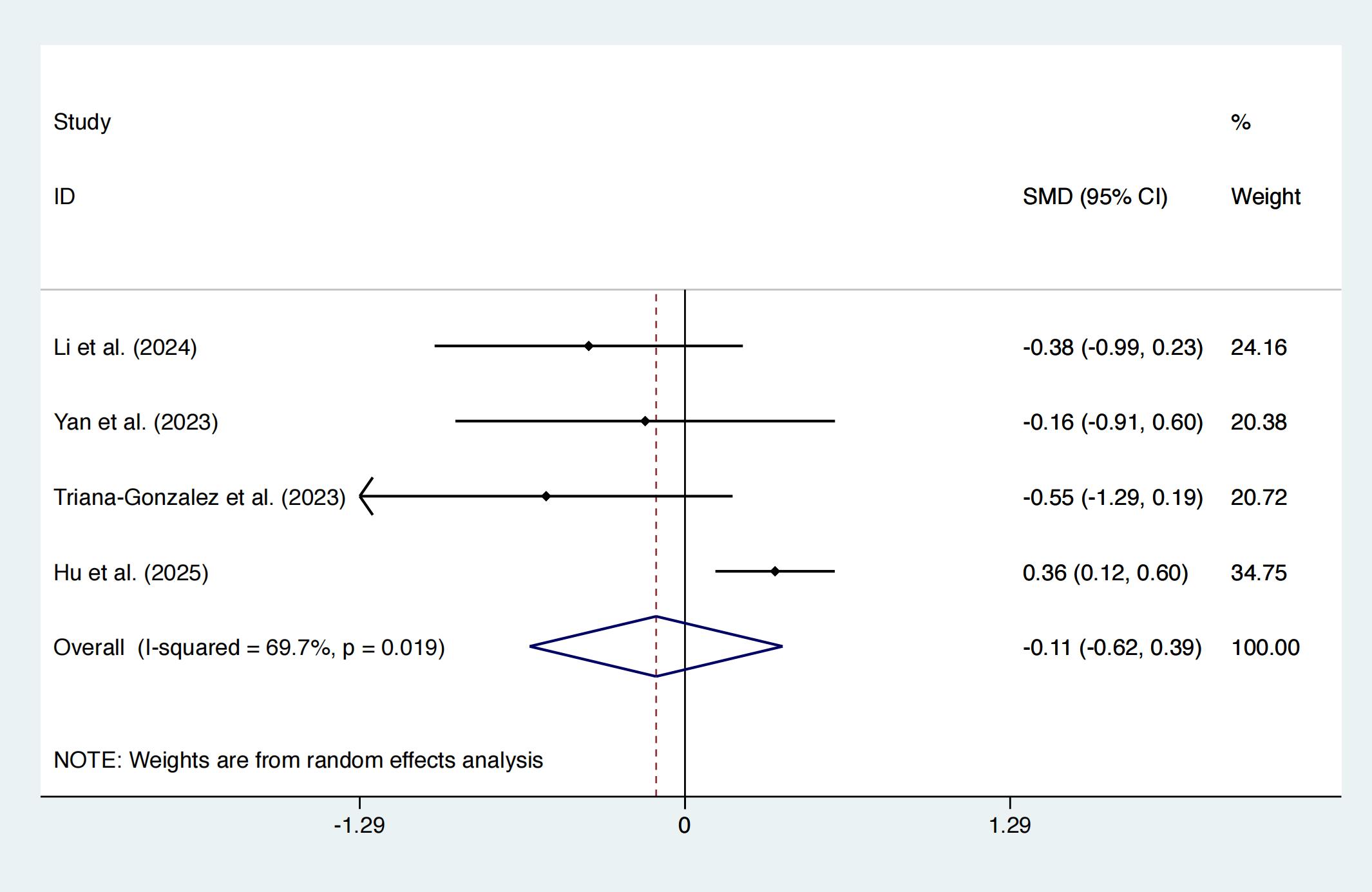
**

**Figure S38: Forest plot of differences in the laboratory findings between the HIV-positive group and the HIV-negative group: procalcitonin.**

**
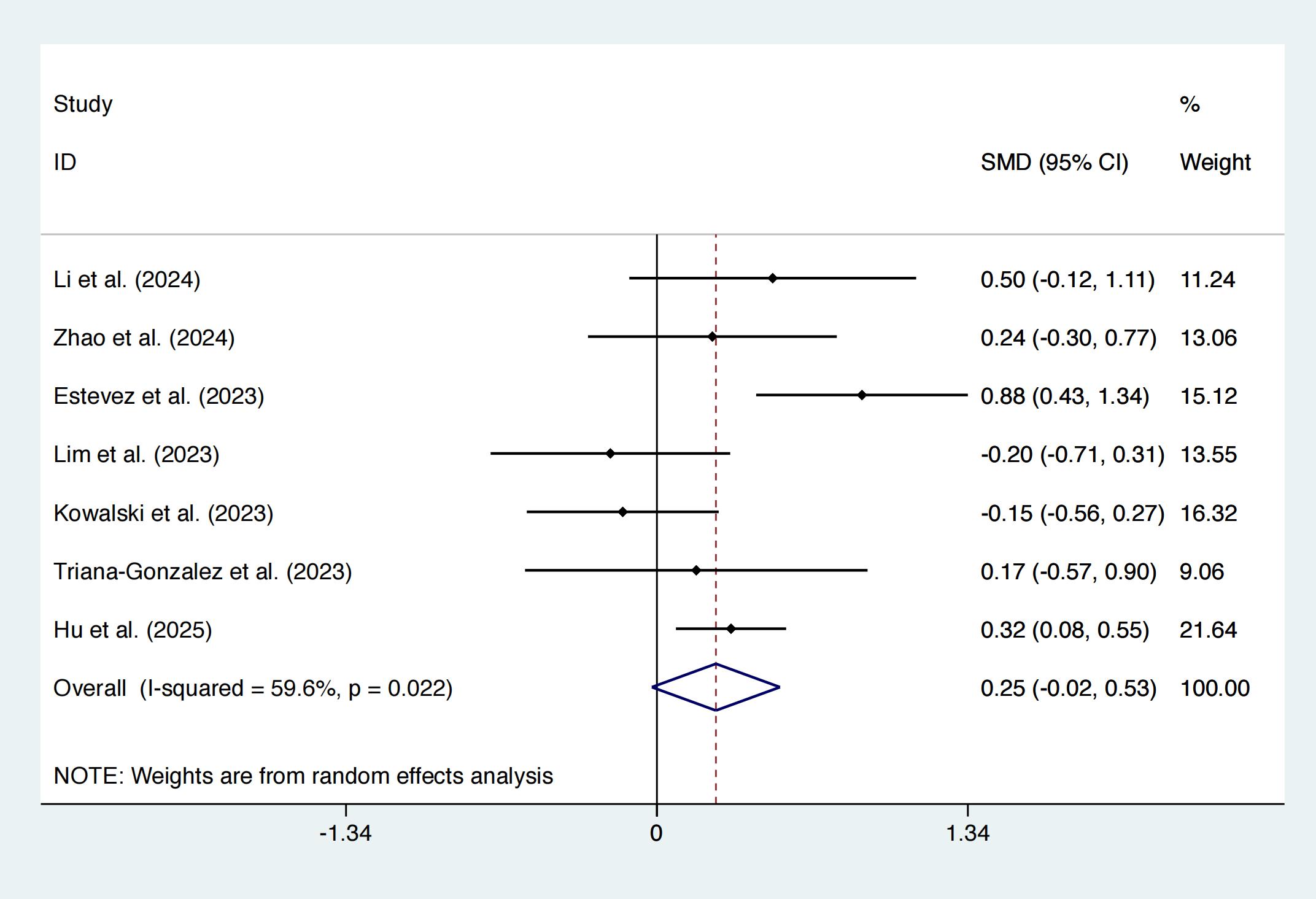
**

**Figure S39: Forest plot of differences in the laboratory findings between the HIV-positive group and the HIV-negative group: C-reactive protein.**

**
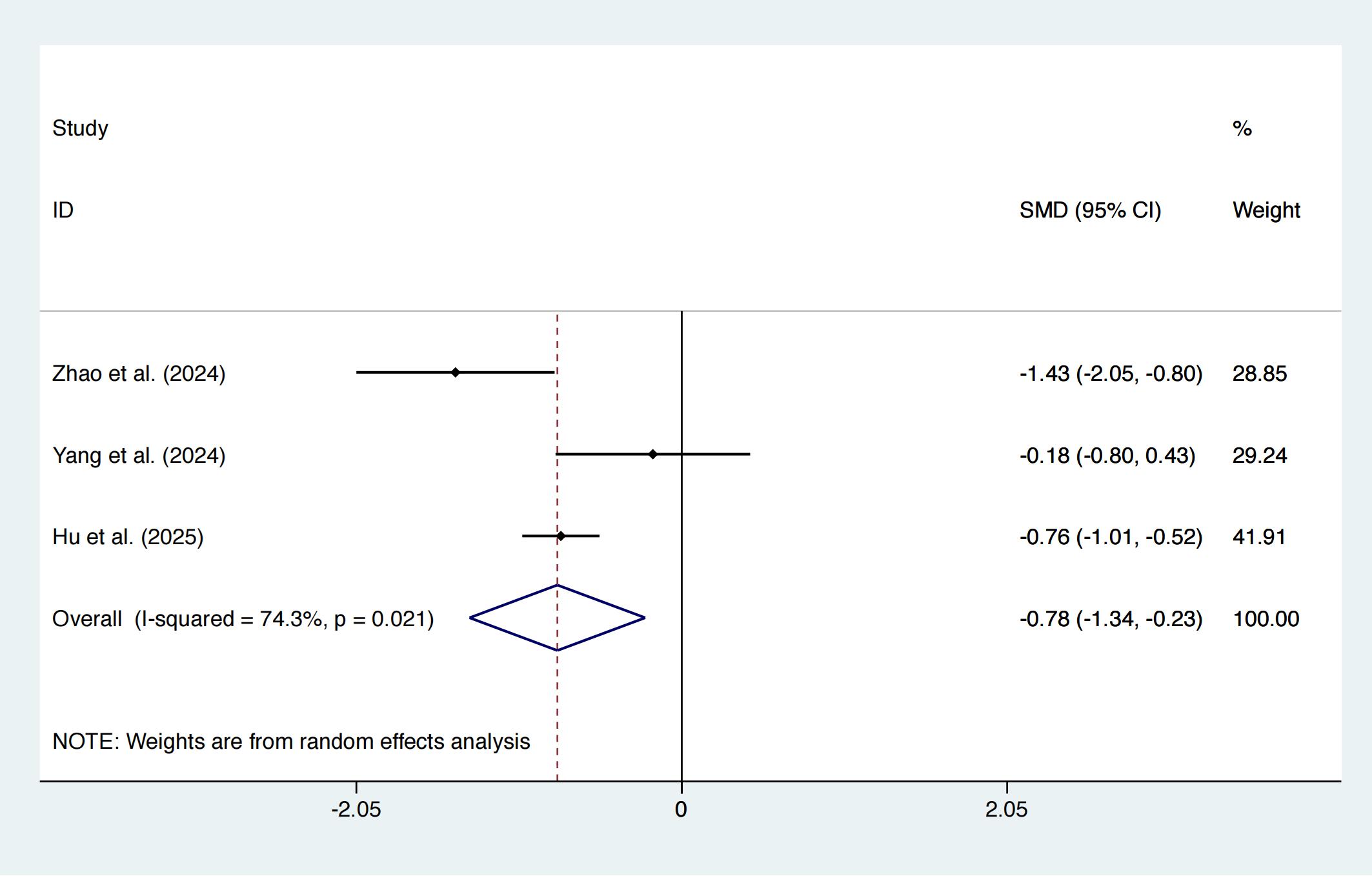
**

**Figure S40: Forest plot of differences in the laboratory findings between the HIV-positive group and the HIV-negative group: CD4^+^ T-cell count.**

**
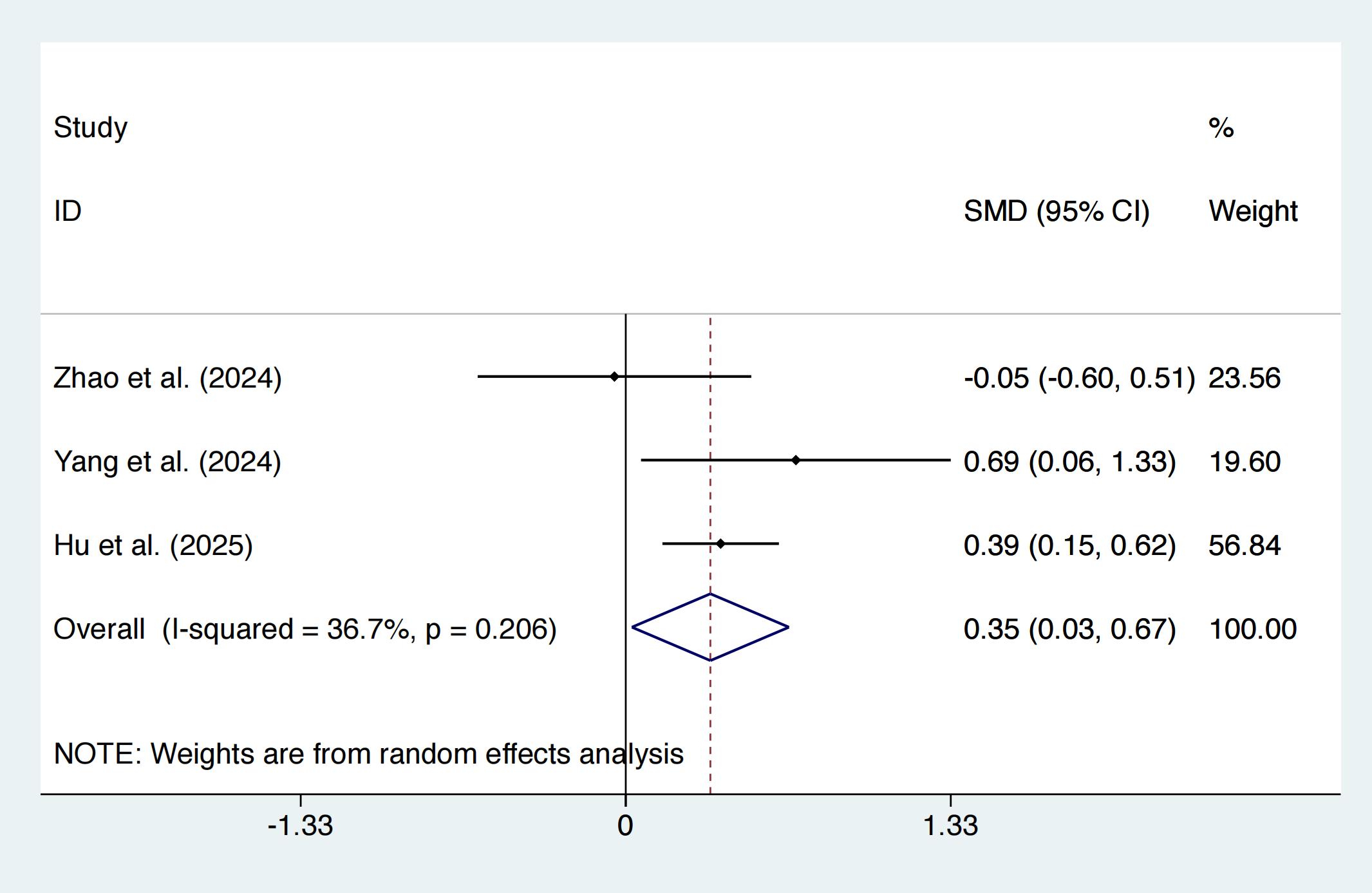
**

**Figure S41: Forest plot of differences in the laboratory findings between the HIV-positive group and the HIV-negative group: CD8^+^ T-cell count.**

**Figure S42: Forest plot of differences in the number of lesions between the HIV-positive group and the HIV-negative group: more than 10.**

**
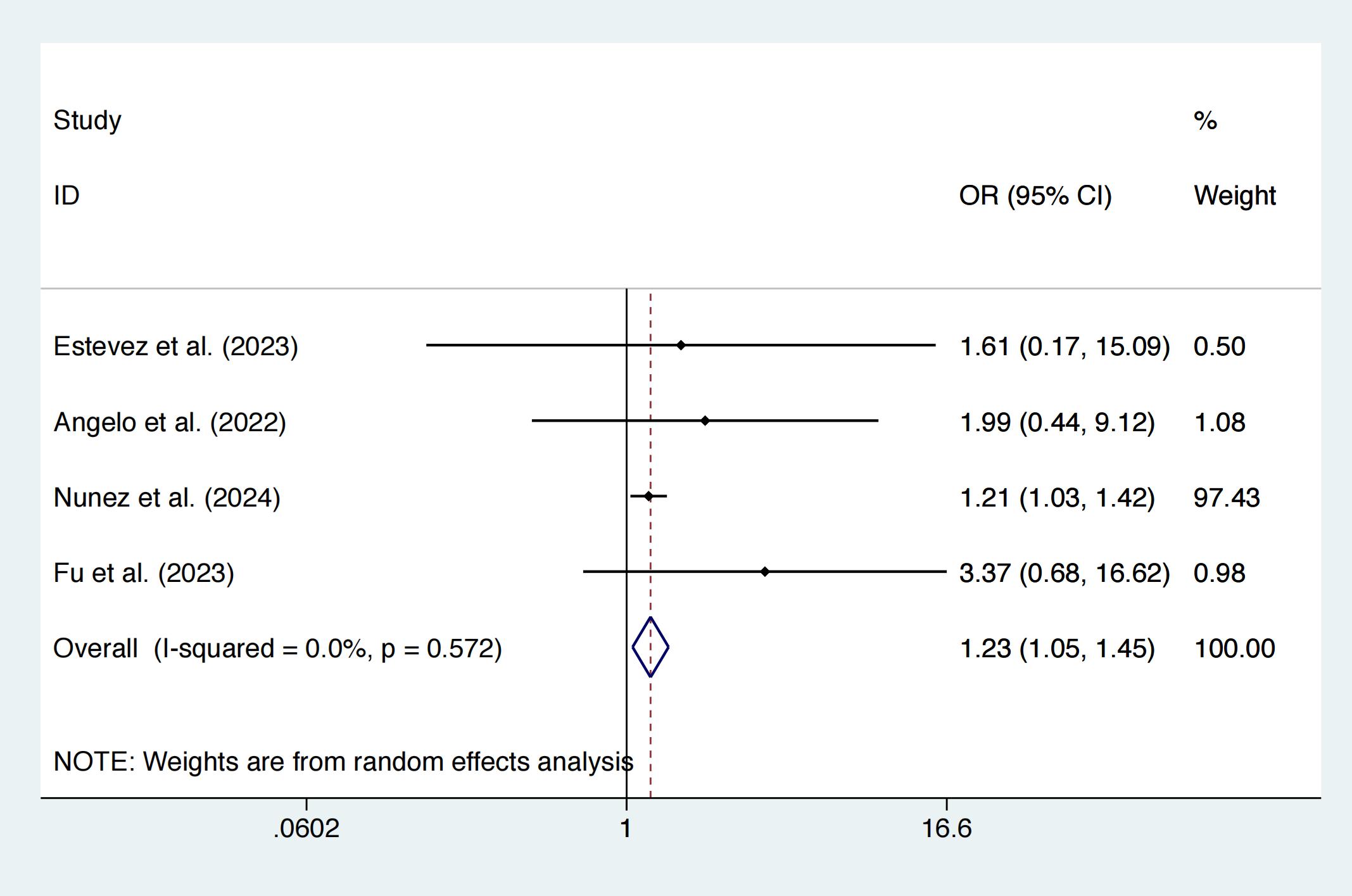
**

**Figure S43: Forest plot of differences in the location of lesions between the HIV-positive group and the HIV-negative group: neck.**

**
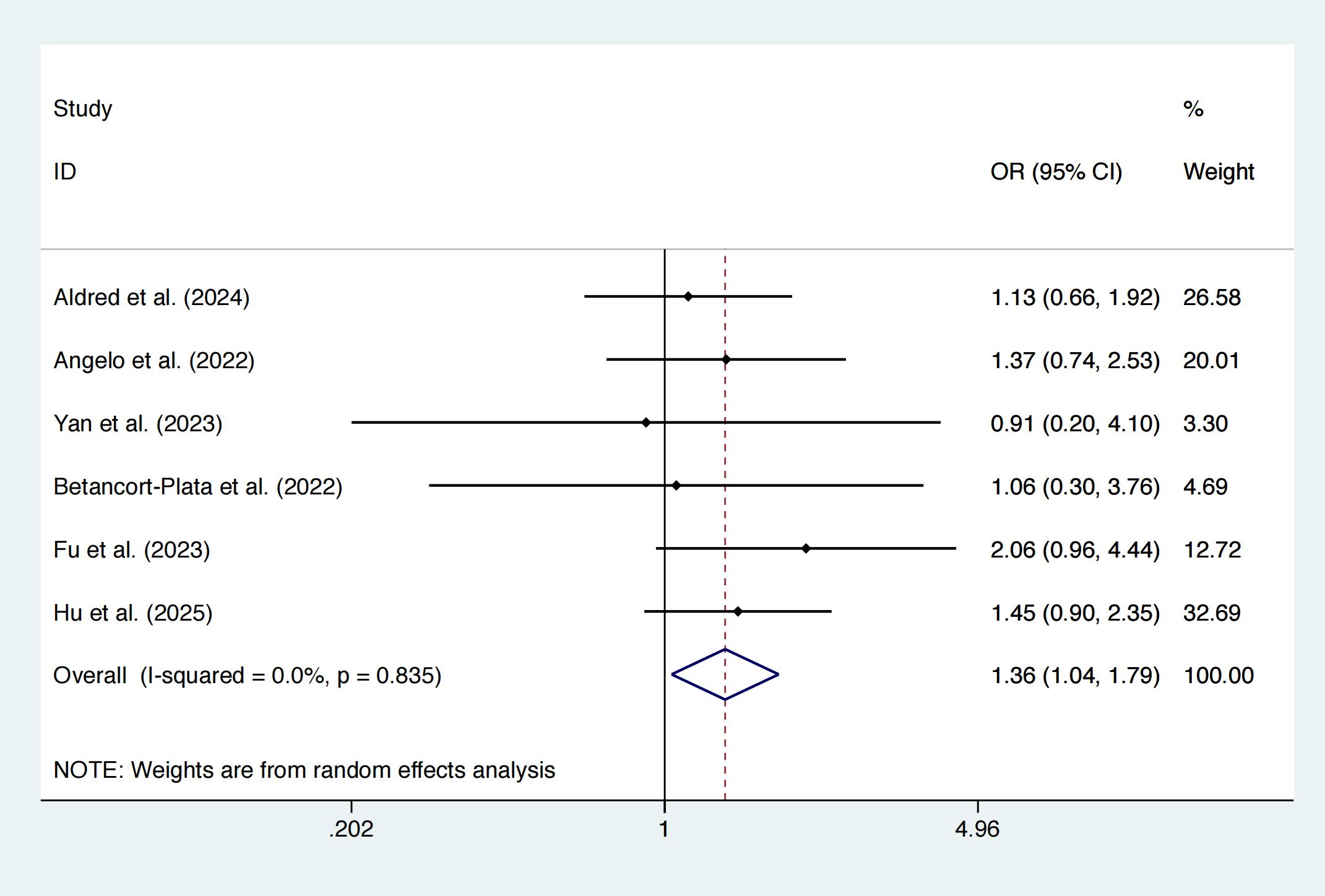
**

**Figure S44: Forest plot of differences in the location of lesions between the HIV-positive group and the HIV-negative group: trunk.**

**Figure S45: Forest plot of differences in the location of lesions between the HIV-positive group and the HIV-negative group: anus.**

**
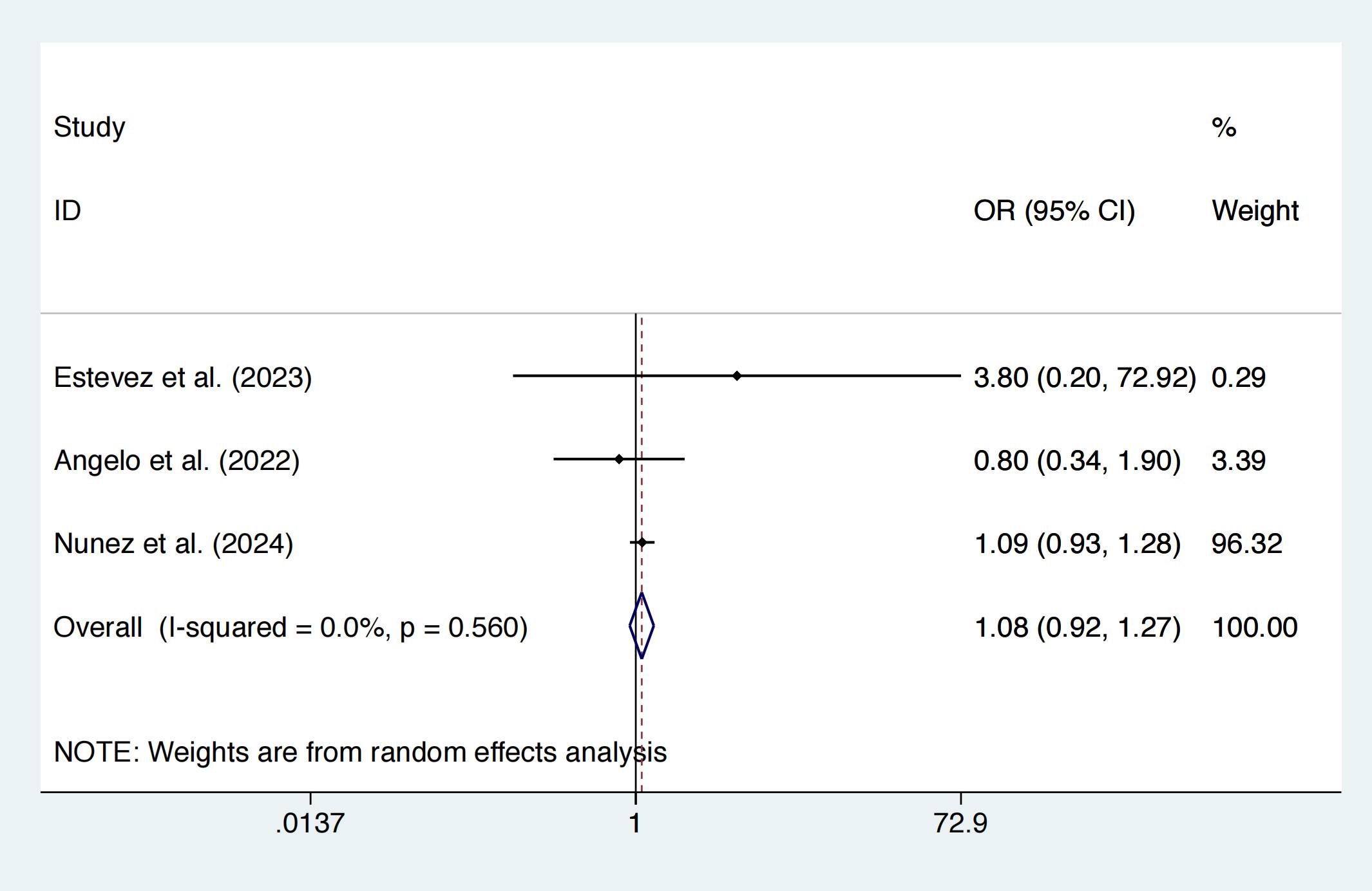
**

**Figure S46: Forest plot of differences in the location of lesions between the HIV-positive group and the HIV-negative group: palms.**

**
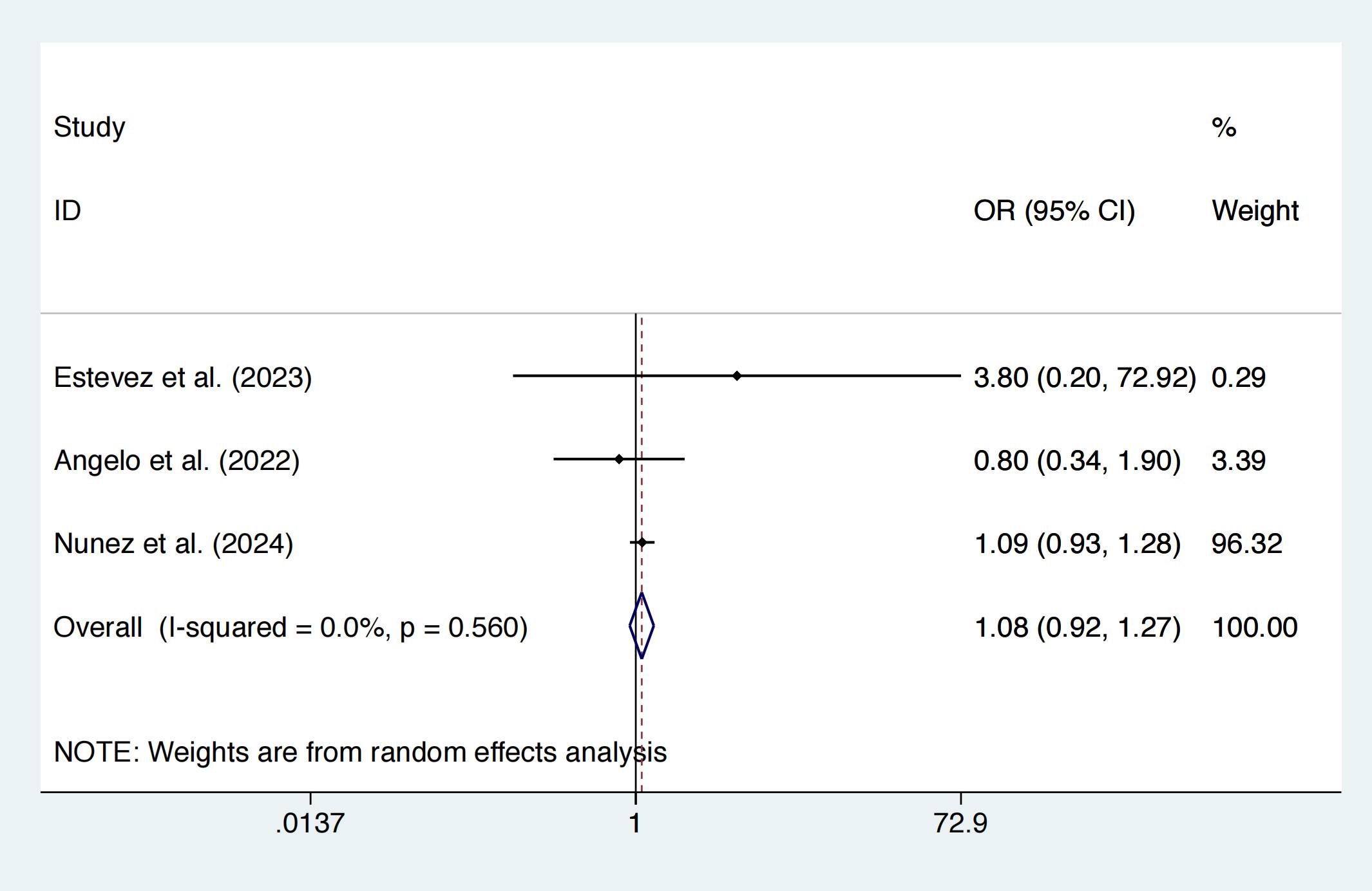
**

**Figure S47: Forest plot of differences in the location of lesions between the HIV-positive group and the HIV-negative group: soles.**

**
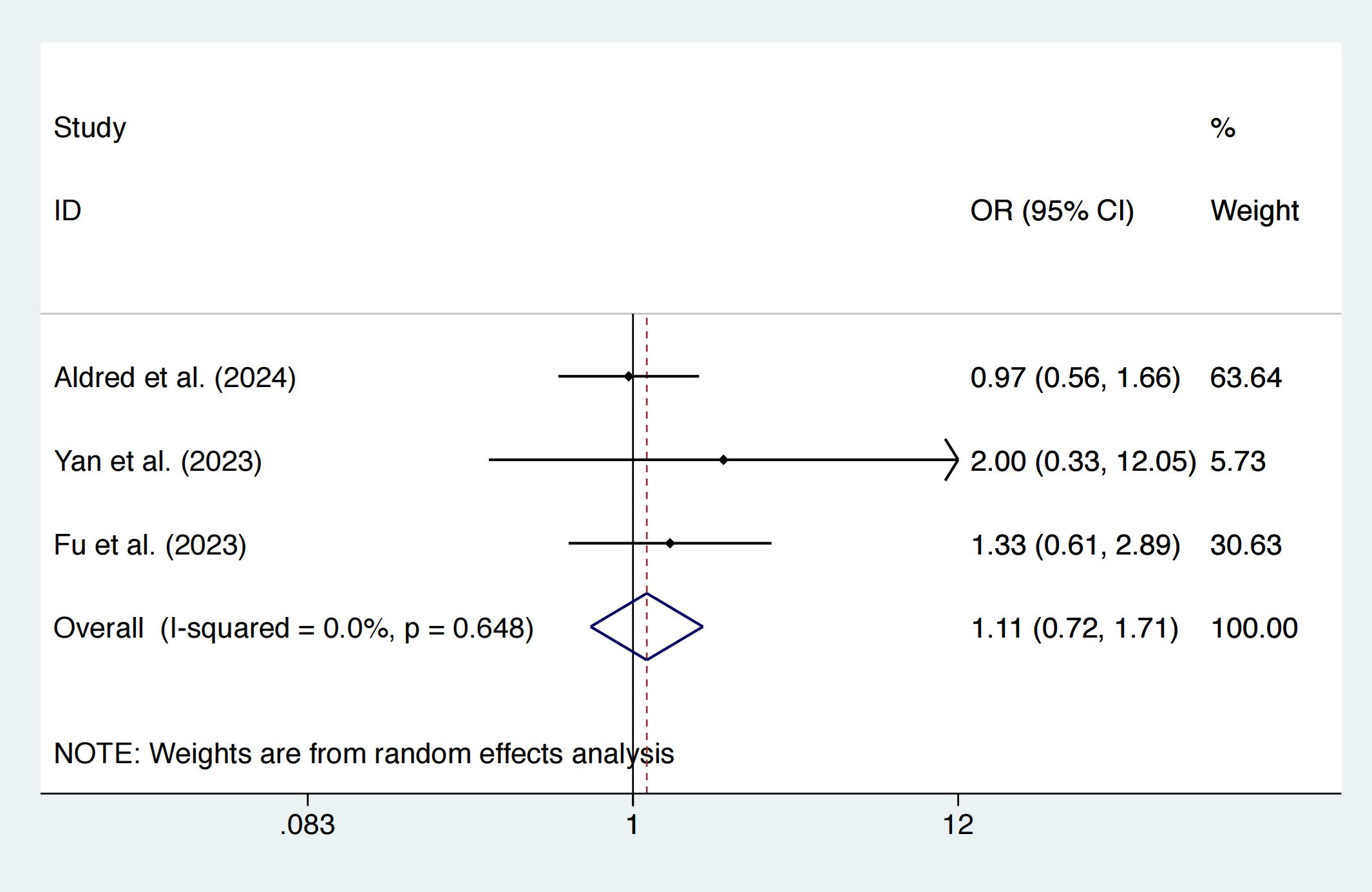
**

**Figure S48: Forest plot of differences in the location of lesions between the HIV-positive group and the HIV-negative group: face/head.**

**
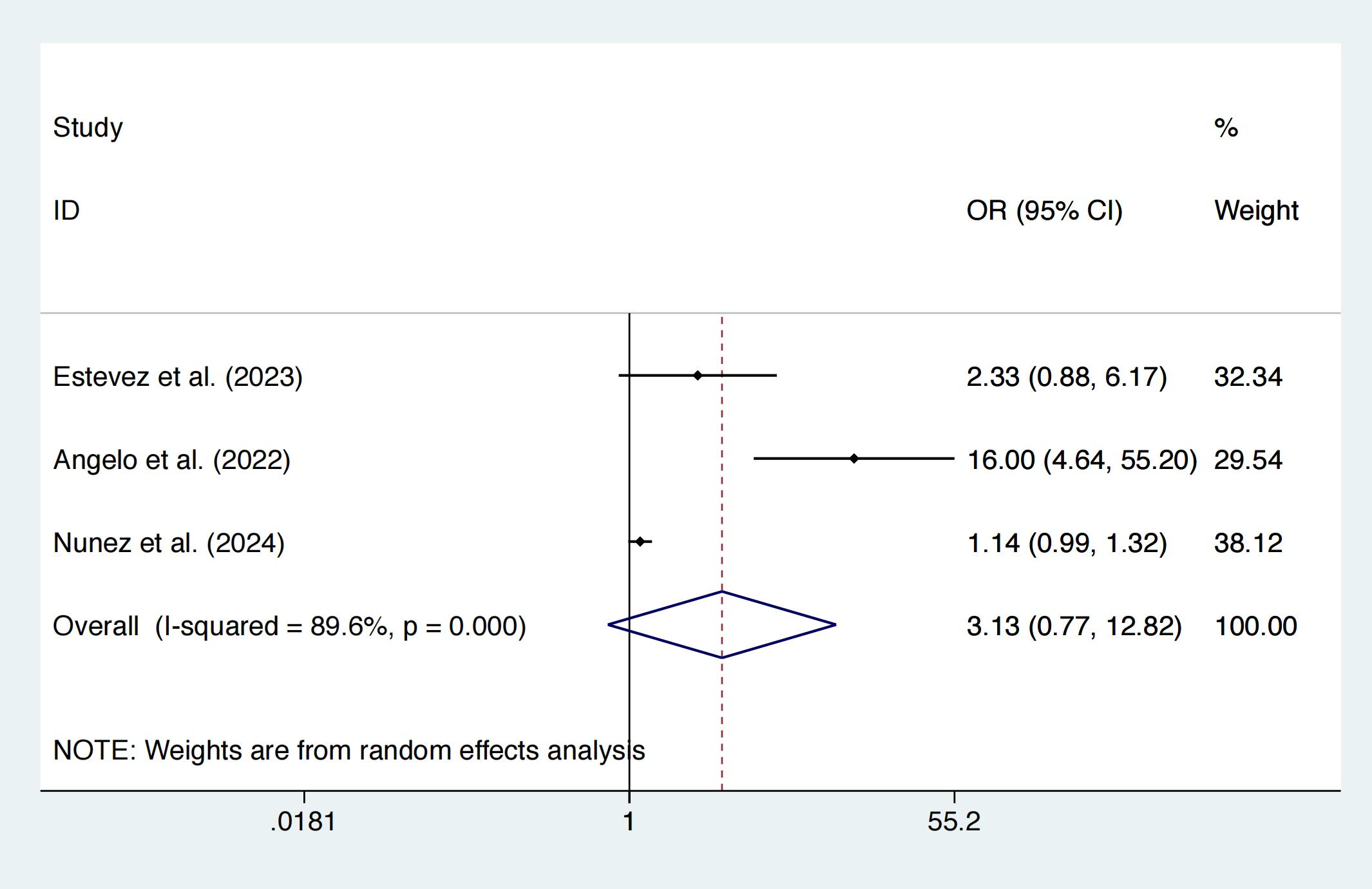
**

**Figure S49: Forest plot of differences in the location of lesions between the HIV-positive group and the HIV-negative group: arms.**

**
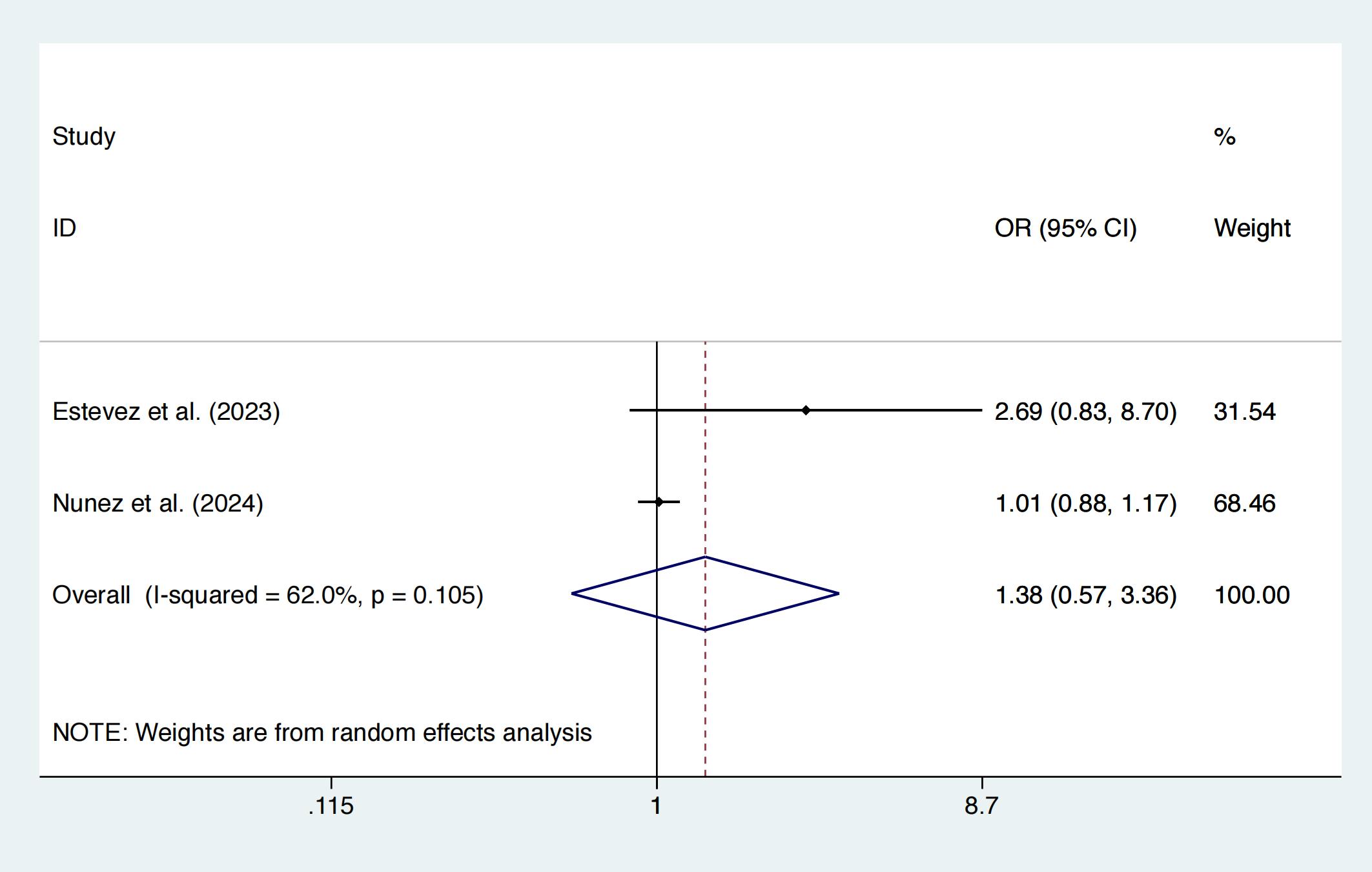
**

**Figure S50: Forest plot of differences in the location of lesions between the HIV-positive group and the HIV-negative group: legs.**

**
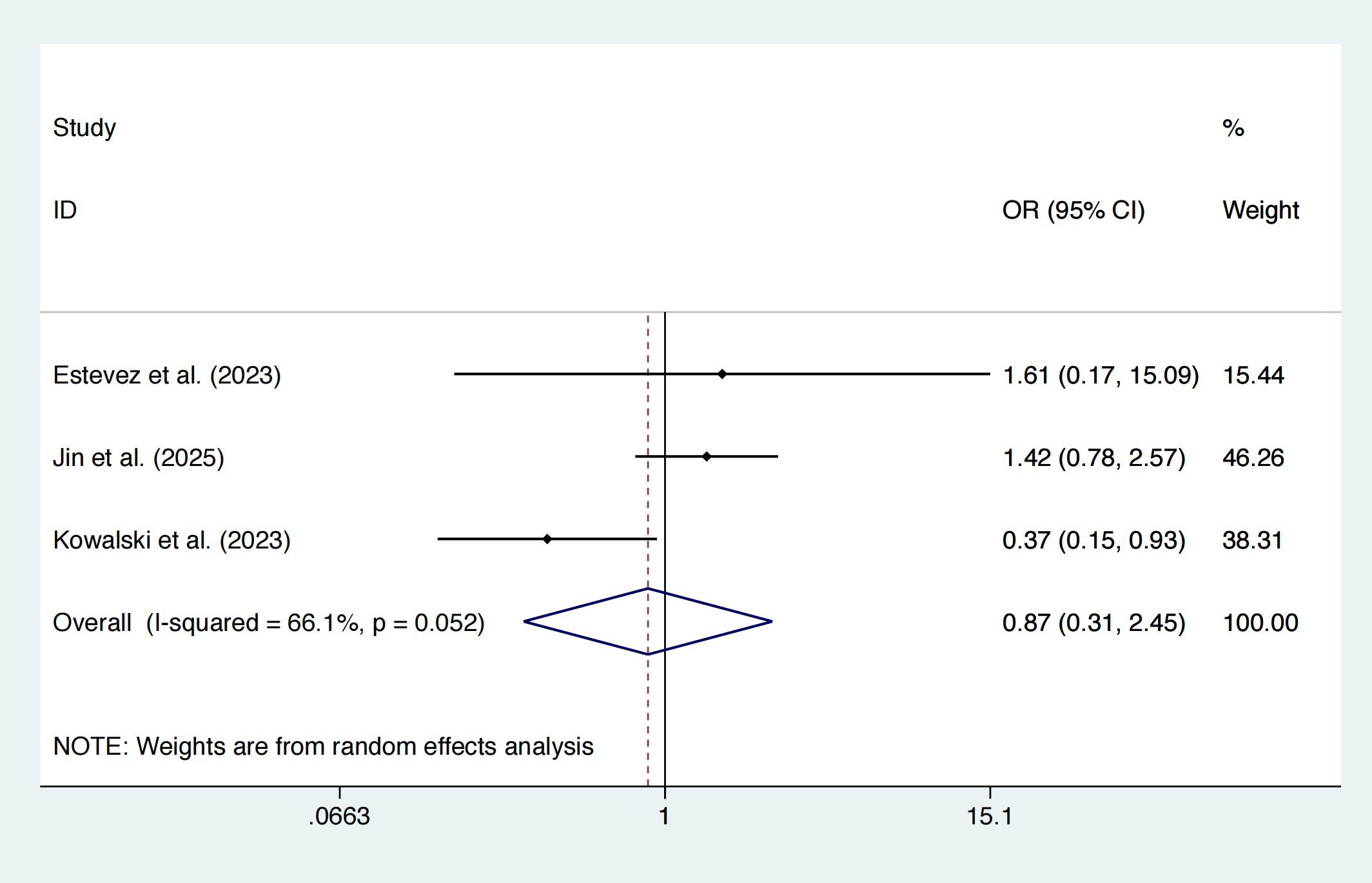
**

**Figure S51: Forest plot of differences in the location of lesions between the HIV-positive group and the HIV-negative group: oral cavity.**

**Figure S52: Forest plot of differences in the location of lesions between the HIV-positive group and the HIV-negative group: genitals .**

**
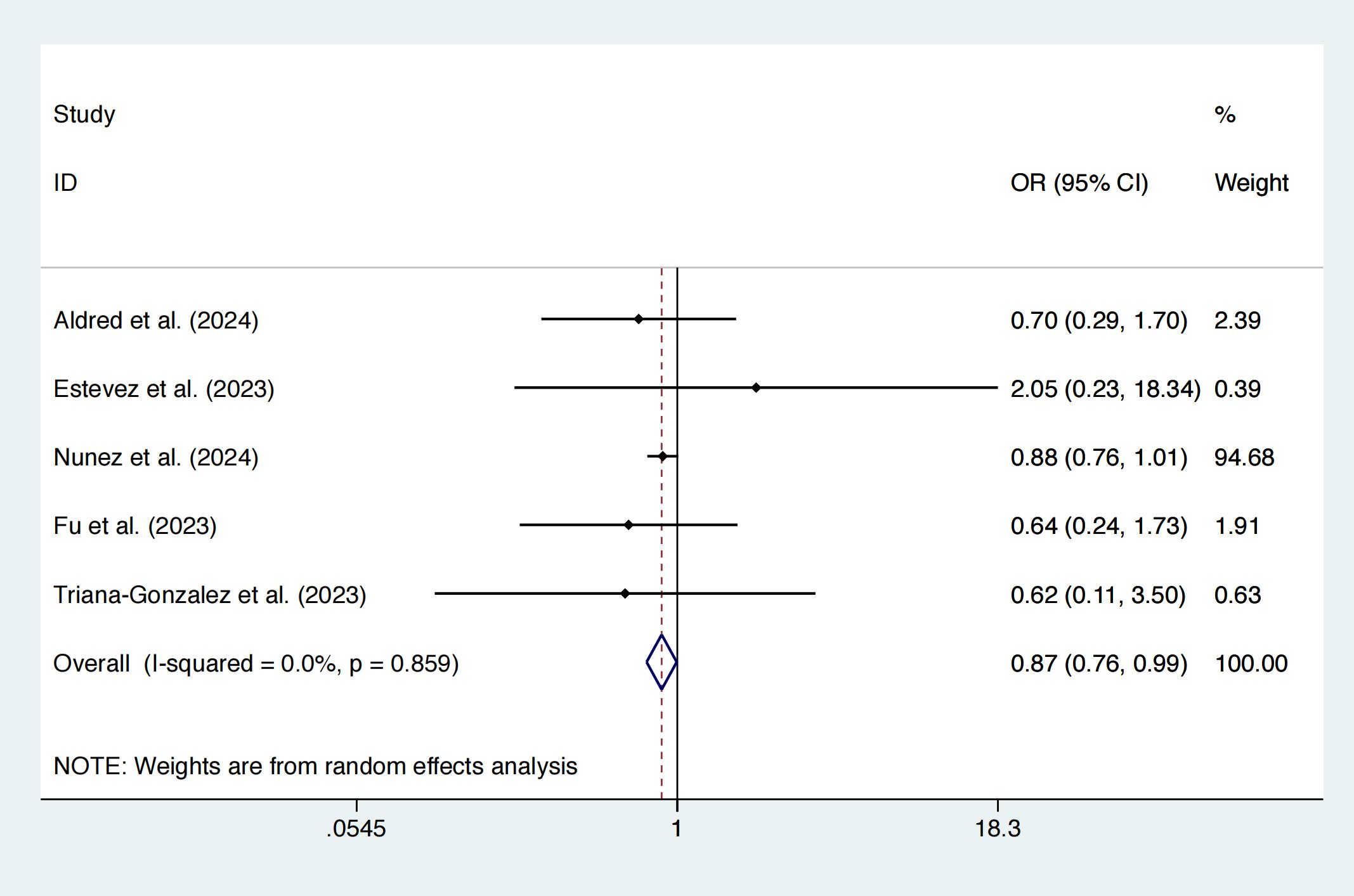
**

**Figure S53: Forest plot of differences in the type of lesions between the HIV-positive group and the HIV-negative group: maculae.**

**
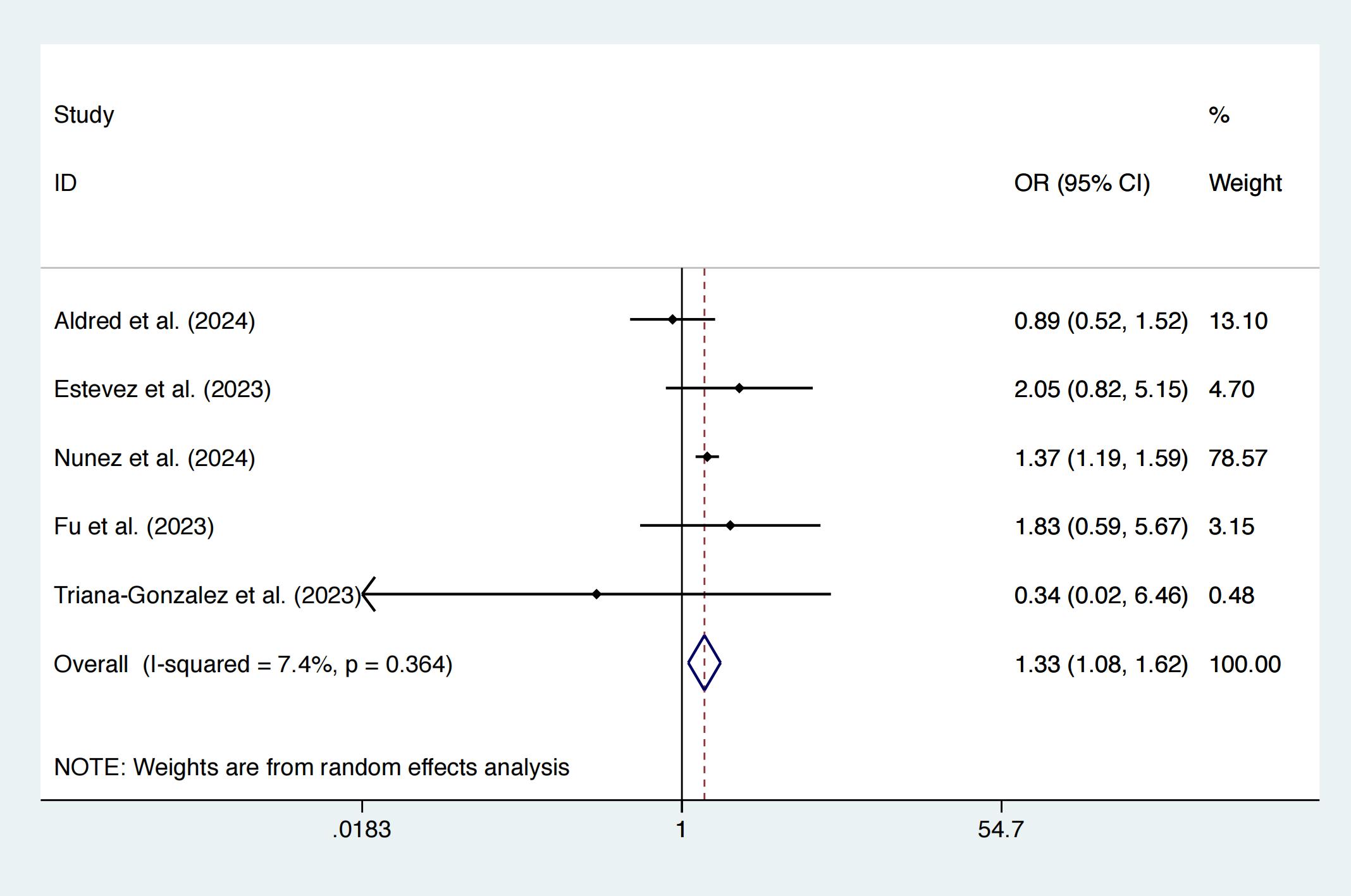
**

**Figure S54: Forest plot of differences in the type of lesions between the HIV-positive group and the HIV-negative group: pustules**

**
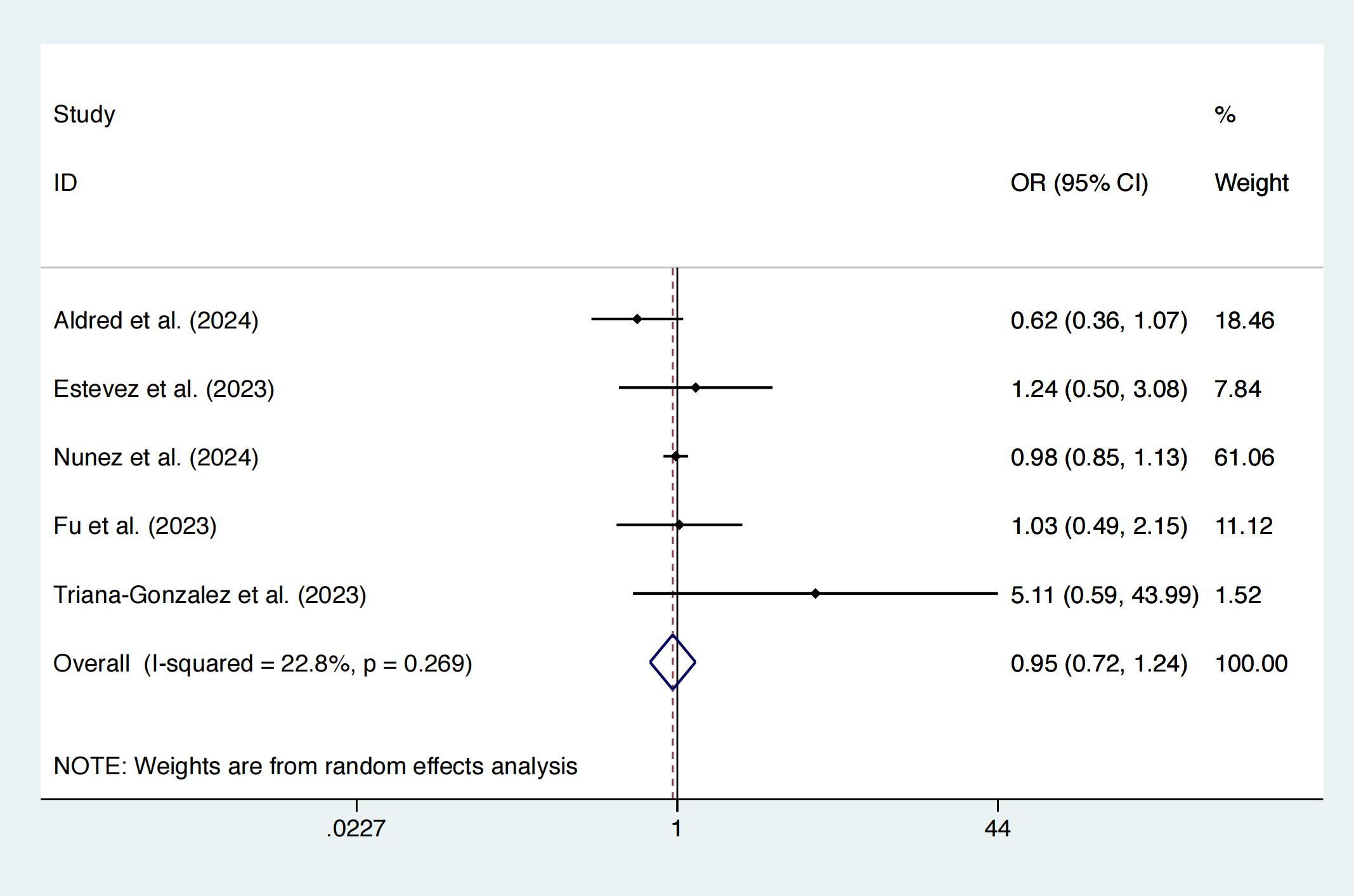
**

**Figure S55: Forest plot of differences in the type of lesions between the HIV-positive group and the HIV-negative group: papulae.**

**
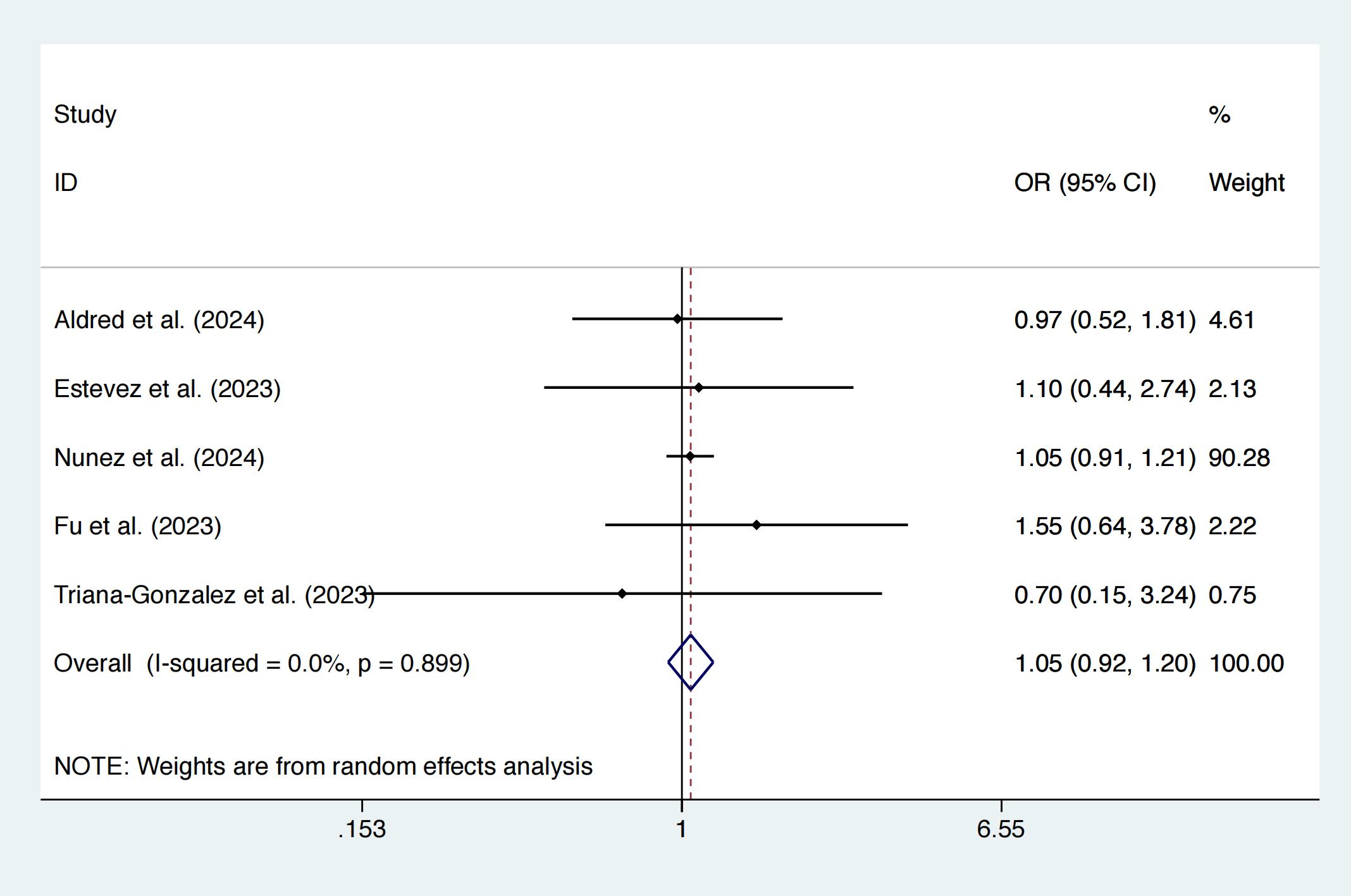
**

**Figure S56: Forest plot of differences in the type of lesions between the HIV-positive group and the HIV-negative group: vesicle.**

**
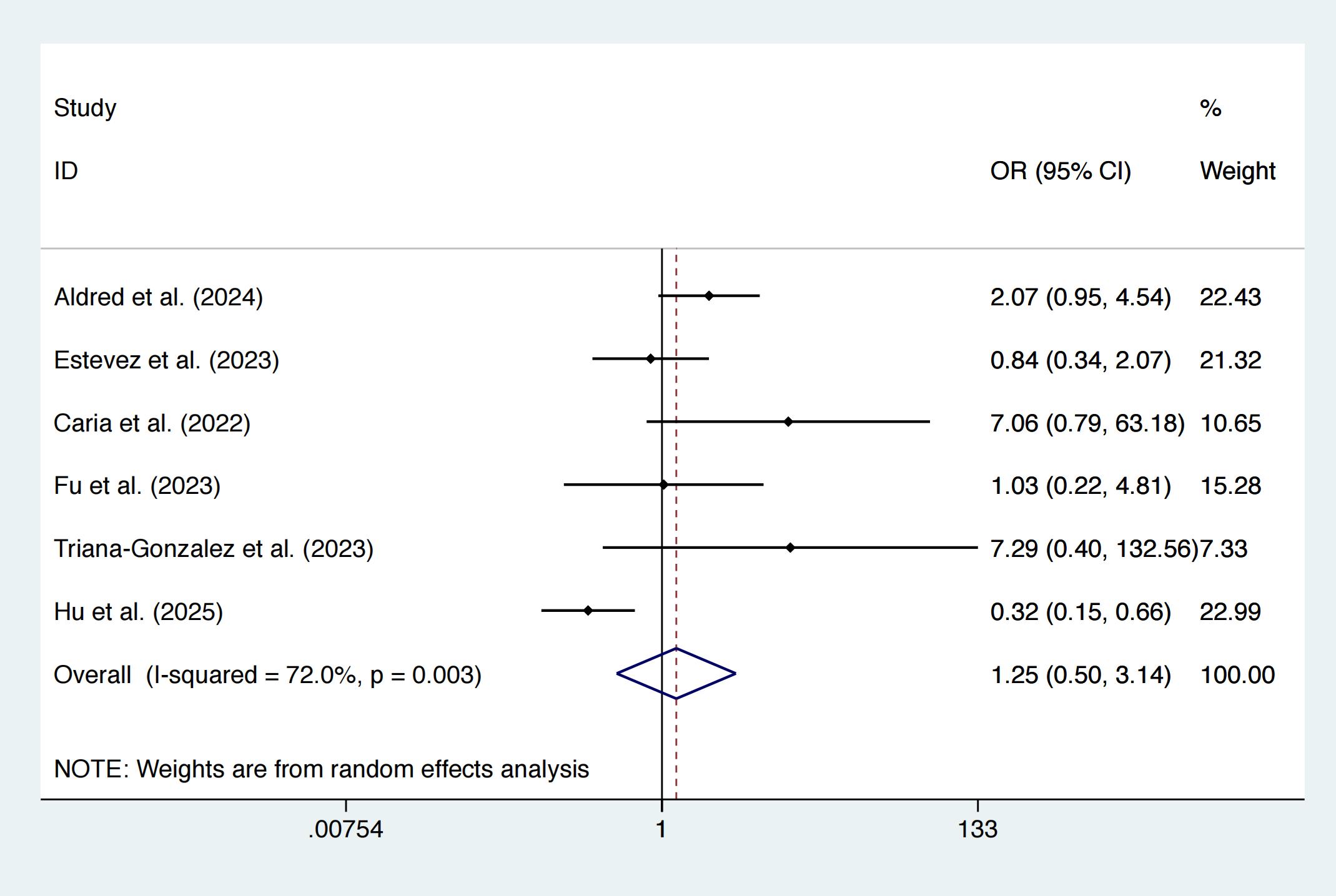
**

**Figure S57: Forest plot of differences in the type of lesions between the HIV-positive group and the HIV-negative group: ulceration.**

**
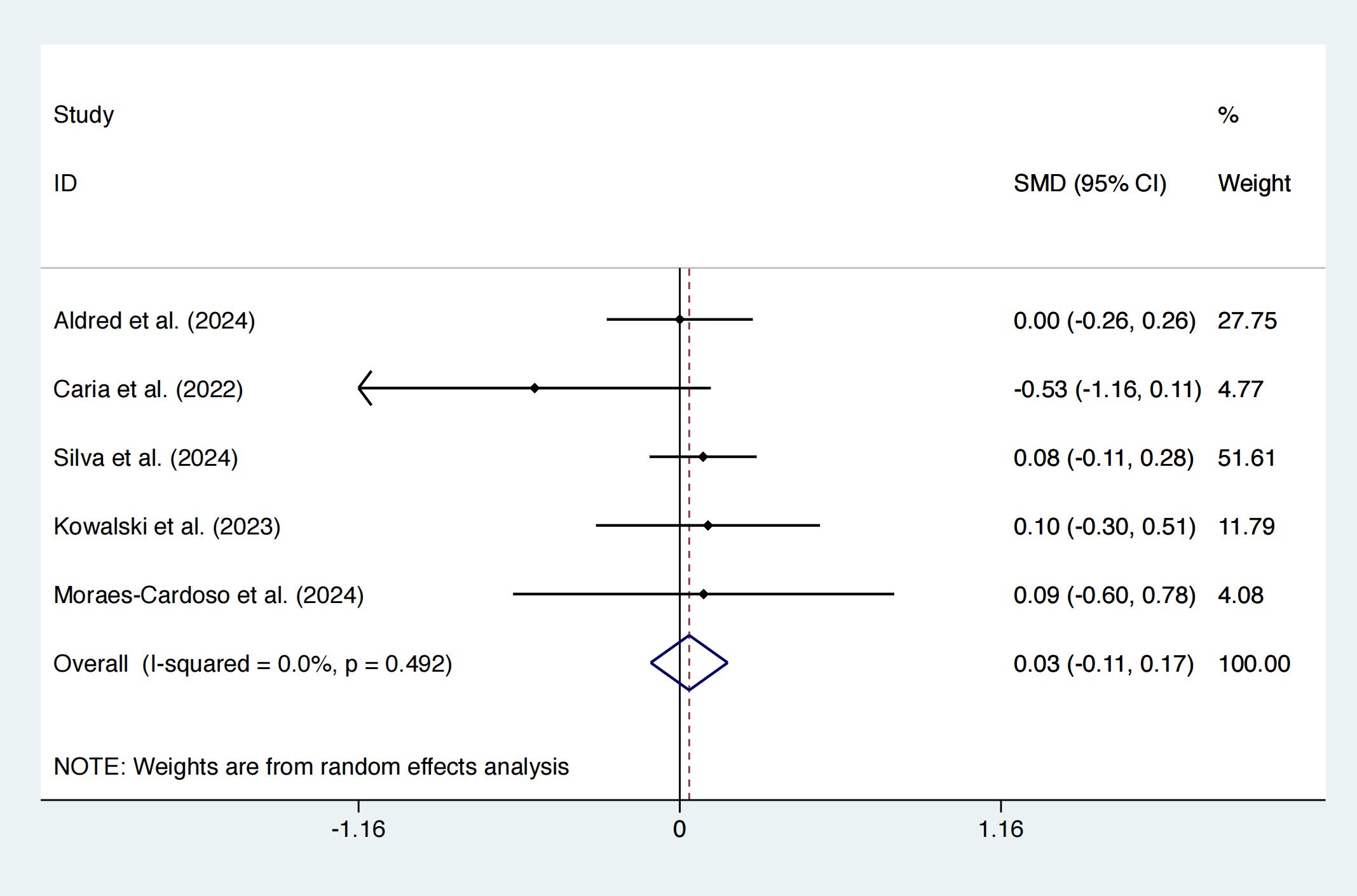
**

**Figure S58: Forest plot of differences in the clinical characteristics between the HIV-positive group and the HIV-negative group: days between first symptoms and clinical assessment.**

**Figure S59: Forest plot of differences in the clinical characteristics between the HIV-positive group and the HIV-negative group: mpox vaccination.**

**
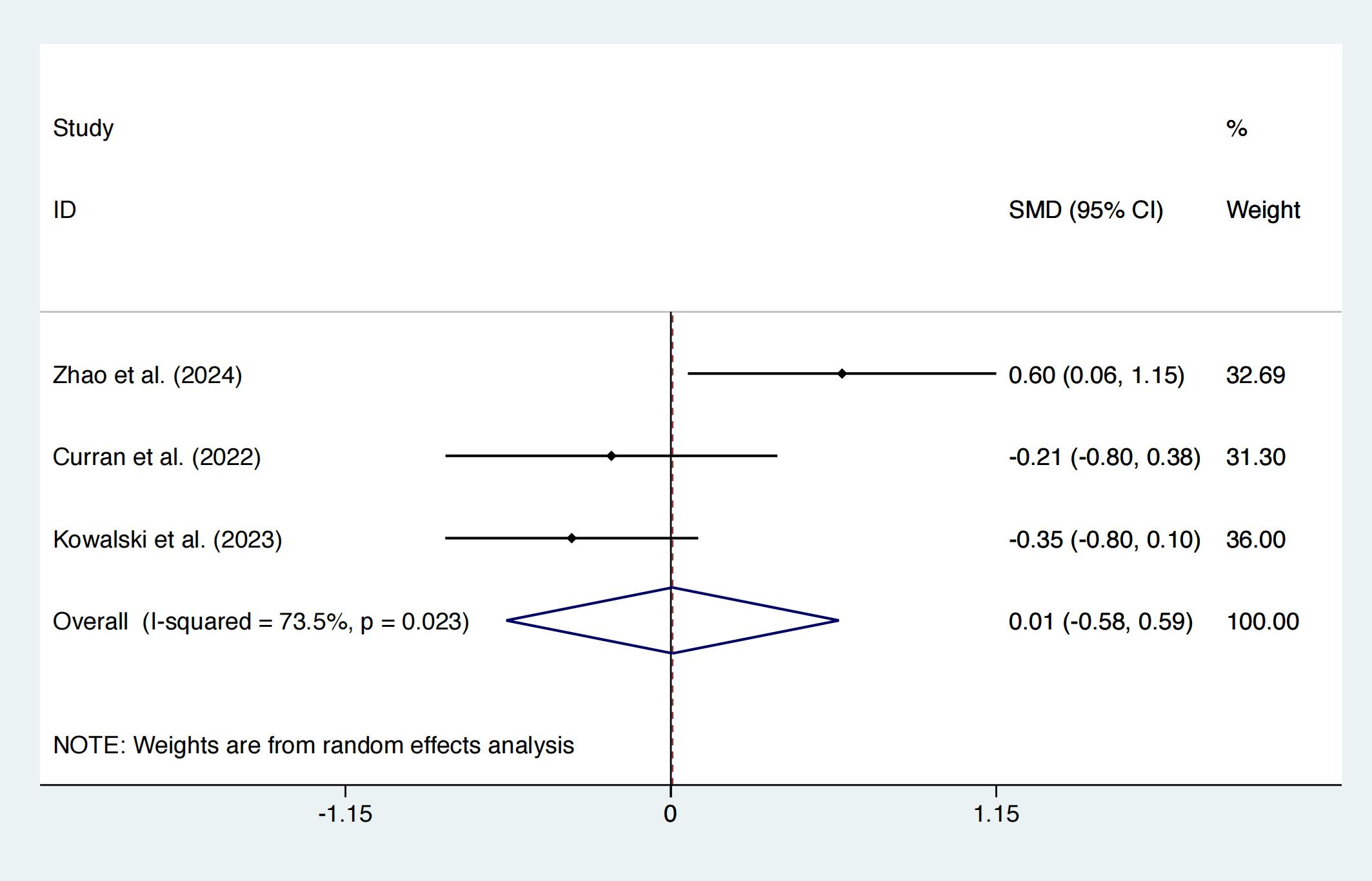
**

**Figure S60: Forest plot of differences in the clinical characteristics between the HIV-positive group and the HIV-negative group: hospitalization duration.**

**
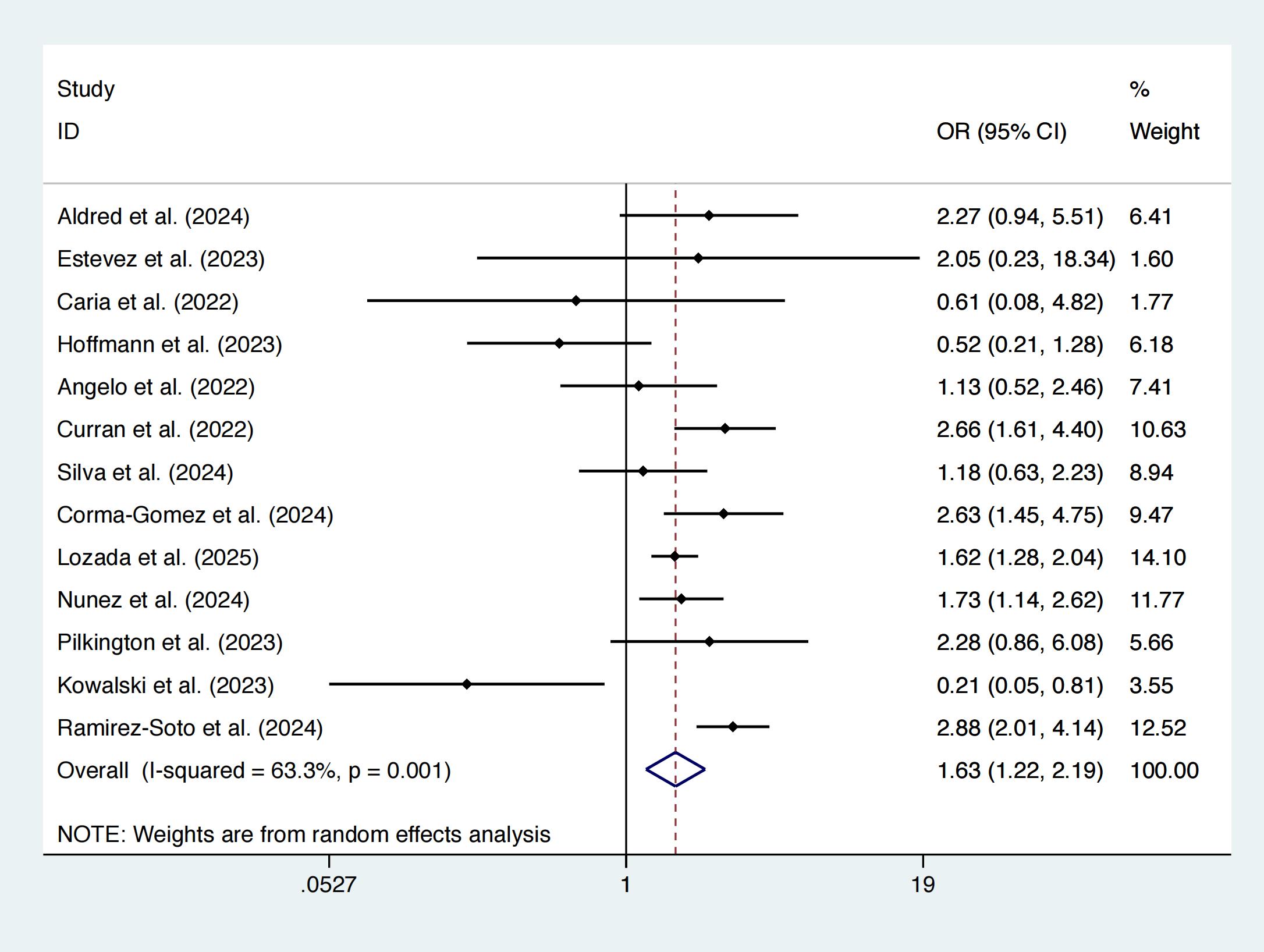
**

**Figure S61: Forest plot of differences in the clinical characteristics between the HIV-positive group and the HIV-negative group: hospitalizations.**

**Figure S62: Forest plot of differences in the clinical characteristics between the HIV-positive group and the HIV-negative group: use of tecovirimat.**

**
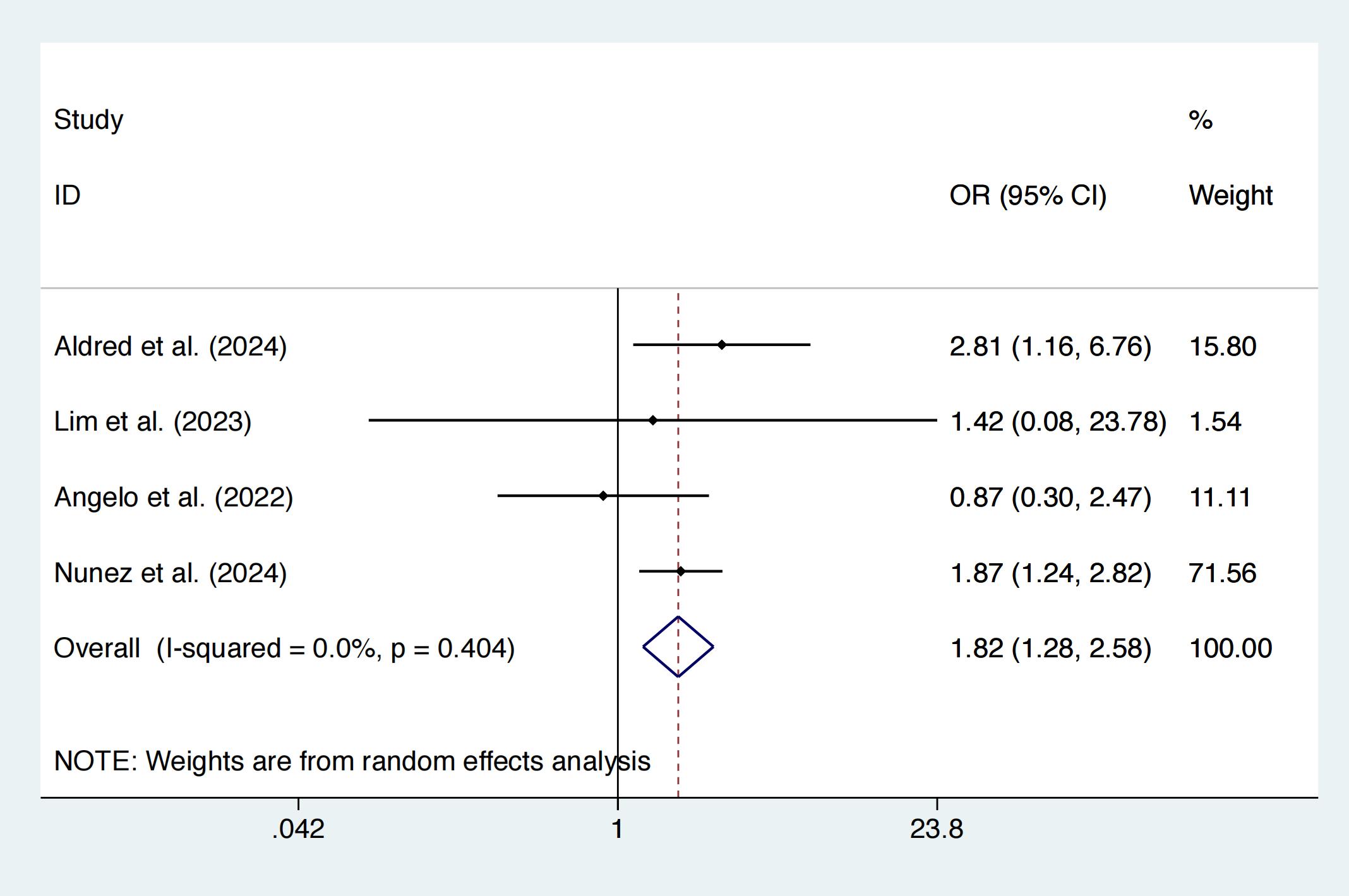
**

**Figure S63: Forest plot of differences in the clinical characteristics between the HIV-positive group and the HIV-negative group: severe mpox.**

**
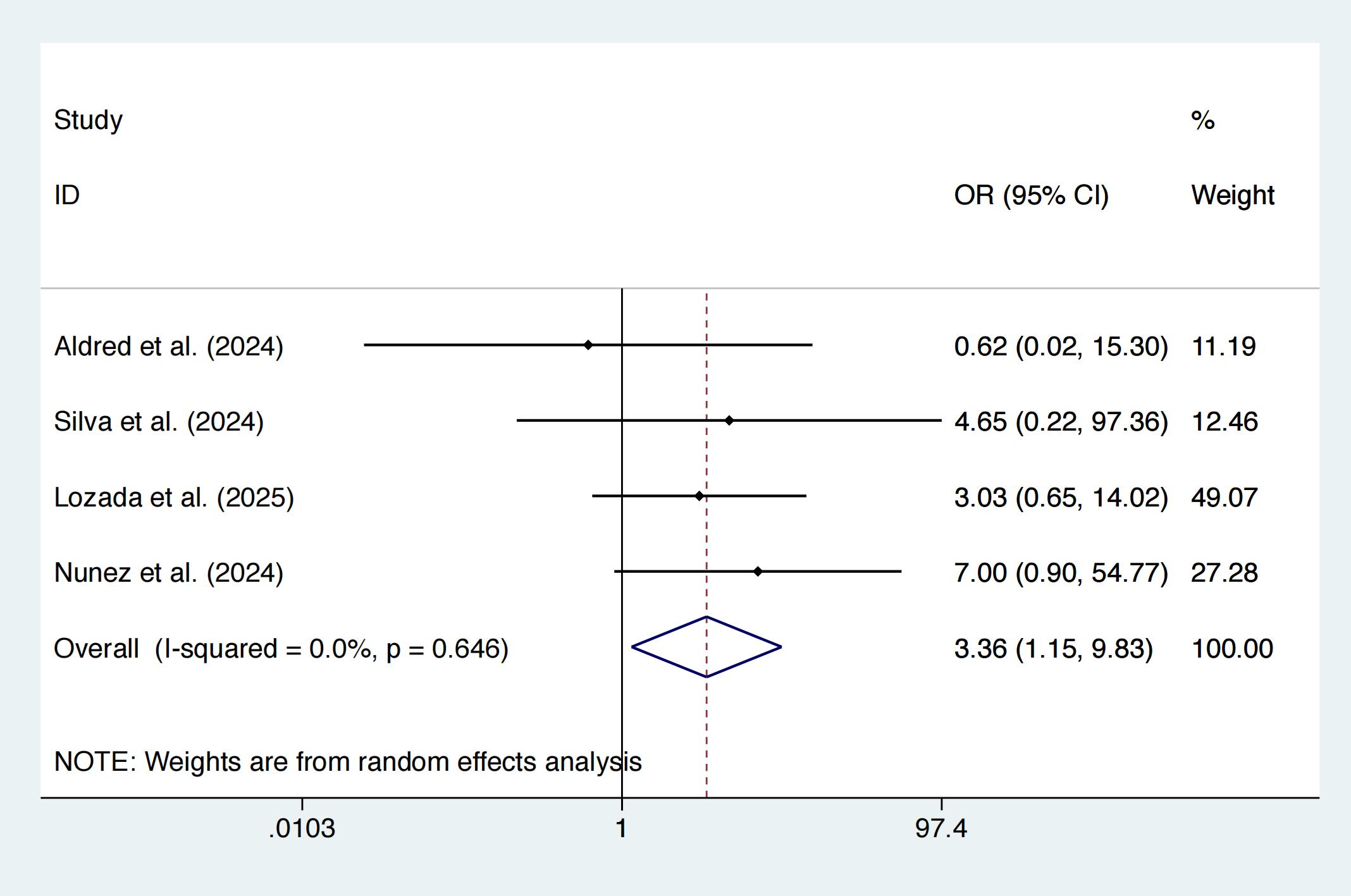
**

**Figure S64: Forest plot of differences in the clinical characteristics between the HIV-positive group and the HIV-negative group: mortality.**

**Figure S65: Egger's publication bias plot of differences in the clinical characteristics between the HIV-positive group and the HIV-negative group: age (p=0.477).**

**Figure S66: Egger's publication bias plot of differences in the clinical characteristics between the HIV-positive group and the HIV-negative group: men who have sex with men (p=0.941).**

**Figure S67: Egger's publication bias plot of differences in the race between the HIV-positive group and the HIV-negative group: Black (p=0.803).**

**Figure S68: Egger's publication bias plot of differences in the race between the HIV-positive group and the HIV-negative group: White (p=0.529).**

**Figure S69: Egger's publication bias plot of differences in the sexually transmitted infection between the HIV-positive group and the HIV-negative group: syphilis (p=0.653).**

**Figure S70: Egger's publication bias plot of differences in the sexually transmitted infection between the HIV-positive group and the HIV-negative group:** **gonorrhea (p=0.626).**

**Figure S71: Egger's publication bias plot of differences in the sexually transmitted infection between the HIV-positive group and the HIV-negative group:** **hepatitis B (p=0.085).**

**Figure S72: Egger's publication bias plot of differences in the sexually transmitted infection between the HIV-positive group and the HIV-negative group: hepatitis C (p=0.127).**

**Figure S73: Egger's publication bias plot of differences in the sexually transmitted infection between the HIV-positive group and the HIV-negative group: chlamydia (p=0.919).**

**Figure S74: Egger's publication bias plot of differences in the sexually transmitted infection between the HIV-positive group and the HIV-negative group: herpes (p=0.703).**

**Figure S75: Egger's publication bias plot of differences in the clinical characteristics between the HIV-positive group and the HIV-negative group: days between first symptoms and clinical assessment (p=0.344).**

**Figure S76: Egger's publication bias plot of differences in the location of lesions between the HIV-positive group and the HIV-negative group: palms (p=0.885).**

**Figure S77: Egger's publication bias plot of differences in the location of lesions between the HIV-positive group and the HIV-negative group: soles (p=0.290).**

**Figure S78: Egger's publication bias plot of differences in the location of lesions between the HIV-positive group and the HIV-negative group: neck (p=0.144).**

**Figure S79: Egger's publication bias plot of differences in the location of lesions between the HIV-positive group and the HIV-negative group: face/head (p=0.259).**

**Figure S80: Egger's publication bias plot of differences in the location of lesions between the HIV-positive group and the HIV-negative group: legs (p=0.282).**

**Figure S81: Egger's publication bias plot of differences in the location of lesions between the HIV-positive group and the HIV-negative group: trunk (p=0.688).**

**Figure S82: Egger's publication bias plot of differences in the location of lesions between the HIV-positive group and the HIV-negative group: oral cavity (p=0.858).**

**Figure S83: Egger's publication bias plot of differences in the location of lesions between the HIV-positive group and the HIV-negative group: genitals (p=0.149).**

**Figure S84: Egger's publication bias plot of differences in the location of lesions between the HIV-positive group and the HIV-negative group: anus (p=0.154).**

**Figure S85: Egger's publication bias plot of differences in the type of lesions between the HIV-positive group and the HIV-negative group: maculae (p=0.668).**

**Figure S86: Egger's publication bias plot of differences in the type of lesions between the HIV-positive group and the HIV-negative group: papulae (p=0.709).**

**Figure S87: Egger's publication bias plot of differences in the type of lesions between the HIV-positive group and the HIV-negative group: vesicle (p=0.890).**

**Figure S88: Egger's publication bias plot of differences in the type of lesions between the HIV-positive group and the HIV-negative group: pustules (p=0.719).**

**Figure S89: Egger's publication bias plot of differences in the type of lesions between the HIV-positive group and the HIV-negative group: ulceration (p=0.230).**

**Figure S90: Egger's publication bias plot of differences in the number of lesions between the HIV-positive group and the HIV-negative group: more than 10 (p=0.917).**

**Figure S91: Egger's publication bias plot of differences in the complications between the HIV-positive group and the HIV-negative group: proctitis (p=0.939).**

**Figure S92: Egger's publication bias plot of differences in the complications between the HIV-positive group and the HIV-negative group: bacterial infection (p=0.219).**

**Figure S93: Egger's publication bias plot of differences in the complications between the HIV-positive group and the HIV-negative group: urethritis (p=0.708).**

**Figure S94: Egger's publication bias plot of differences in the complications between the HIV-positive group and the HIV-negative group: pneumonia (p=0.474).**

**Figure S95: Egger's publication bias plot of differences in the symptoms between the HIV-positive group and the HIV-negative group: rectal pain (p=0.420).**

**Figure S96: Egger's publication bias plot of differences in the symptoms between the HIV-positive group and the HIV-negative group: fever (p=0.338).**

**Figure S97: Egger's publication bias plot of differences in the symptoms between the HIV-positive group and the HIV-negative group: lymphadenopathy (p=0.577).**

**Figure S98: Egger's publication bias plot of differences in the symptoms between the HIV-positive group and the HIV-negative group: headache (p=0.315).**

**Figure S99: Egger's publication bias plot of differences in the symptoms between the HIV-positive group and the HIV-negative group: sore throat (p=0.338).**

**Figure S100: Egger's publication bias plot of differences in the symptoms between the HIV-positive group and the HIV-negative group: asthenia (p=0.417).**

**Figure S101: Egger's publication bias plot of differences in the symptoms between the HIV-positive group and the HIV-negative group: myalgia (p=0.044).**

**Figure S102: Egger's publication bias plot of differences in the symptoms between the HIV-positive group and the HIV-negative group: diarrhea (p=0.355).**

**Figure S103: Egger's publication bias plot of differences in the symptoms between the HIV-positive group and the HIV-negative group: nausea (p=0.602).**

**Figure S104: Egger's publication bias plot of differences in the symptoms between the HIV-positive group and the HIV-negative group: arthragia (p=0.426).**

**Figure S105: Egger's publication bias plot of differences in the laboratory findings between the HIV-positive group and the HIV-negative group: white blood cell count (p=0.088).**

**Figure S106: Egger's publication bias plot of differences in the laboratory findings between the HIV-positive group and the HIV-negative group: neutrophil count (p=0.121).**

**Figure S107: Egger's publication bias plot of differences in the laboratory findings between the HIV-positive group and the HIV-negative group: lymphocyte count (p=0.875).**

**Figure S108: Egger's publication bias plot of differences in the laboratory findings between the HIV-positive group and the HIV-negative group: monocyte count (p=0.389).**

**Figure S109: Egger's publication bias plot of differences in the laboratory findings between the HIV-positive group and the HIV-negative group: platelet count (p=0.602).**

**Figure S110: Egger's publication bias plot of differences in the laboratory findings between the HIV-positive group and the HIV-negative group: hemoglobin (p=0.361).**

**Figure S111: Egger's publication bias plot of differences in the laboratory findings between the HIV-positive group and the HIV-negative group: albumin (p=0.345).**

**Figure S112: Egger's publication bias plot of differences in the laboratory findings between the HIV-positive group and the HIV-negative group: alanine transaminase (p=0.859).**

**Figure S113: Egger's publication bias plot of differences in the laboratory findings between the HIV-positive group and the HIV-negative group: total bilirubin (p=0.198).**

**Figure S114: Egger's publication bias plot of differences in the laboratory findings between the HIV-positive group and the HIV-negative group: lactate dehydrogenase (p=0.484).**

**Figure S115: Egger's publication bias plot of differences in the laboratory findings between the HIV-positive group and the HIV-negative group: creatinine (p=0.866).**

**Figure S116: Egger's publication bias plot of differences in the laboratory findings between the HIV-positive group and the HIV-negative group: procalcitonin (p=0.048).**

**Figure S117: Egger's publication bias plot of differences in the laboratory findings between the HIV-positive group and the HIV-negative group: C-reactive protein (p=0.855).**

**Figure S118: Egger's publication bias plot of differences in the laboratory findings between the HIV-positive group and the HIV-negative group: CD4^+^ T-cell count (p=0.961).**

**Figure S119: Egger's publication bias plot of differences in the laboratory findings between the HIV-positive group and the HIV-negative group: CD8^+^ T-cell count (p=0.915).**

**Figure S120: Egger's publication bias plot of differences in the clinical characteristics between the HIV-positive group and the HIV-negative group: mpox vaccination (p=0.228).**

**Figure S121: Egger's publication bias plot of differences in the clinical characteristics between the HIV-positive group and the HIV-negative group: hospitalization duration (p=0.682).**
